# Genetic Characterization of Rat Hepatic Stellate Cell Line HSC-T6 for In Vitro Cell Line Authentication

**DOI:** 10.3390/cells11111783

**Published:** 2022-05-29

**Authors:** Indrajit Nanda, Claus Steinlein, Thomas Haaf, Eva M. Buhl, Domink G. Grimm, Scott L. Friedman, Steffen K. Meurer, Sarah K. Schröder, Ralf Weiskirchen

**Affiliations:** 1 Institute of Human Genetics, Julius Maximilians University of Würzburg, D-97074 Würzburg, Germany; nanda@biozentrum.uni-wuerzburg.de (I.N.); claus.steinlein@biozentrum.uni-wuerzburg.de (C.S.); thomas.haaf@uni-wuerzburg.de (T.H.); 2 Electron Microscopy Facility, Institute of Pathology, RWTH University Hospital Aachen, D-52074 Aachen, Germany; ebuhl@ukaachen.de; 3 TUM Campus Straubing for Biotechnology and Sustainability, Technical University of Munich & Weihenstephan-Triesdorf University of Applied Sciences, D-94315 Straubing, Germany; dominik.grimm@hswt.de; 4 Division of Liver Diseases, Icahn School of Medicine at Mount Sinai, New York, NY 10029, USA; scott.friedman@mssm.edu; 5 Institute of Molecular Pathobiochemistry, Experimental Gene Therapy and Clinical Chemistry (IFMPEGKC), RWTH University Hospital Aachen, D-52074 Aachen, Germany; smeurer@ukaachen.de (S.K.M.); saschroeder@ukaachen.de (S.K.S.)

**Keywords:** liver, extracellular matrix, hepatic stellate cell, myofibroblast, fibrosis, in vitro model, SKY analysis, phalloidin stain, next generation sequencing, STR profile

## Abstract

Immortalized hepatic stellate cells (HSCs) established from mouse, rat, and humans are valuable in vitro models for the biomedical investigation of liver biology. These cell lines are homogenous, thereby providing consistent and reproducible results. They grow more robustly than primary HSCs and provide an unlimited supply of proteins or nucleic acids for biochemical studies. Moreover, they can overcome ethical concerns associated with the use of animal and human tissue and allow for fostering of the 3R principle of replacement, reduction, and refinement proposed in 1959 by William M. S. Russell and Rex L. Burch. Nevertheless, working with continuous cell lines also has some disadvantages. In particular, there are ample examples in which genetic drift and cell misidentification has led to invalid data. Therefore, many journals and granting agencies now recommend proper cell line authentication. We herein describe the genetic characterization of the rat HSC line HSC-T6, which was introduced as a new in vitro model for the study of retinoid metabolism. The consensus chromosome markers, outlined primarily through multicolor spectral karyotyping (SKY), demonstrate that apart from the large derivative chromosome 1 (RNO1), at least two additional chromosomes (RNO4 and RNO7) are found to be in three copies in all metaphases. Additionally, we have defined a short tandem repeat (STR) profile for HSC-T6, including 31 species-specific markers. The typical features of these cells have been further determined by electron microscopy, Western blotting, and Rhodamine-Phalloidin staining. Finally, we have analyzed the transcriptome of HSC-T6 cells by mRNA sequencing (mRNA-Seq) using next generation sequencing (NGS).

## 1. Introduction

Cultured primary hepatic stellate cells (HSCs) are a common in vitro model to study nearly all aspects of pro-fibrogenic liver cells. However, the usage of these cells is restricted by their limited supply, high variance of individual preparations caused by many genetic and epigenetic factors, and different ethical issues, which arise when working with primary cells. The purification procedure of these cells further requires trained personnel and specialized laboratory equipment.

Therefore, several immortalized HSC lines have been established from mouse, rat, and humans. Compared to primary cells, these cells have the advantage of growing continuously and have an almost unlimited lifespan, which is essential when performing long-lasting experiments [1]. Moreover, based on their clonal origin they have a homogenous and specific phenotype with characteristic growth properties even under non-specified culture conditions. Consequently, continuous HSC lines are attractive in vitro model systems that are widely used in biomedical research to address issues of HSC biology and function.

To date, about 30 continuous HSC lines have been established. They are typically used in investigations studying aspects of extracellular matrix synthesis, retinol metabolism, cellular toxicity, proliferation, cell adhesion, and migration. The generally high transfection efficiency of these lines further enables simple investigations using gene overexpression and silencing. Of note, researchers tend to use a specific HSC line as the ‘default’ cell line to investigate specific aspects of HSC biology. In particular, the immortalized rat liver stellate cell line HSC-T6, first established in year 1998, has become an important tool to study hepatic retinoid storage and metabolism [2,3]. Consequently, the number of published studies performed with this cell line has dramatically increased during the last year (see below). Nevertheless, permanent cell lines are prone to genotypic and phenotypic drift at higher passage numbers and are potentially vulnerable to artifacts resulting from cell misidentification or cross-contamination. Therefore, when working with a cell line, proper cell line authentication is often mandatory when publishing reliable research results or before receiving funding from granting agencies [4,5].

Specifically, three properties of a cell line need to be assessed to ensure that a cell line is consistent with the expectations [6]. Firstly, authentication through comparison of the cellular DNA profile is necessary to establish the original source of the cell line under investigation. Secondly, there is a need to test for potential contamination with organisms including bacteria, fungi, mycoplasma, yeast viruses, or cells. Thirdly, known cellular phenotypic characteristics of a cell line might be helpful to document the authenticity of a line. Typical characteristics of a cell line are doubling times, differentiation status, marker gene expression, drug sensitivity, or other genotypic or phenotypic traits [5]. Most helpful for cell authentication are short tandem repeat (STR) profiles, which facilitate reliable and quick distinction of cell lines [5]. Similarly, alteration in the karyotype including changes in the chromosomal modal number or characteristic changes to chromosome structure (deletion, inversions, and translocation) may create typical marker chromosomes by which a cell line can be identified. To date, however, genetic details have only been reported for two continuous stellate cell lines, namely human line LX-2 and murine cell line GRX [7,8], while none of the established rat HSC lines has been genetically characterized yet.

In the present study, we have performed karyotype analysis and multicolor spectral karyotyping (SKY) on HSC-T6 chromosomes, and have identified several chromosomal characteristics of the respective HSC line. Moreover, we have established a STR profile including 31 species-specific allelic variant sites that allows simple and fast cell authentication. We further performed bulk mRNA-sequencing of HSC-T6 cells cultured under basal conditions and confirmed the expression of typical HSC markers and markers relevant to retinol metabolism and storage. Rhodamine-Phalloidin staining and electron microscopy further identified robust microfilaments, cytoplasmic fat droplets, and other cellular features that are typical for a cell line originating from HSC. In summary, this study for the first time defines authentication standards for HSC-T6 cells that will be helpful in the future to provide the requested transparency and rigor when using this rat HSC line in biomedical research.

## 2. Materials and Methods

### 2.1. Literature Search

Papers working with HSC-T6 cells were identified by searching the PubMed database [9] using the search terms “HSC-T6” or “HSC-T6 and hepatic”. All papers identified by these searches are listed in Table S1.

### 2.2. Cell Culture

The adherent, immortalized rat hepatic stellate cell line HSC-T6 was first established and characterized over twenty years ago [2,3] and is commercially available since 2016 (#SCC069, Sigma-Aldrich/Merck, Taufkirchen, Germany). For our study, we have used cells at low passage numbers that were sent from the laboratory in New York at the end of the year 1999 to the laboratory in Aachen and stored frozen at −80 °C until use. The original passage numbers have not been properly recorded, as these are immortalized cells. The experiments in this study were performed at passage number pX+5 to pX+9, in which X is the unknown passage number and N is the current passage number in our laboratory. The cells were propagated in 10 cm^2^ Petri dishes and cultured in Dulbecco’s modified Eagle’s medium (DMEM) supplemented with 10% fetal bovine serum (FBS), 2 mM L-Glutamine, 1 mM sodium pyruvate, and 1 × Penicillin/Streptomycin. The medium exchange was conducted every second day and the detachment of cells for subculturing was conducted using the Accutase solution (#A6964, Sigma-Aldrich). The fibroblastic cell line WI-38 (VA-13) transformed by the oncogenic virus SV40 and was obtained from American Type Culture Collection (#CCL-75.1, ATCC, Manassas, VA, USA). The establishment and conditions used for culturing of the murine reporter stellate cell line Col-GFP HSC carrying a green fluorescent protein (GFP) expression cassette under the control of the collagen α1(I) promoter/enhancer was reported before [10]. Cells were routinely tested for mycoplasma infection by using the Venor^®^GeM OneStep Mycoplasma detection kit for conventional PCR (#11-850, Minerva Biolabs GmbH, Berlin, Germany), by essentially using the cycling conditions proposed by the manufacturer. Amplicons were separated on 2% agarose gels with ethidium bromide using 1× TAE (40 mM Tris base, 20 mM acetic acid, and 1 mM ethylenediaminetetraacetic acid disodium salt dihydrate) as a running buffer and visualized in a GEL iX20 imager (Intas Science Imaging Instruments GmbH, Göttingen, Germany) (Figure S1).

### 2.3. Electron Microscopic Analysis

The cells were fixed in 1× phosphate buffered saline (PBS) containing 3% glutaraldehyde. After washing in 0.1 M Soerensen’s phosphate buffer (Merck, Darmstadt, Germany), the samples were post-fixed in 1% osmium tetroxide (OsO_4_) (Roth, Karlsruhe, Germany) solved in 25 mM sucrose buffer (Merck) and dehydrated by ascending ethanol series (30%, 50%, 70%, 90%, and 100%) for 10 min each. The last step was repeated three times. Subsequently, dehydrated specimens were incubated in propylene oxide (Serva, Heidelberg, Germany) for 30 min, in a mixture of Epon resin (Serva) and propylene oxide (1:1) for 1 h, and finally, in pure Epon for 1 h. Epon polymerization was performed at 90 °C for 2 h. Finally, ultrathin sections (70–100 nm) were cut with an ultramicrotome (Reichert Ultracut S, Leica, Wetzlar, Germany) using a diamond knife (Diatome Ltd., Nidau, Switzerland) and picked up on Cu/Rh grids (HR23 Maxtaform, Plano GmbH, Wetzlar, Germany). Contrast was enhanced by staining with 0.5% uranyl acetate and 1% lead citrate (both Science Services, Munich, Germany). Samples were viewed at an acceleration voltage of 60 kV using a Zeiss Leo 906 (Carl Zeiss AG, Oberkochen, Germany) transmission electron microscope. Pictures were acquired at magnifications of 6000× to 100,000×.

### 2.4. Preparation of HSC-T6 Metaphase Chromosomes and Karyotyping

Chromosomes from HSC-T6 cells were prepared by a standard procedure described for metaphase preparation from rat fibroblast culture with slight modification [11]. In brief, HSC-T6 cells were allowed to grow at 37 °C in T25 flasks to semi confluent density. After exposing cells to colcemid solution (Gibco, ThermoFisher Scientific, Schwerte, Germany), cells were detached through mild trypsin-EDTA (Sigma-Aldrich) treatment and collected in a centrifuge tube. After a brief centrifugation, the cells were treated with hypotonic solution (0.56% KCl) for 30 min at 37 °C followed by fixation with a mixture of acetic acid and methanol (1:3). Air-dried chromosome spreads were prepared from the fixed cell suspension and the slides were treated with 0.025% trypsin solution at progressive intervals, followed by staining with 5% Giemsa solution to visualize the G-banding pattern. Heterochromatin staining in metaphase chromosomes was performed as previously described [12].

For conventional chromosome analysis from GTG (G-bands by trypsin using Giemsa) and CBG (C-bands by barium hydroxide using Giemsa) stained slides, at least ten metaphases were analyzed and karyotyped using the Zeiss Axio-Imager M1 microscope equipped with band view software (version 6.0; Applied Spectral Imaging Inc., Carlsbad, CA, USA). Metaphases of at least >350 band resolutions were photographed, and the chromosome identification and karyotyping was achieved according to a published report [11].

### 2.5. In Situ Hybrdization and Spectral Imaging

Commercially available rat SKY probe (Applied Spectral Imaging Inc., Carlsbad, CA, USA) was used to detect potential rearrangements in HSC-T6 chromosomes. Denaturation of the SKY probe and HSC-T6 chromosome spreads, as well as the conditions used for in situ hybridization, 4′,6-diamidino-2-phenylindole (DAPI) counterstaining, anti-fade embedding, and spectral imaging and analysis, was conducted essentially following a protocol described before [13].

### 2.6. Short Tandem Repeat (STR) Profiling

STR profiling and interspecies contamination test for HSC-T6 cells was performed using the cell line authentication service from IDEXX (Kornwestheim, Germany) using the CellCheck Rat system. This system establishes a genetic cell profile using 31 species-specific STR markers.

### 2.7. Next Generation Sequencing and Data Analysis

High quality RNA from HSC-T6 cells was isolated by an established procedure using CsCl density gradient centrifugation [8]. In brief, confluent cells were lysed and homogenized in a guanidine thiocyanate-containing buffer. Subsequently, the solution was layered onto a cesium chloride cushion and centrifuged for 21 h at 21 °C and 25,000 rpm in a Beckman SW41 rotor. The resulting pellet was resuspended in sterile water, once purified by ethanol precipitation, and finally resuspended in sterile water. Concentration, purity, and quality were determined by UV spectroscopy and on the Agilent 4200 TapeStation automated platform (Agilent Technologies Inc., Waldbronn, Germany). Subsequently, the ribosomal RNA was depleted and the mRNA was converted into a library of template molecules suitable for subsequent cluster generation and DNA sequencing using the NEBNext Multiplex Oligos for Illumina Index Primers Set 1 kit containing preformed adaptors and primers. Pre-filled ready-to-use-cartridges (MiSeq Reagent kit V2, 300-cycles; Illumina Inc., San Diego, CA, USA) were used for sequencing on the Illumina platform (Illumina). All sequencing results were converted into fastq datafiles. Construction and sequencing of the cDNA library was conducted in the IZKF Genomic Facility of the University Hospital Aachen.

Before any downstream analysis, all sequencing reads were first quality controlled using FastQC (v0.11.8) [14]. Sequencing adapters and low-quality ends (with a cutoff of 20) have been trimmed from all reads as well as reads with a length below 35-bp were removed using the tool cutadapt (v1.9.1) [15]. Next, kallisto (v0.48.0), a pseudoalignment-based RNASeq quantification tool, was used to quantify transcript abundances [16]. For this purpose, the latest *Rattus norvegicus* Rnor_6.0 reference transcriptome [17] and transcriptome information files [18] were downloaded from Ensembl [19]. In addition, bootstrapping with 100 bootstraps was used for quantifying the abundances of transcripts. Transcript abundances are reported in Transcripts Per Million (TPM).

### 2.8. Western Blot Analysis

Preparation of protein extracts, protein quantification, and Western blot analysis were conducted using the established protocols published previously [20]. In brief, equal amounts of protein cell extracts (40 µg/lane) from HSC-T6 and WI-38 (VA-13) or brain tissue (60 µg/lane) were heated at 80 °C for 10 min and separated in 4–12% Bis-Tris gels (Invitrogen, Darmstadt, Germany) under reducing conditions using the MES running buffer. Proteins were subsequently electro-blotted on nitrocellulose membranes (Schleicher & Schuell, Dassel, Germany). Proper transfer to the membranes was controlled by Ponceau S stain and unspecific binding sites were blocked in TBST (10 mM Tris/HCl, 150 mM NaCl, 0.1% (*v*/*v*) Tween 20, pH 7.6) containing 5% (*w*/*v*) non-fat milk powder. The membranes were probed with antibodies specific for Fibronectin (#AB1954, Sigma-Aldrich), collagen type I (#NB600-408, Novus Biologicals, Wiesbaden Nordenstadt, Germany), Vimentin (#ab92547, Abcam, Berlin, Germany), SV40 large T antigen (#sc-20800, Santa Cruz Biotech., Santa Cruz, CA, USA), α-smooth muscle actin (#CBL171-1, Sigma-Aldrich), GFAP (clone 2E1, #sc-33673, Santa Cruz), Ferrtin heavy chain (clone B-12, #sc-376594, Santa Cruz), PDGF-Rβ (#sc-432, Santa Cruz), TGF-βRII (#sc-400, Santa Cruz), pSmad2CT (#CS8828, Cell Signaling Technology, Frankfurt am Main, Germany), total Smad2 (#CS3103, Cell Signaling), CTGF (#sc-14939, Santa Cruz), ppERK1/2 (#CS9101, Cell Signaling), Id2 (#sc-489, Santa Cruz), collagen Iα1 (#PS065, Monosan, Biomol, Hamburg, Germany), GAPDH (sc-32233, Santa Cruz), and β-actin (#A5441, Sigma-Aldrich), respectively. Primary antibodies were detected with horseradish-peroxidase (HRP)-conjugated secondary antibodies and the Supersignal™ chemiluminescent substrate (Perbio Science, Bonn, Germany).

### 2.9. Rhodamine-Phalloidin Stain

For microfilament staining, 40,000–60,000 cells/well were seeded in 24-well plate on glass coverslips. After 24 h, the medium was removed and the cells were washed three times in PBS and fixed in 3.7% neutral buffered paraformaldehyde for 20 min. Subsequently, the cells were washed again three times with PBS and then permeabilized with precooled 0.1% sodium citrate/0.1% Triton-X-100 solution for 3 min. The cells were further washed three times with PBS and unspecific binding sites were blocked by incubation in PBS containing 50% (*v*/*v*) FBS and 0.5% (*w*/*v*) bovine serum albumin for 1 h at room temperature. A total of 5 µL of a Rhodamine-Phalloidin stock solution (#R415, ThermoFisher Scientific Inc., Waltham, MA, USA) was diluted in 200 µL PBS containing 1% (*w*/*v*) BSA. The solution was applied on each sample and incubated for 20 min at room temperature in the dark. Finally, the stained samples were washed three time in PBS, nuclei counterstained by incubation with 500 µL 4’,6-diamidino-2-phenylindole (DAPI) solution (200 ng/mL, #D1306, ThermoFisher Scientific Inc.) for 30 min at room temperature in the dark, again followed by washing three times in PBS and once in dH_2_O. For documentation, the glass coverslips were mounted with PermaFluor aqueous mounting medium (#TA-030-FM, ThermoFisher Scientific Inc.) and analyzed under a Nikon Eclipse E80i fluorescence microscope equipped with the NIS-Elements Vis software (version 3.22.01).

### 2.10. Stimulation with Fatty Acids and Lipid Droplet Stain

Stimulation of HSC-T6 with oleic acid and subsequent staining with BODIPY^TM^ 493/503 to visualize lipid droplets was performed according to published protocols [21,22]. In brief, the cells were split on glass coverslips, which were placed into 6-well plates. After 24 h, the cells were stimulated by the addition of oleic acid (#O3008, Sigma-Aldrich) to a final concentration of 0.2 mM. HSC-T6 grown in standard medium containing the vehicle served as controls. After 24 h, the cells were fixed in 3.7% neutral buffered paraformaldehyde. Subsequently, the cells were washed with PBS and then permeabilized with precooled 0.1% sodium citrate/0.1% Triton-X-100 solution for 3 min. For lipid droplet staining, BODIPY^TM^ 493/503 (#D3922, Invitrogen) was dissolved in DMSO (5 mM stock). In all further steps, the samples were constantly protected from light. After washing again with PBS, the cells were stained under shaking in a 6 µM BODIPY^TM^ 493/503 solution (prepared in PBS) for 1 h at room temperature. After staining, the samples were washed again with PBS. Finally, the slides were counterstained with DAPI and mounted using a PermaFluor aqueous mounting medium (ThermoFisher Scientific Inc.) and examined using a Nikon Eclipse E80i microscope.

### 2.11. Stimulation of HSC-T6 Cells

HSC-T6 cells were cultured in growth medium until confluent. For stimulation, the cells were detached using 1× Trypsin and plated in 6-well dishes at a density of 4 × 10^5^ cells per well. Cells were cultured overnight in growth medium, which was exchanged to starvation medium with reduced FBS content (0.5% FBS) 2 h before the experiment. When indicated, the ALK5 inhibitor SB-431542 (#S4317, Sigma-Aldrich) was added to the medium 30 min prior to the ligand application. Recombinant mouse TGF-β1 (mTGF-β1, #7666-MB, R&D Systems, Wiesbaden, Germany) at 1 ng/mL or recombinant human PDGF-BB (hPDGF-BB, #220-BB; R&D Systems) at 25 ng/mL was added to the cells for indicated times. Thereafter, cells were lysed in: (i) RIPA lysis buffer and prepared for the Western blot analysis, as outlined above, or (ii) in the Lysis buffer of the PureLink^TM^ RNA Mini kit (#12183018A, Invitrogen) supplemented with 40 mM dithiothreitol (DTT) for qPCR analysis. For the Western blot analysis, 40 µg (30 min stimulation experiment) or 60 µg protein extracts (24 h stimulation) were used. For RT-qPCR, 1 µg of total RNA was reverse transcribed using the SuperScript^TM^II Reverse Transcriptase (#18064014, Invitrogen) and the first strand cDNA reaction was diluted 1:10 and used for subsequent PCR analysis.

### 2.12. Reverse Transcription Polymerase Chain Reaction

Reverse transcription of RNA isolated from HSC-T6 cells was conducted according to standard procedures [8]. All primers used in the study are given in Table S2.

## 3. Results

### 3.1. Usage of HSC-T6 Cells in Biomedical Research

**Figure 1:**
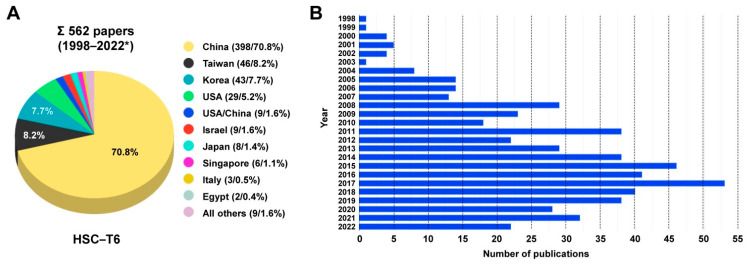
Studies of HSC-T6 cells in articles published during 1998-2022. (**A**) Studies using HSC-T6 cells were identified by searching the PubMed database or using the Google search engine using the search terms “HSC-T6” or “HSC-T6 and hepatic”. The search conducted on 6 May 2022 resulted in a total of 562 papers that reported findings established in HSC-T6 cells. Most studies with these cells were conducted in China, Taiwan, and Korea. (**B**) Since the establishment of HSC-T6 cells, reports using this cell line as an experimental tool steadily increased from year to year. * Please note that papers for 2022 only cover the time interval from January to April.

To date, there are 10 HSC lines available that were either established from the Wistar or Sprague-Dawley rat, namely CFSC (and subclones derived thereof) [22,23], NFSC [23,24], PAV-1 [25], HSC-PQ [26], BSC (and subclones derived thereof) [27,28], MFBY2 [29], T-HSC/Cl6 [30], LSC-1 [31], RGF (and subclone RGF-N2) [32], and HSC-T6 [2,3]. However, none of these lines has been thoroughly characterized for genetic details yet. This is somewhat surprising, since a detailed search revealed that there are more than 550 studies that have already published results with this cell line (Figure 1, Table S1).

### 3.2. Phenotypic Appearance of HSC-T6 Cells

**Figure 2:**
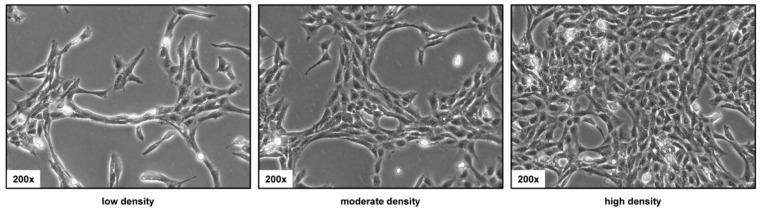
Light microscopic appearance of HSC-T6 cells. The cells were seeded in cell culture dishes and representative images taken at different cell densities. Original magnifications are 200×.

Cultured on uncoated plastic, HSC-T6 have a typically spindle-shaped morphology. However, these phenotypic characteristics might change in response to cell density (Figure 2). At low density, the cells have an elongated morphology, while at higher density, the cells form a dense network in which the individual cells look smaller.

### 3.3. Electronmicroscopic Analysis of HSC-T6 Cells

**Figure 3:**
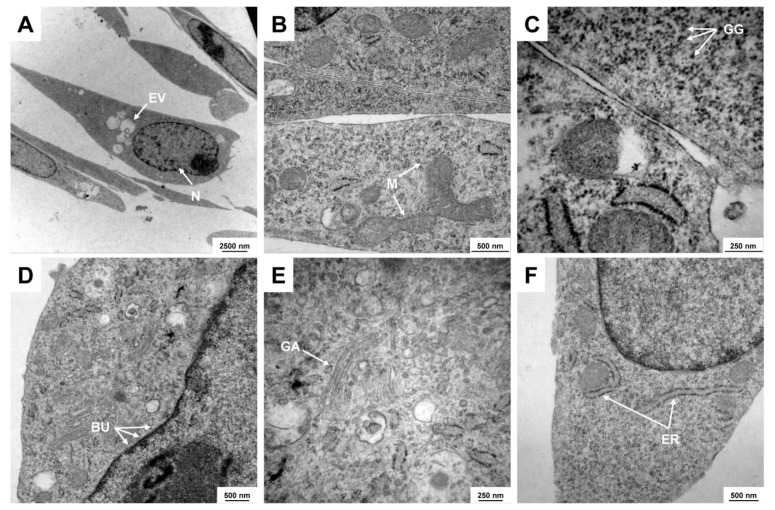
Representative electron microscopic analysis of HSC-T6 cells. Typical ultrastructural features of HSC-T6 cells are depicted including (**A**) the large cell nucleus (N) and endolysosomal vesicles (EV), (**B**) large mitochondria (M), (**C**) typical colloidal spherical glycogen granules (GG), (**D**) excessive bulges (BU) of the nuclear envelope, (**E**) pronounced Golgi apparatus (GA), and (**F**) rough endoplasmic reticulum (ER). Magnifications: (**A**) 3597×, (**B**) 27,800×, (**C**) 60,000×, (**D**) 16,700×, (**E**) 35,970×, and (**F**) 21,560×, respectively.

HSC-T6 cells have several specific ultrastructural features typical of hepatic stellate cells. In particular, the cells are characterized by a typical large nucleus with excessive bulges of the nuclear envelope, prominent endolysosomal vesicles, large mitochondria, glycogen granules, prominent Golgi apparatus, and rough endoplasmic reticulum, which can be visualized using transmission electron microcopy (Figure 3).

### 3.4. Expression of Typical Hepatic Stellate Cell Markers in HSC-T6 Cells

**Figure 4:**
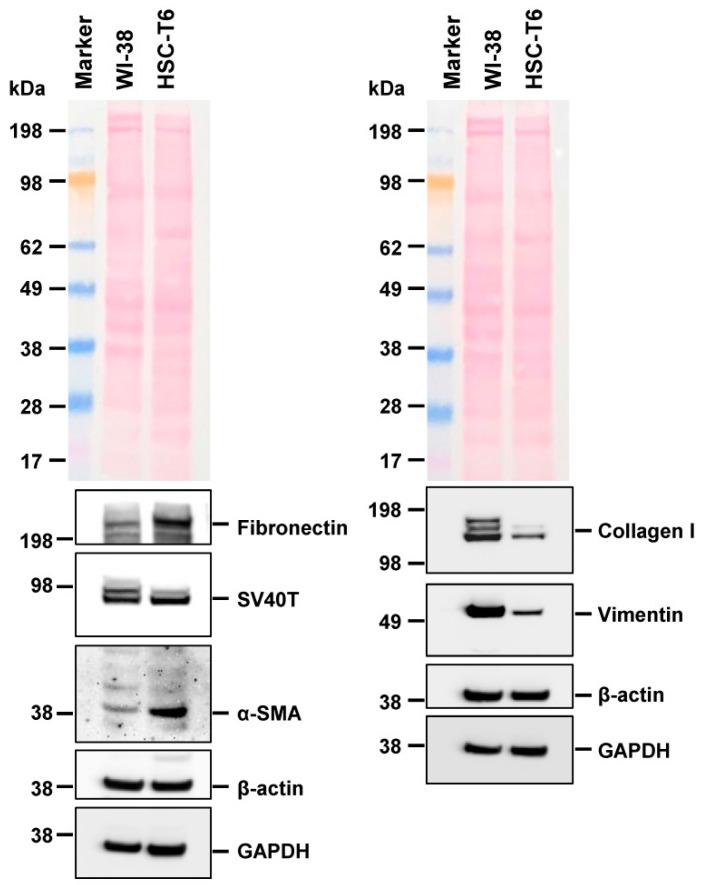
Hepatic stellate cell marker expression in HSC-T6 cells. Cell extracts were prepared from WI-38 and HSC-T6 cells and analyzed for expression of Fibronectin, Simian virus (SV40) large T antigen (SV40T), α-smooth muscle actin (α-SMA), collagen type I, and Vimentin. The expression of β-actin, GAPDH, and Ponceau S stain were included to demonstrate equal protein loading.

In line with the original report describing the establishment of line HSC-T6 from Sprague-Dawley rats [2,3], the cells grow rapidly in the culture and express the cytoskeletal proteins α-smooth muscle actin (α-SMA) and Vimentin that are typical for activated stellate cells (Figure 4). In addition, the cells are positive for Fibronectin and collagen type I, which also support the notion that these cells originated from pro-fibrogenic HSC. Importantly, the cells still express the large T-antigen of SV40 (SV40T), which was used as the oncogenic agent for immortalization of HSC-T6 [2,3]. In addition, we demonstrate that HSC-T6 cells express low quantities of the glial fibrillary acid protein (GFAP) (Figure S2). Interestingly, the basal expression of collagen type Iα1 (*ColIa1*) was significantly higher than in other continuous HSC lines such as Col-GFP HSC and not stimulated by the transforming growth factor-β1 (TGF-β1) (Figure S3A). Conversely, the basal expression of α-SMA (*Acta2*) was significantly lower in HSC-T6 than in Col-GFP HSC, and increased after stimulation with TGF-β1 (Figure S3B). Another typical profibrogenic marker, the connective tissue growth factor (*Ctgf*/*Ccn2*), was only slightly induced by TGF-β (Figure S3C), while the relative mRNA abundances of the transforming growth factor-β type II (*Tbr2*), *Tgfb1*, and *Gfap* were not altered after stimulation with the respective cytokine (Figure S3D). However, the cells are highly responsive to TGF-β1 as assessed by the stimulation of Smad2 phosphorylation after short term stimulation for 30 min (Figure S4). Moreover, HSC-T6 demonstrated increased expression of CTGF/CCN2 after longer term stimulation with TGF-β1 for 24 h (Figure S5).

**Figure 5:**
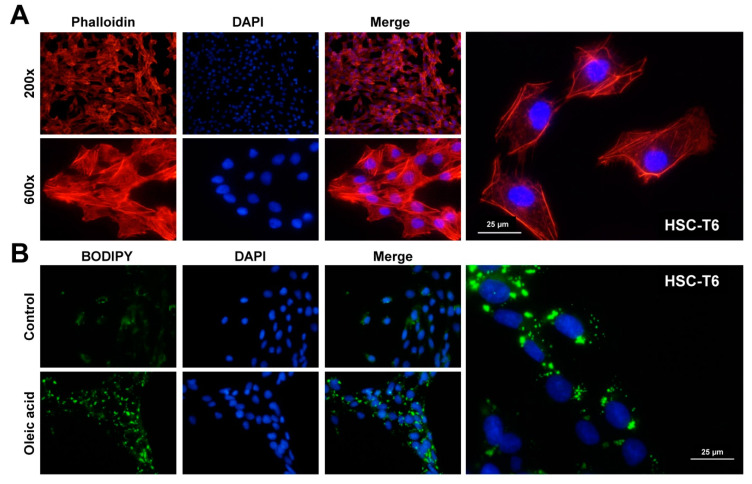
Microfilament staining and uptake of fatty acids in HSC-T6 cells. (**A**) HSC-T6 cells were seeded on glass coverslips and stained with Rhodamine-Phalloidin. The stain revealed a dense network of microfilaments in the cytoplasm. (**B**) HSC-T6 were grown in the presence of oleic acid and stained with BODIPY^TM^ 493/503, as control cells that were cultured in the presence of the vehicle were stained in parallel. Nuclei in (**A**) and (**B**) were counterstained with 4’,6-diamidino-2-phenylindole (DAPI). (**B**) All glass coverslips were mounted with PermaFluor aqueous mounting medium and analyzed under a fluorescence microscope at indicated magnifications.

In addition to these cellular characteristics, cultured HSC-T6 cells form robust intracellular actin microfilaments (Figure 5A), which are the basis for the acquisition of their contractility, migration, and adhesion properties, as reported previously [33,34]. Similarly, the cells have capacity to incorporate fatty acids into cytosolic lipid droplets when cultured in media containing the monounsaturated fatty acid oleic acid (C18:1) (Figure 5B).

To analyze gene expression across the transcriptome, we next performed bulk mRNA sequencing (mRNA-Seq) from HSC-T6 cells using next generation sequencing (NGS). For this analysis, total RNA was isolated from cells that were grown to approximately 80% confluence in basal medium. The transcript abundances of mRNAs obtained by NGS analysis were then quantified by using the information about the latest *Rattus norvegicus* reference transcriptome. The complete list of mRNA abundances is given in Table S3. We found in total 22,056 different transcript species that had a transcript abundance that were in the range of 4.8 × 10^−11^ (ENSRNOT00000000821.6, *Acab*, and acetyl-CoA carboxylase beta) and 9890.55 (ENSRNOT00000030919.5, *Fth1*, and ferritin heavy chain 1), respectively. Genes that are known to be typically expressed in HSC, including α-smooth muscle actin (*Acta2*), collagen type I α1 (*Col1a1*), collagen type III α1 (*Col3a1*), vimentin (*Vim*), fibronectin 1 (*Fn1*), galectin 1 (*Lgals1*), secreted protein acidic and cysteine rich (*Sparc*), and the cysteine and glycine-rich protein 2 (*Csrp2*), were found in an abundance of 2.377 (*Acta2*) and 4801.86 (*Lgals1*) (Table 1). Similarly, we found a strong expression of *Timp1*, *Timp2*, and *Cd63*, which encodes a TIMP1 receptor. The smooth muscle marker tansgelin 2 (*Tagln2*) and cytoskeleton-associated proteins, such as cofilin 1 (*cfl1*), β-actin (*Actb*), and caveolin 1 (*Cav1*), were highly abundant in HSC-T6 cells. Most importantly, the expression of several nuclear receptors and genes associated with retinoid metabolism were detected, as demonstrated previously in the initial descriptions of HSC-T6 [2,3]. Although we have not confirmed the expression of the respective retinoid receptors at the protein level, our NGS analysis confirms the previous study, which analyzed the expression of respective receptor in more detail [3]. Strikingly, the expression of ferritin heavy chain 1 (*Fth1*), which is fundamentally involved in iron homeostasis, had the highest mRNA abundance in HSC-T6, with a TPM of nearly 10,000. The expression of *Fth1* in HSC-T6 was confirmed at both mRNA and protein levels (Figure S6).

Moreover, the antiviral response protein bone marrow stromal cell antigen 2, also known as tetherin, that was recently also found in murine HSC line GRX [8], was expressed in large quantities in HSC-T6 cells.

### 3.5. Convential Genetic Analysis of HSC-T6 Cells

**Figure 6:**
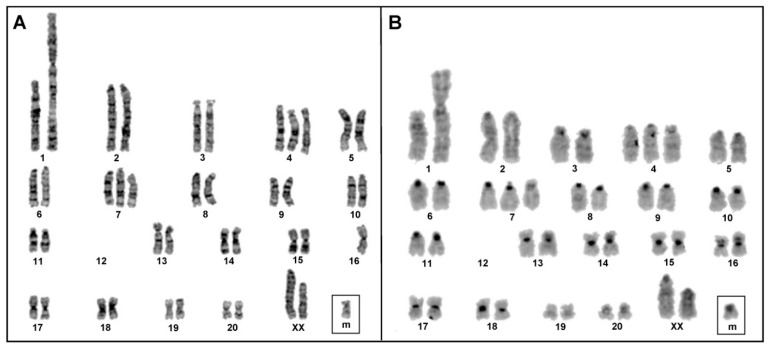
Conventional cytogenetic analysis of HSC-T6 cells. (**A**) GTG banded and (**B**) CBG-banded karyotypes of HSC-T6 showing structural and numerical aberrations. Note that one copy of chromosome 16 was missing in the metaphase spreads (**A**), probably due to overspreading of chromosomes.

Analysis from at least 20 Giemsa-stained metaphases revealed that the chromosome number of HSC-T6 ranges from 40–44. Similar numerical variation was encountered in metaphases harvested at different times. In the majority of metaphases, however, the chromosome number was consistently found to be 43. The GTG banded karyotype apparently demonstrated that one of the homologs of chromosome 1 (RNO1) is extremely metacentric and appears to be a derivative chromosome (Figure 6A). In addition, the banding pattern characteristically identified both chromosomes 4 and 7 (RNO4 and RNO7) in three copies. Notably, our analysis failed to identify the complete chromosome 12 (RNO12) but was able to detect a small marker chromosome (Figure 6A). The band resolution is not adequate enough to verify whether the derivative chromosome 1 or the marker chromosome contains part of the RNO12. The CBG banded karyotype (Figure 6B) rules out any major rearrangements involving the centromeric regions and confirms that HSC-T6 is a female cell line through the absence of typical heterochromatic Y. The conventional chromosome analysis demonstrates the non-random accumulation of both structural and numerical rearrangements. Although the insertions or cryptic rearrangements cannot be evaluated through the banding pattern, based on the GTG analysis, the karyotype of HSC-T6 can be depicted provisionally as 43,XX,der(1),+4,+7,-12,+mar.

### 3.6. In Situ Hybridization and Spectral Karyotype Analysis

**Figure 7:**
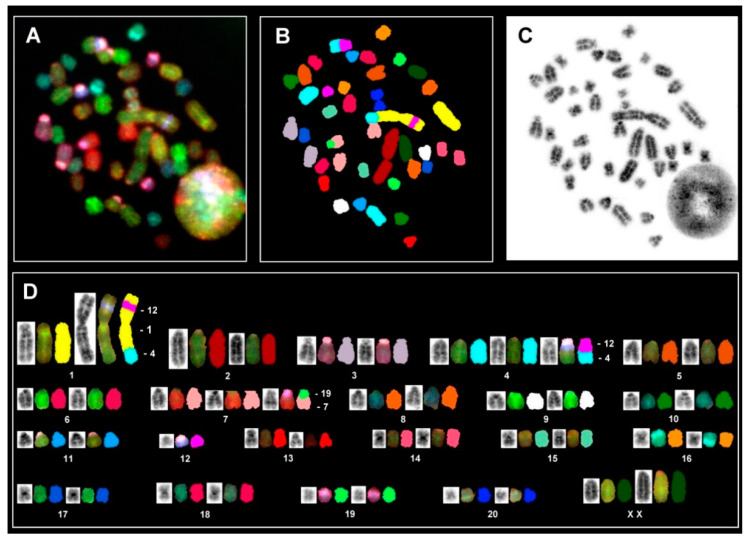
Spectral karyotyping of a HSC-T6 cell line metaphase. (**A**) RGB image after hybridization of metaphase with the SKY probe cocktail. (**B**) Classified pseudo-color image of the same metaphase. (**C**) Inverted DAPI-stained image. (**D**) Karyotype of the metaphase showing spectrally classified, pseudo-colored chromosomes (right) compared with its inverted DAPI-stained chromosomes (left) and corresponding RGB image (middle). Specific chromosomes involved in rearrangements are indicated.

Based on the assigned specific color code to individual chromosomes, the SKY analysis allows for the authentic identification of both structural and numerical aberrations. Figure 7 displays metaphase spread and karyotypes exhibiting clear SKY hybridization signals, specifying individual chromosomes along with information about structural aberrations.

Interestingly, the large derivative chromosome 1 that cannot be characterized through its banding pattern contains a part of RNO4 at the distal region of one arm and the other arm shows a segment interstitially from the RNO12. The hybridization patterns generally display two identical hybridization signals for each chromosome pair. On the other hand, three hybridization signals were observed for RNO4 and RNO7, indicating the presence of three copies of these two different chromosomes. However, one of these three copies of both chromosomes is strikingly rearranged in all metaphases. The proximal part of the RNO4 contains a part of RNO12, whereas the proximal region of RNO7 displays a signal specific to RNO19. Since the signal specific to RNO12 is also present interstitially on the derivative chromosome (RNO1), it may be assumed that one complete homolog of RNO12 has accumulated a break followed by the transposition to two different chromosomes (RNO1 and RNO4). This may point to four independent translocations in the HSC-T6 karyotype involving RNO1, RNO4, and RNO12 and next involving RNO7 and RNO19. Since the SKY karyotype was able to detect a single RNO12, it is likely that the marker chromosome identified in the banded chromosomes is actually RNO12. Although the hybridization signals clearly detect three copies of both RNO4 and RNO7, a possible partial trisomy for RNO12 or RNO19 cannot be ruled out. Apart from these major recurrent rearrangements, Table 2 presents 11 different translocations encountered in 20 different metaphases karyotyped. Since each of these 11 rearrangements was found in no more than two metaphases, these aberrations may be considered as random events.

Based on the SKY analysis, the karyotype of HSC-T6 can be outlined as 43,XX,der(1)t(1;4)ins(1;12),der(4)t(4;12),+4,der(7)t(7;19),+7,(?)+12,(?)+19, respectively.

### 3.7. Short Tandem Repeat Analysis

The karyotype analysis demonstrated that HSC-T6 cells contain non-random accumulation of both structural and numerical rearrangements. To further establish a simple characteristic useful to uniquely identify HSC-T6 cells, short tandem repeat (STR) DNA profiling was performed using 31 polymorphic markers. The resulting PCR products were analyzed on a genetic analyzer resulting in electropherogram profiles with characteristic peaks for each highly-polymorphic STR tested (Figure S7) that allowed one to define a characteristic allele constitution for each of the mouse-specific 31 markers (Table 3).

## 4. Discussion

Cell authentication is of the utmost importance to ensure valid and reproducible results when using a continuous cell line. However, cell line cross-contamination and accidental inoculation of a cell line with another line is a serious confounder in cell culture that can invalidate research results and undermine the comparison of results between laboratories [35]. Nevertheless, there is increasing evidence that globally a large number of cell lines have become contaminated, either as a result of erroneous cell culture handling or cross-contamination by other cell lines [36]. This problem has been known for over 60 years, but the authenticity of cell lines used in biomedical research has received little attention [37]. The frequency of cell line misidentification is rather high. A conservative estimate data established with misidentified cell lines presented by Horbach and Halffman in 2017 estimated that 32,755 research articles were conducted with misidentified cells that were in turn cited by about half a million other papers [38]. According to another estimate that is based on submission to major cell repositories, between 18% and 36% of all cell lines used are contaminated or misidentified [39]. Consequently, several funding agencies and publishers have established very thorough and helpful guidelines and documentary standards for authors when working with continuous cell lines [5]. However, morphological evaluation, cell-specific protein and/or expression patterns, as well as simple functional evaluations, are often the basis for leading a scientist to believe that he/she is working with the correct cell line. However, as discussed above, this can have serious consequences.

Nowadays, several principles and techniques were proposed in the frame of Good Cell Culture Practice (GCCP) [40]. In particular, karyotyping and species-specific short tandem repeat (STR) profiling have been proposed for the routine identification of a cell line and to reduce the frequency of cell misidentification [41]. However, the implementation of these guidelines for non-human cell lines is an ongoing challenge [42].

There are ~30 cell lines available that are purportedly of hepatic stellate cell origin. They were established from mouse, rat, and human. However, for only two of them are STR profiles available, namely the murine cell line GRX [8] and the human cell line LX-2 [7], while none of the rat lines was genetically characterized yet.

This is somewhat astonishing because some of these lines were used in high frequency in studies investigating aspects of hepatic stellate cell functionality and biology. The line HSC-T6 was established in year 1998 by transforming primary HSC from Sprague-Dawley rats with the large T-antigen from Simian virus 40 [2,3]. It is the most widespread rat HSC line and there are about 600 studies available reporting findings that were based on this line (Figure 1, Table S1). In particular, the cell line is most common in laboratories located in China, Taiwan, Korea, and the US. HSC-T6 cells display similar features as the activated primary HSC, including a fibroblastic morphology resembling that of myofibroblasts (Figure 2), and similar ultrastructural features (Figure 3), expression profiles (Figure 4), and microfilament bundles and networks (Figure 5A), as well as the capacity to take up and store fatty acids (Figure 5B). Moreover, previous studies have demonstrated that these cells express all six retinoid nuclear receptors (RARα, RARβ, RARγ, RXRα, RXRβ, and RXRγ), the cellular retinol-binding protein type I (CRBP1), the lecithin:retinol acyltransferase (LRAT) and acyl-Coa:retinol acyl transferase (ARAT) [3]. This establishes the HSC-T6 line as a valuable cell model for studies of retinoid metabolism. Nevertheless, although this cell line is commercially available for biomedical research and analysis, it had not previously been genetically characterized.

In the study presented here, we have established for the first time a meaningful STR profile (Table 3), which allows rapid and reliable cell authentication of this continuous line.

Aside from the genome-wide STR profiling, our study identifies gross chromosomal rearrangements (Figure 6 and Figure 7, Table 2). In particular, the complete gain of two chromosomes (RNO4 and RNO7) along with the characteristic derivative RNO1 can be considered as a constitutive chromosomal feature of HSC-T6. Interestingly, an extra copy of RNO4 is also reported in virally transformed glial cells [43] and rat endometrial carcinoma [44]. Furthermore, it is noteworthy that all three chromosomes (RNO4, RNO7, and RNO12) possess three distinct oncogenes (*k-ras*, *c-myc,* and *brca2*). It can therefore be inferred that the excess of two oncogenes (*k-ras* and *c-myc*) and the translocation of RNO12 containing *brca2* to RNO1 and RNO4 may have a role in contributing to the unrestrained proliferation of HSC-TC6 cells. However, we have no experimental data yet to support this assumption and more specialized studies will be necessary. Apart from the major rearrangement, the SKY analysis is able to detect random aberrations, each confined to two cells. These aberrant cells may represent either ongoing background instability or potentially these can give rise to novel clones. We anticipate that the chromosomal features for HSC-T6 presented here are of potential interest for laboratories utilizing multiple non-human HSC lines.

In this study, we further performed mRNA bulk NGS of RNA isolated from HSC-T6 that were grown under basal conditions. This analysis has revealed that the cell line has a large repertoire of different mRNAs (Table S3). In particular, there is high expression of characteristic HSC markers. These include, for example, *Acta2*, *Col1a1*, *Col3a1*, *Csrp2*, *Vim*, *Fn1*, and many others. Most of these markers are reliable markers indicating the cellular activation of HSC [45]. Moreover, the expression of tetherin by HSC-T6 cells might reflect a potentially broad role in antiviral response. We have recently identified tetherin expression in the murine HSC line, GRX, which may allow the retention of nascent retroviral particles at the surface by linking them to the cellular membrane [8]. Although not systematically tested yet, it is possible that HSC have a general antiviral activity limiting viral pathogenesis. Somewhat surprising is the finding that HSC-T6 express extremely high levels of the ferritin-heavy chain 1 (*Fth1*). This protein has a ferroxidase activity, is involved in intracellular iron storage, and protects from ferroptosis [46]. In this regard, a previous report performed in smooth muscle cells demonstrated that increased ferritin heavy chain expression correlates with an enhanced differentiated phenotype [47]. Thus, it is possible that the ferritin heavy chain is necessary to maintain the activated/transdifferentiated phenotype of the cells. Although we have not validated the NGS by independent RT-qPCR analysis, many genes in our analysis were previously detected in HSC or immortalized HSC lines [1,45]. In addition, the expression by HSC-T6 cell of a large repertoire of retinoid nuclear receptors confirms the previous suggestion that HSC-T6 display the same retinoid-related phenotype as primary HSC, and are therefore a useful experimental tool to study retinoid biology in this cell type [3].

Many previous research results were established using misidentified or contaminated cell lines [38], which lead to false interpretations, predictions, correlations, conclusions, and misguided follow-up studies [5,35,36,38]. The established genetic characteristics of HSC-T6 will now allow easy cell authentication and increase the general validity of this line in biomedical research.

The detailed characterization of different continuous cell lines is of fundamental importance. As our study demonstrates, distinct HSC cell lines can display different HSC markers or altered sensitivity toward pro-fibrogenic markers. Still, no HSC cell line can mimic all features of primary HSCs. Nevertheless, validating key experiments using more than one cell line can minimize the interpretation of results that are unique to a single line. The cellular and molecular characteristics might significantly differ and can be variable in their biological properties, and each line has its advantages and disadvantages. Therefore, the choice of cell line to use needs to account for these issues. In particular, the immortalization with SV40T that is a potent transforming protein might significantly modulate not only cellular proliferation, gene expression, metabolism, but also could disrupt genome integrity and induce DNA damage response signaling [48]. Similarly, the murine HSC line GRX cultures are permanently infected with retroviruses, which could also affect the general characteristics of this cell line; working with this cell line might be a source of infection for other cell lines [8]. In some countries, continuous cell lines established from humans (e.g., LX-2) might require the approval of institutional ethical committees.

## 5. Conclusions

HSC-T6 cells are widely used as an important experimental tool for studies of hepatic metabolism. The identified chromosomal arrangement and the definition of a unique STR profile for HSC-T6 cells now allows investigators to verify their identity and prevent the erroneous interpretations of data. This is an important step for fulfilling the recommendations of granting agencies and scientific journals when working with continuous growing cell lines. Western blot expression analysis and mRNA bulk NGS sequencing further revealed that HSC-T6 expresses typical HSC markers including collagen type I, α-SMA, Vimentin, and Fibronectin, confirming that this cell line originated from HSC. In line, HSC-T6 cells contain robust microfilaments and are capable of storing large lipid fat droplets in their cytoplasm. In addition, this cell line still produces oncogenic SV40T, which can be used as a characteristic feature of these cells. In summary, the findings of our study will increase the biomedical value of HSC-T6 for liver research and provide an important benchmark for proper genetic characterization of other cell lines used in studies of liver biology, and in particular, hepatic fibrogenesis.

## Figures and Tables

**Table 1:** Gene expression in HSC-T6 cells under basal culture conditions as determined by next generation mRNA sequencing.

Transcript Id	Gene Id	Gene	Gene Description	TPM	Remarks
ENSRNOT00000083468.1	ENSRNOG00000058039.1	*Acta2*	Actin alpha 2, smooth muscle	2.377	HSC marker
ENSRNOT00000005311.6	ENSRNOG00000003897.6	*Col1a1*	Collagen type I alpha 1 chain	2033.74	HSC marker
ENSRNOT00000004956.4	ENSRNOG00000003357.4	*Col3a1*	Collagen type III alpha 1 chain	2128.9	HSC marker
ENSRNOT00000024430.5	ENSRNOG00000018087.5	*Vim*	Vimentin	2054.81	HSC marker
ENSRNOT00000057585.4	ENSRNOG00000014288.8	*Fn1*	Fibronectin 1	143.953	HSC marker
ENSRNOT00000019772.6				352.585	
ENSRNOT00000013538.6	ENSRNOG00000009884.6	*Lgals1*	Galectin 1	4801.86	HSC marker
ENSRNOT00000017486.7	ENSRNOG00000012840.7	*Sparc*	Secreted protein acidic and cysteine rich	2188.34	HSC marker
ENSRNOT00000067011.2	ENSRNOG00000003772.7	*Csrp2*	Cysteine and glycine-rich protein 2	195.244	HSC marker
ENSRNOT00000013745.7	ENSRNOG00000010208.7	*Timp1*	TIMP metallopeptidase inhibitor	5518.57	Inhibitor of MMPs
ENSRNOT00000004290.4	ENSRNOG00000003148.5	*Timp2*	TIMP metallopeptidase inhibitor 2	369.667	Inhibitor of MMPs
ENSRNOT00000010180.5	ENSRNOG00000007650.5	*Cd63*	Cd63 molecule	1639.48	TIMP1 receptor
ENSRNOT00000011208.7	ENSRNOG00000008301.7	*Tagln2*	Transgelin 2	754.93	Smooth muscle marker
ENSRNOT00000015962.6	ENSRNOG00000020660.7	*Cfl1*	Cofilin 1	1670.37	Cytoskeleton
ENSRNOT00000042459.4	ENSRNOG00000034254.4	*Actb*	Actin, beta	4379.5	Cytoskeleton
ENSRNOT00000080216.1				1820.7	
ENSRNOT00000078250.1	ENSRNOG00000056836.1	*Cav1*	Caveolin 1	167.988	Scaffolding protein
ENSRNOT00000008659.4	ENSRNOG00000009972.7	*Rara*	Retinoic acid receptor, alpha	24.8697	Nuclear receptors
ENSRNOT00000084644.1				23.8544	
ENSRNOT00000033048.6	ENSRNOG00000024061.7	*Rarb*	Retinoic acid receptor, beta	0.541197	Nuclear receptors
ENSRNOT00000016801.5	ENSRNOG00000012499.7	*Rarg*	Retinoic acid receptor, gamma	64.5919	Nuclear receptors
ENSRNOT00000017096.7				39.1824	
ENSRNOT00000012892.4	ENSRNOG00000009446.4	*Rxra*	Retinoid X receptor alpha	18.9052	Nuclear receptors
ENSRNOT00000041613.5	ENSRNOG00000000464.7	*Rxrb*	Retinoid X receptor beta	20.0384	Nuclear receptors
ENSRNOT00000087670.1				1.79029	
ENSRNOT00000086978.1				1.08069	
ENSRNOT00000081588.1				0.804912	
ENSRNOT00000091182.1				0.723898	
ENSRNOT00000087895.1				0.688452	
ENSRNOT00000084638.1				0.499241	
ENSRNOT00000019571.3	ENSRNOG00000014090.3	*Retsat*	Retinol saturase	52.476	Retinoid metabolism
ENSRNOT00000081756.1	ENSRNOG00000053850.1	*Rdh5*	Retinol dehydrogenase 5	0.0383589	Retinoid metabolism
ENSRNOT00000032076.4	ENSRNOG00000025767.4	*Rdh8*	Retinol dehydrogenase 8	0.112568	Retinoid metabolism
ENSRNOT00000009096.4	ENSRNOG00000006681.4	*Rdh10*	Retinol dehydrogenase 10	3.87175	Retinoid metabolism
ENSRNOT00000085927.1	ENSRNOG00000054770.1	*Rdh11*	Retinol dehydrogenase 11	26.8725	Retinoid metabolism
ENSRNOT00000078436.1				0.15904	
ENSRNOT00000089162.1	ENSRNOG00000056553.1	*Rdh12*	Retinol dehydrogenase 12	1.06243	Retinoid metabolism
ENSRNOT00000031462.5	ENSRNOG00000027919.5	*Rdh13*	Retinol dehydrogenase 13	1.96789	Retinoid metabolism
ENSRNOT00000006020.6	ENSRNOG00000039551.3	*Rdh14*	Retinol dehydrogenase 14	8.16116	Retinoid metabolism
ENSRNOT00000093003.1				2.97058	
ENSRNOT00000092948.1				0.857514	
ENSRNOT00000018622.4	ENSRNOG00000013794.4	*Rbp1*	Retinol binding protein 1	25.3842	Retinoid metabolism
ENSRNOT00000018755.6	ENSRNOG00000013932.6	*Rbp2*	Retinol binding protein 2	0.309974	Retinoid metabolism
ENSRNOT00000021055.7	ENSRNOG00000015518.7	*Rbp4*	Retinol binding protein 4	0.0363529	Retinoid metabolism
ENSRNOT00000021348.5	ENSRNOG00000015850.5	*Rbp7*	Retinol binding protein 7	0.244971	Retinoid metabolism
ENSRNOT00000082156.1	ENSRNOG00000025608.4	*Lrat*	lecithin retinol acyltransferase	0.0125274	Retinoid metabolism
ENSRNOT00000022113.4	ENSRNOG00000016275.4	*Ttr*	Transthyretin	0.161195	RBP transporter
ENSRNOT00000030919.5	ENSRNOG00000022619.6	*Fth1*	Ferritin heavy chain 1	9890.55	Iron storage
ENSRNOT00000087162.1	ENSRNOG00000059900.1	*Bst2*	Bone marrow stromal cell antigen 2	1681.66	Antiviral response
ENSRNOT00000091906.1				589.283	
ENSRNOT00000080988.1	ENSRNOG00000052802.1	*Aldoa*	Aldolase, fructose-bisphophate	1471.21	Glycolysis
ENSRNOT00000087928.1				492.926	
ENSRNOT00000088473.1				183.94	
ENSRNOT00000015332.7	ENSRNOG00000011329.7	*Pkm*	Pyruvate kinase M1/2	1437.52	Glycolysis
ENSRNOT00000083666.1				455,466	
ENSRNOT00000077604.1	ENSRNOG00000058249.1	*Pgk1*	Phosphoglycerate kinase 1	1404.31	Glycolysis
ENSRNOT00000085653.1				0.204793	
ENSRNOT00000050443.4	ENSRNOG00000018630.7	*LOC108351137*	Glyceraldehyde-3-phosphate dehydrogenase	4598.86	Glycolysis
ENSRNOT00000041328.3	ENSRNOG00000030963.3			4243.2	

Abbreviations used: MMPs, matrix metalloproteinases; RBP, retinol-binding protein.

**Table 2:** Common and sporadic structural chromosomal aberrations in HSC-T6.

Cell	Chromosome Numbers	der(1)t(1;4;12)	der(1)t(1;4)	der(1)t(1;15)	der(3)t(3;18)	der(4)t(4;12)	der(7)t(7;18)	der(7)t(7;19)	der(7) t(7;20)	der(10)t(10;12;13)	der(12)t(12:18)	der(13)t(13;17)	der(15)t(15;17)	der(15)t(15;18)	der(X)t(X;20)
1	41,XX	●				●		●	●						
2	40,XX	●	●	●				●				●	●	●	
3	44,XX	●				●		●							
4	41,XX	●				●		●							
5	41,XX	●				●		●							
6	40,XX	●				●		●							
7	43,XX	●				●		●							
8	43,XX	●				●		●		●					
9	42,XX	●				●	●	●							●
10	43,XX	●				●		●						●	
11	43,XX	●				●	●	●							
12	43,XX	●				●		●							
13	42,XX	●				●		●							
14	42,XX	●			●	●		●							
15	43,XX		●			●		●			●				
16	43,XX	●				●		●							
17	43,XX	●				●		●							
18	43,XX	●			●	●		●							
19	41,XX		●			●		●			●				
20	43,XX	●				●		●							

**Table 3:** STR-based DNA profiling of HSC-T6 cells using the 31 species-specific STR markers.

SN	Marker Name ^1^	Chromosomal Location	Allele Sizes (bp)
1	73	1	194
2	8	2	234
3	2	2	127
4	4	3	238
5	3	3	160, 162
6	26	4	166
7	19	4	175
8	81	5	130, 132
9	34	6	188
10	30	7	186, 192
11	24	8	249, 253
12	59	9	143, 146, 180 ^2^
13	62	9	177
14	1	10	96
15	55	10	210, 218
16	36	11	234
17	67	11	165
18	13	12	121, 135
19	35	13	197, 203
20	42	13	127
21	70	14	175, 179
22	61	15	128
23	79	15	172
24	90	16	174
25	69	16	139
26	78	17	147, 151
27	15	18	232
28	16	18	247, 251
29	75	19	144, 184
30	96	20	210, 212
31	91	20	205, 211

^1^ Profiling was conducted using the CellCheck^TM^ Rat Panel (IDEXX BioAnalytics, Columbia, MO, USA); ^2^ based on the electropherogram, a tri-allelic variant is assumed at this STR marker site. Abbreviations used: bp, base pairs; SN, serial number; STR, short tandem repeat. The individual electropherograms are depicted in Figure S7.

## Data Availability

This manuscript contains most of the original data generated during our study. However, additional datasets (e.g., the complete fastq data file of our NGS analysis, additional illustrations of the SKY painting) can be requested from the corresponding author.

## Supplementary Materials

The following supporting information can be downloaded at: https://www.mdpi.com/article/10.3390/cells11111783/s1, Table S1: Publications using or mentioning HSC-T6 cells, Table S2: Primers used in this study, Table S3: Gene expression in HSC-T6 cells under basal culture conditions as determined by next generation mRNA sequencing, Figure S1: Testing HSC-T6 cells for potential mycoplasma contaminations, Figure S2: Expression of GFAP in HSC-T6 cells, Figure S3: Marker gene expression in HSC-T6 and stimulation experiments, Figure S4: Short term stimulation experiments, Figure S5: Long term stimulation experiments, and Figure S6: Expression of Ferritin heavy chain 1 (Fth1) in HSC-T6 cells, Figure S7: HSC-T6 short tandem repeat (STR) markers.
