# Supplementary material for: Genetic Characterization of Rat Hepatic Stellate Cell Line HSC-T6 for In Vitro Cell Line Authentication
Source: Cells. 2022 May 29;11(11):1783. doi: 10.3390/cells11111783 (PMC9179542; doi:10.3390/cells11111783)
Supplement: Supplementary file 1 [file cells-11-01783-s001.zip › Figure S1 to Figure S6.pdf]

Supplementary Figures

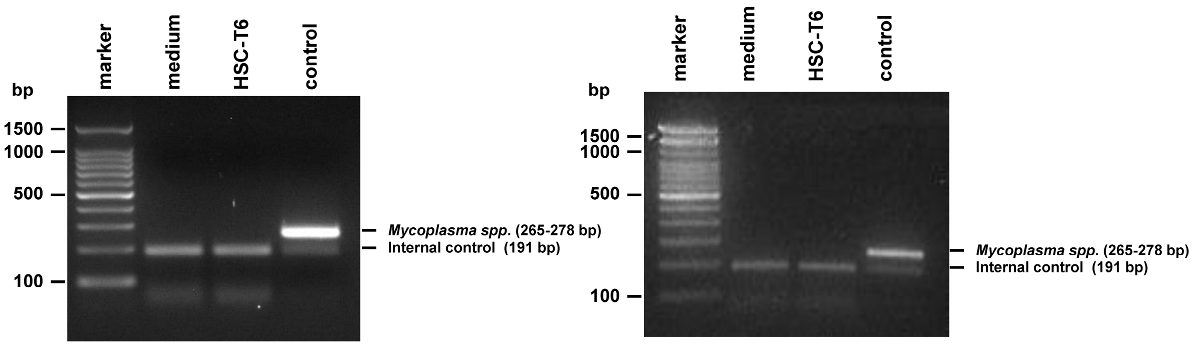

Figure S1. Testing HSC-T6 cells for potential mycoplasma contaminations. Fresh medium (medium) and cell supernatant from a semi-confluent (left panel) and a confluent (right panel) HSC-T6 culture (HSC-T6) were tested for potential mycoplasma contaminations using a commercially available mycoplasma detection kit for conventional PCR using the manufacturer's instructions. According to the suppliers' information provided, the primers of the kit system detect the 16S rRNAs of 1 *Ureaplasma*, 7 *Acholeplasma* and 85 *Mycoplasma* species. Depending on the mycoplasma species, the amplicon is in the range of 265-278 bp size range. In addition, the kit amplifies an internal control DNA of 191 bp in size in each sample to verify whether the PCR reaction took place without any inhibition. A positive control (control) from the kit demonstrates the full functionality of the detection assay. The fragments were separated in a 2% agarose gel, which contained ethidium bromide.

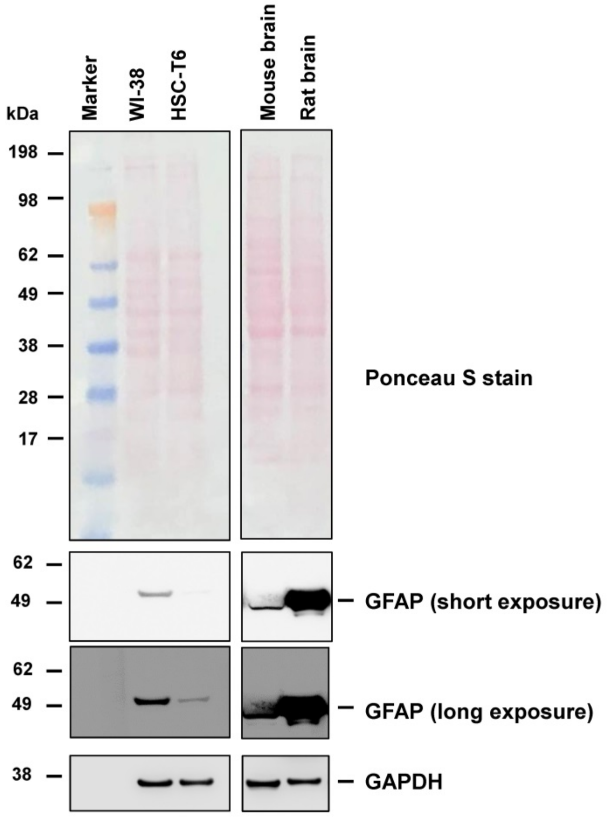

Figure S2. Expression of GFAP in HSC-T6 cells. Protein extracts from WI-38 and HSC-T6 cells (40 µg per lane) were analyzed for expression of GFAP. Extracts from mouse and rat brain tissues (60 µg per lane) were taken as control. Shown are short and long exposures. GAPDH expression and Ponceau S stain severed as controls.

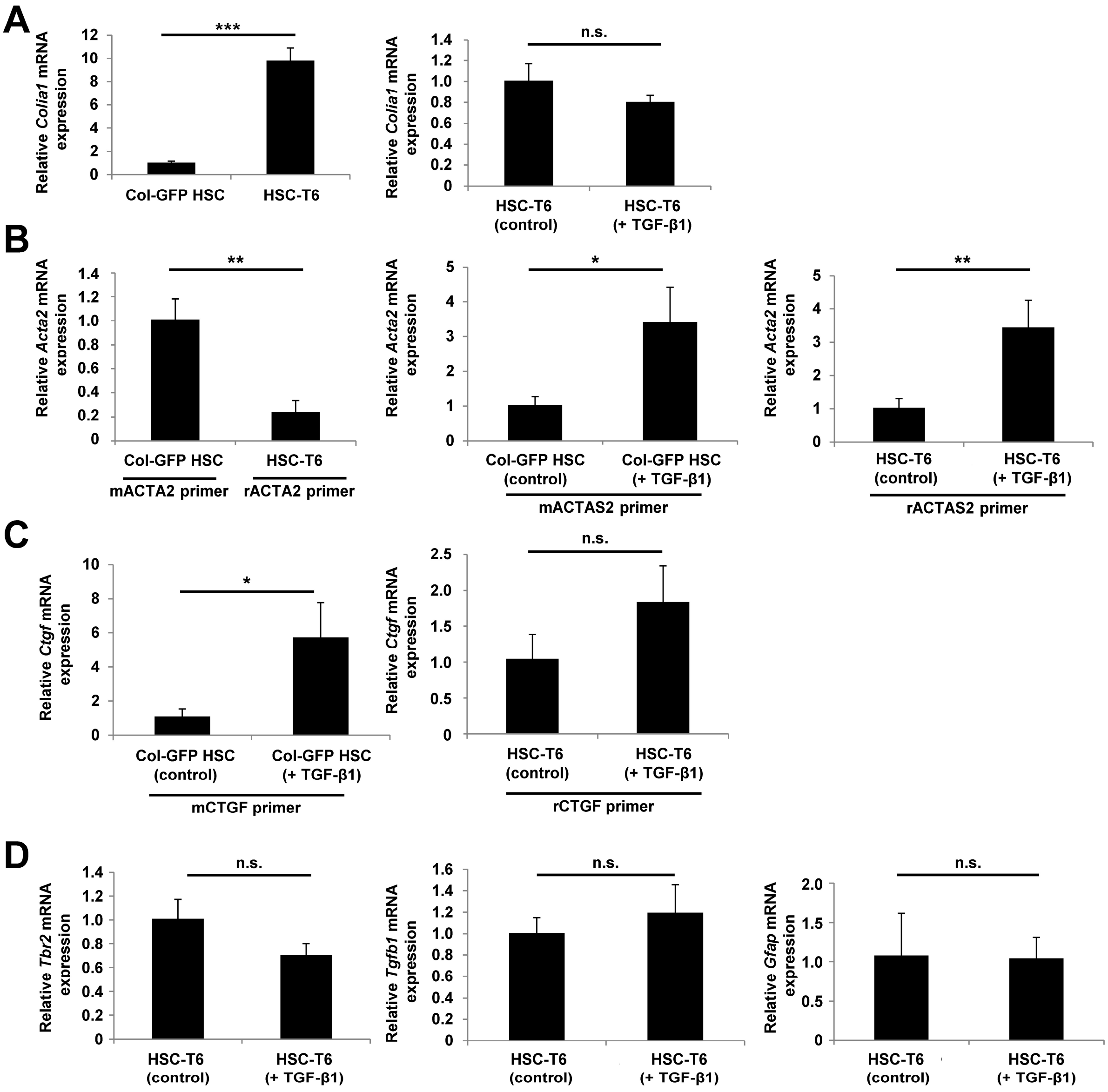

Figure S3. Marker gene expression in HSC-T6 and stimulation experiments. (**A**) Col-GFP HSC and HSC-T6 cells cultured under basal conditions were analyzed for expression of collagen type I α1 (*CollaI*) mRNA (*left panel*). Please note that compared to Col-GFP HSC, the cell line HSC-T6 contained significant higher quantities of *colla1* mRNA. Stimulation of HSC-T6 with TGF-β1 does not induce *Colla1* mRNA expression (*right panel*), possibly because expression is already extremely high. (**B**) Compared to Col-GFP, the cell line HSC-T6 showed higher expression of α-smooth muscle actin (*Acta2*) under basal condition (*left panel*). Similar to Col-GFP HSC cells (*middle panel*), the expression of *Acta2* can be significantly stimulated by TGF-β1 (*right panel*). (**C**) Connective tissue growth factor (*Ctgf*) mRNA expression is significantly induced by TGF-β1 (*left panel*), while there is only a tendency to induce this gene in HSC-T6 cells (*right panel*). (**D**) Moreover, TGF-β1 does not induce transforming growth factor receptor type II (*Tbr2*) (*left panel*), TGF-β1 (*Tgfb1*) (*middle panel*), or *Gfap* in HSC-T6 cells (*right panel*).

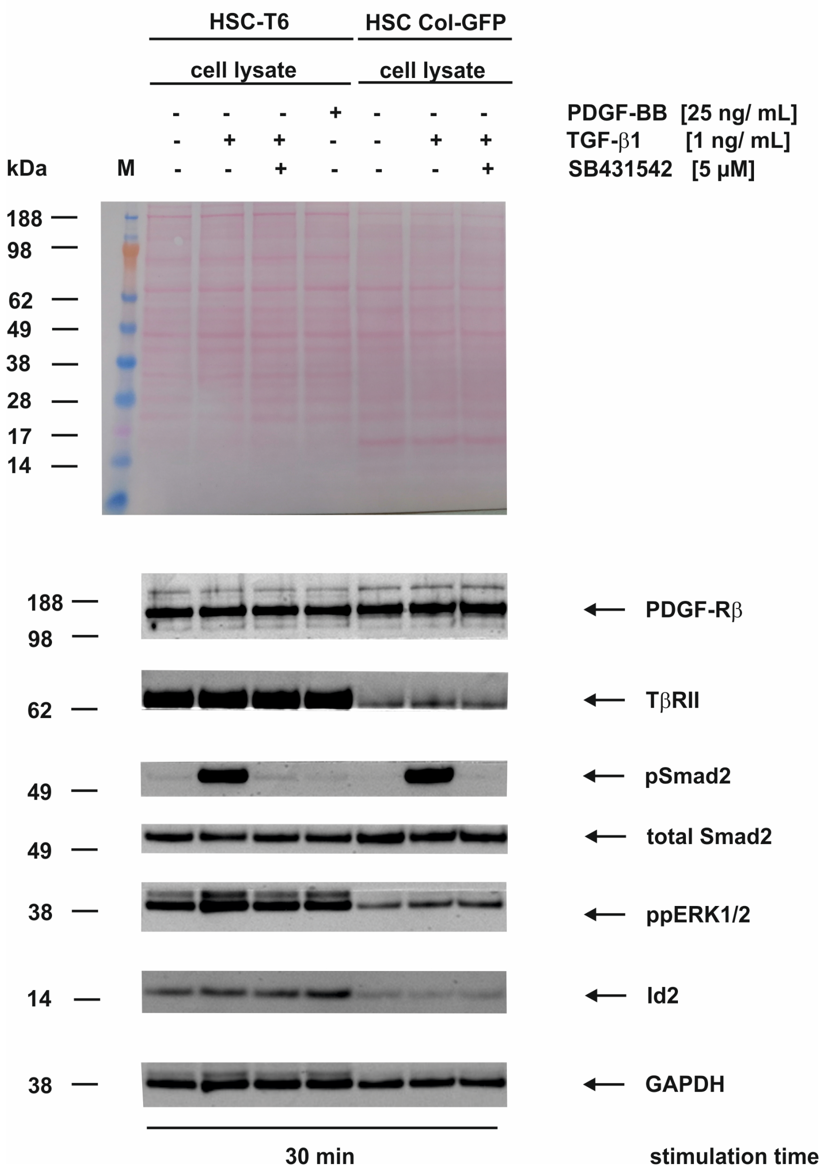

Figure S4. Short term stimulation experiments. HSC-T6 or Col-GFP HSC cells were stimulated for 30 minutes with platelet-derived growth factor-B (PDGF-BB), TGF-β1 (in the presence or absence of ALK5 inhibitor SB431542), or left untreated. Cell extracts were prepared and analyzed by Western blot for indicated proteins. Please note that both cell lines increase phosphorylation of Smad2 after stimulation with TGF-β1.

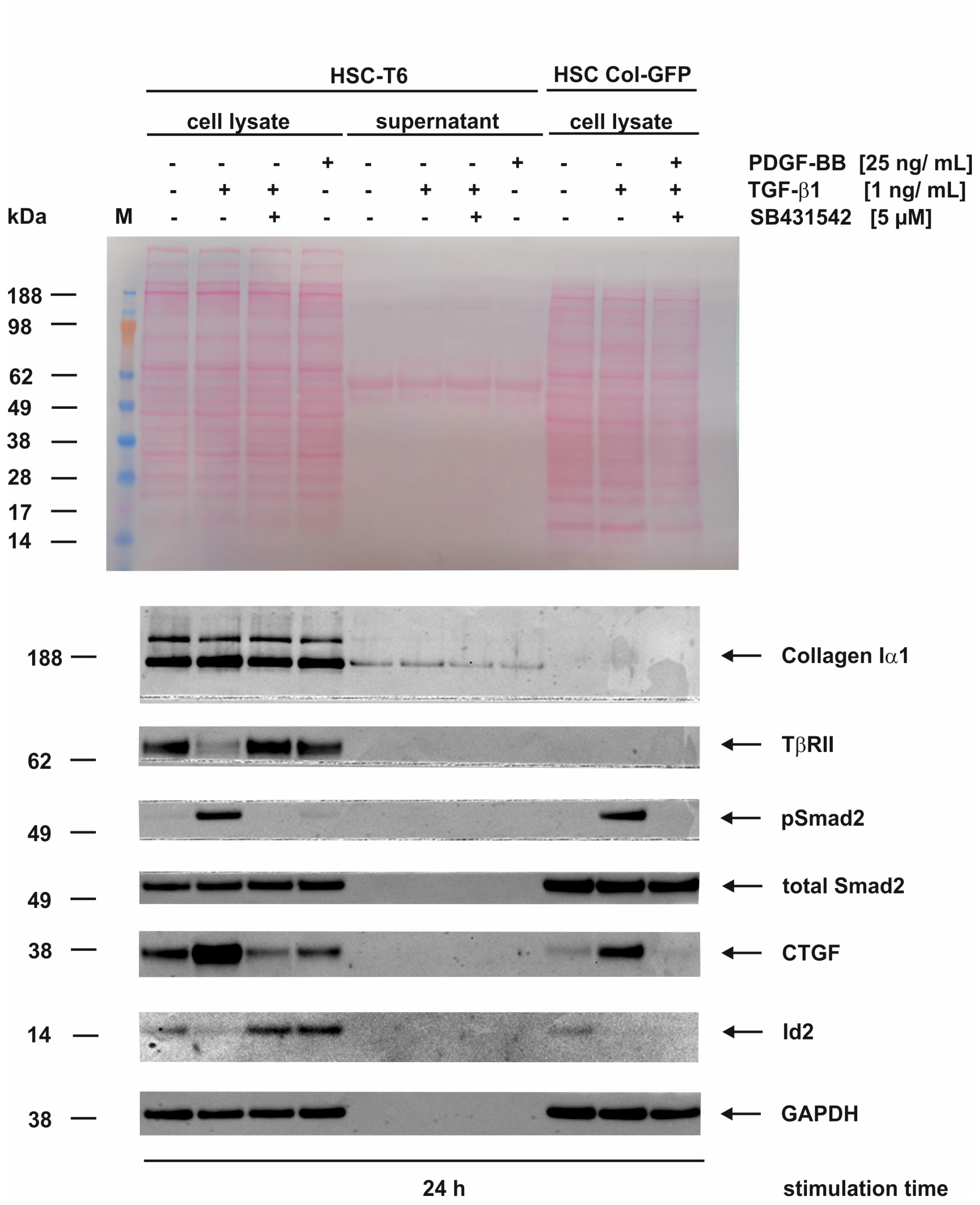

Figure S5. Long term stimulation experiments. HSC-T6 or Col-GFP HSC cells were stimulated for 24 minutes with platelet-derived growth factor-B (PDGF-BB), TGF-β1 (in the presence or absence of ALK5 inhibitor SB431542), or left untreated. Cell extracts (HSC-T6, Col-GFP HSC) and supernatants (HSC-T6) were analyzed by Western blot for indicated proteins. Please note that both cell lines showed increased phosphorylation of Smad2 and CTGF expression after stimulation with TGF-β1. The lower quantities of transforming growth factor-β receptor type II after TGF-β1 stimulation is due to receptor internalization. Please note, that the expression of collagen type I does not increase after stimulation.

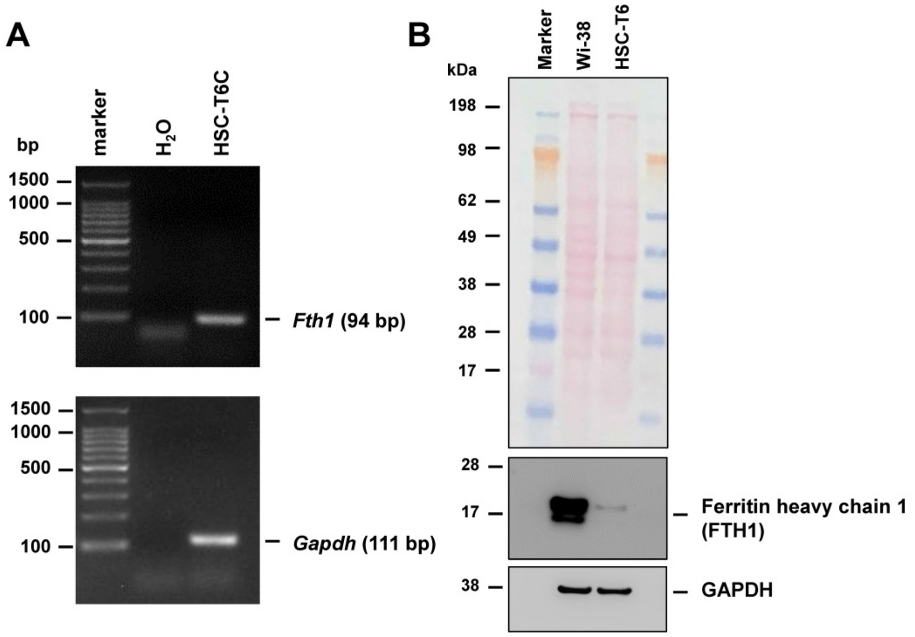

Figure S6. Expression of Ferritin heavy chain 1 (Fth1) in HSC-T6 cells. (**A**) Expression of *Fth1* was tested by RT-PCR. A no template control (H_2O) and expression of Glyceraldehyde 3-phosphate dehydrogenase (*Gapdh*) served as controls. (**B**) Protein extracts from WI-38 and HSC-T6 were analyzed by Western blot for expression of FTH1. The blot was re-probed with an antibody specific for GAPDH. Ponceau S stain served as a control to demonstrate equal protein loading in each lane.

Figure S7. HSC-T6 short tandem repeat (STR) markers. Genomic DNA from HSC-T6 cells was isolated and a short tandem repeat (STR) profile determined using 31 rat specific markers. Shown are the electropherograms determined for each marker. For this figure refer to separate pdf-file (Figure S7).
