# Supplementary material for: Genetic Characterization of Rat Hepatic Stellate Cell Line HSC-T6 for In Vitro Cell Line Authentication
Source: Cells. 2022 May 29;11(11):1783. doi: 10.3390/cells11111783 (PMC9179542; doi:10.3390/cells11111783)
Supplement: Supplementary file 1 [file cells-11-01783-s001.zip › Table S1.pdf]

Table S1. Publications using or mentioning HSC-T6 cells

S/N	Year of publication	Reference	Origin of publication
1	1998	Kim Y, Ratziu V, Choi SG, Lalazar A, Theiss G, Dang Q, Kim SJ, Friedman SL. Transcriptional activation of transforming growth factor beta1 and its receptors by the Kruppel-like factor Zf9/core promoter-binding protein and Sp1. Potential mechanisms for autocrine fibrogenesis in response to injury. J Biol Chem. 1998;273(50):33750-8. doi: 10.1074/jbc.273.50.33750	USA / Germany
2	1999	Nieto N, Friedman SL, Greenwel P, Cederbaum AI. CYP2E1-mediated oxidative stress induces collagen type I expression in rat hepatic stellate cells. Hepatology 1999;30(4):987-96. doi: 10.1002/hep.510300433	USA
3	2000	Ankoma-Sey V, Wang Y, Dai Z. Hypoxic stimulation of vascular endothelial growth factor expression in activated rat hepatic stellate cells. Hepatology 2000;31(1):141-8. doi: 10.1002/hep.510310122	USA
4	2000	Zhang M, Zhang JP, Ji HT, Wang JS, Qian DH. Effect of six flavonoids on proliferation of hepatic stellate cells in vitro. Acta Pharmacol Sin. 2000;21(3):253-6. PMID: 11324426	China
5	2000	Vogel S, Piantedosi R, Frank J, Lalazar A, Rockey DC, Friedman SL, Blaner WS. An immortalized rat liver stellate cell line (HSC-T6): a new cell model for the study of retinoid metabolism in vitro. J Lipid Res. 2000;41(6):882-93. PMID: 10828080	USA
6	2000	Paik J, Vogel S, Piantedosi R, Sykes A, Blaner WS, Swisshelm K. 9-cis-retinoids: biosynthesis of 9-cis-retinoic acid. Biochemistry 2000;39(27):8073-84. doi: 10.1021/bi992152g	USA
7	2001	Bruck R, Genina O, Aeed H, Alexiev R, Nagler A, Avni Y, Pines M. Halofuginone to prevent and treat thioacetamide-induced liver fibrosis in rats. Hepatology 2001;33(2):379-86. doi: 10.1053/jhep.2001.21408	Israel
8	2001	Zhang JP, Zhang M, Zhou JP, Liu FT, Zhou B, Xie WF, Guo C. Antifibrotic effects of matrine on in vitro and in vivo models of liver fibrosis in rats. Acta Pharmacol Sin. 2001;22(2):183-6. PMID: 11741525	Israel
9	2001	Zhang JP, Zhang M, Jin C, Zhou B, Xie WF, Guo C, Zhang C, Qian DH. Matrine inhibits production and actions of fibrogenic cytokines released by mouse peritoneal macrophages. Acta Pharmacol Sin. 2001;22(8):765-8. PMID: 11749854	China
10	2001	Kang LP, Qi LH, Zhang JP, Shi N, Zhang M, Wu TM, Chen J. Effect of genistein and quercetin on proliferation, collagen synthesis, and type I procollagen mRNA levels of rat hepatic stellate cells. Acta Pharmacol Sin. 2001;22(9):793-6. PMID: 11749858	China
11	2001	Qi LH, Kang LP, Zhang JP, Shi N, Zhang M, Wu TM. [Antifibrotic effects of genistein and quercetin in vitro]. Yao Xue Xue Bao 2001;36(9):648-51. Chinese. PMID: 12580099	China

12	2002	Saxena NK, Ikeda K, Rockey DC, Friedman SL, Anania FA. Leptin in hepatic fibrosis: evidence for increased collagen production in stellate cells and lean littermates of ob/ob mice. Hepatology 2002;35(4):762-71. doi: 10.1053/jhep.2002.32029	USA
13	2002	Ding HG, Wang BE, Shang HW. [Effect of herbal compound 861 on expression and activity of nitric oxide synthase in hepatic stellate cells]. Zhongguo Zhong Xi Yi Jie He Za Zhi. 2002;22(5):362-4. Chinese. PMID: 12584838	China
14	2002	Yin C, Ma H, Wang A, Ma X, Jia J, Wang B. [Effect of compound 861 on tissue inhibitor of metalloprotenase 1 gene expression of HSC-T6 cells]. Zhonghua Gan Zang Bing Za Zhi. 2002;10(3):197-9. Chinese. PMID: 12113678	China
15	2002	During A, Hussain MM, Morel DW, Harrison EH. Carotenoid uptake and secretion by CaCo-2 cells: beta-carotene isomer selectivity and carotenoid interactions. J Lipid Res. 2002;43(7):1086-95. doi: 10.1194/jlr.m200068-jlr200	USA
16	2003	Chen YX, Lu CH, Xie WF, Zhang XR, Zhang ZB, Wei LX, Jin YX, Guo YJ. [Effect of ribozyme against platelet-derived growth factor receptor beta subunit mRNA on the biological characters of hepatic stellate cells]. Zhonghua Gan Zang Bing Za Zhi 2003;11(5):278-81. Chinese. PMID: 12773241	China
17	2004	Zhang G, Zhang FC, Wang TC, Liang KH. [The effects of Chinese national medicine of Huoxueruanjian compound on SMAD signal in hepatic stellate cell and its significance]. Zhonghua Gan Zang Bing Za Zhi 2004;12(4):213-5. Chinese. PMID: 15099469	China
18	2004	Dong MS, Jung SH, Kim HJ, Kim JR, Zhao LX, Lee ES, Lee EJ, Yi JB, Lee N, Cho YB, Kwak WJ, Park YI. Structure-related cytotoxicity and anti-hepatofibric effect of asiatic acid derivatives in rat hepatic stellate cell-line, HSC-T6. Arch Pharm Res. 2004;27(5):512-7. doi: 10.1007/BF02980124	Korea
19	2004	Ding HG, Wang BE, Jia JD, Xia HX, Wong CY, Zhao CH, Xu YL. Effects of octreotide on expression of L-type voltage-operated calcium channels and on intracellular Ca2+ in activated hepatic stellate cells. Chin Med J. (Engl) 2004;117(6):913-6. PMID: 15198898	China
20	2004	Abu-Absi SF, Hansen LK, Hu WS. Three-dimensional co-culture of hepatocytes and stellate cells. Cytotechnology 2004;45(3):125-40. doi: 10.1007/s10616-004-7996-6	USA
21	2004	Huang J, Hu JH, Qiu L, Cai Z. [Mechanisms of sodium ferulate inhibition of collagen synthesis in hepatic stellate cells]. Yao Xue Xue Bao 2004;39(8):577-80. Chinese. PMID: 15563055	China
22	2004	Li GM, Shi Y, Li DG, Xie Q, Guo Q, Jin YX. [Effect of small interfering RNA targeting connective tissue growth factor on the synthesis and secretion of extracellular matrix in hepatic stellate cells]. Zhonghua Gan Zang Bing Za Zhi 2004;12(9):526-9. Chinese. PMID: 15387902	China
23	2004	Wang X, Tang X, Gong X, Albanis E, Friedman SL, Mao Z. Regulation of hepatic stellate cell activation and growth by	USA

		transcription factor myocyte enhancer factor 2. Gastroenterology 2004;127(4):1174-88. doi: 10.1053/j.gastro.2004.07.007	
24	2004	Fiorucci S, Antonelli E, Rizzo G, Renga B, Mencarelli A, Riccardi L, Orlandi S, Pellicciari R, Morelli A. The nuclear receptor SHP mediates inhibition of hepatic stellate cells by FXR and protects against liver fibrosis. Gastroenterology 2004;127(5):1497-512. doi: 10.1053/j.gastro.2004.08.001	Italy
25	2005	Badria FA, Dawidar AA, Houssen WE, Shier WT. In vitro study of flavonoids, fatty acids, and steroids on proliferation of rat hepatic stellate cells. Z Naturforsch C J Biosci. 2005;60(1-2):139-42. doi: 10.1515/znc-2005-1-225	Egypt / USA
26	2005	Hsu YC, Lin YL, Chiu YT, Shiao MS, Lee CY, Huang YT. Antifibrotic effects of Salvia miltiorrhiza on dimethylnitrosamine-intoxicated rats. J Biomed Sci. 2005;12(1):185-95. doi: 10.1007/s11373-004-8167-7	Taiwan
27	2005	Song YH, Zhou XM, Xue XN, Liu NZ, Tian DA, Kong XJ, Wu XL, Lin JS, Jin YX. Effect of ribozyme against transforming growth factorbeta1 on biological character of activated HSCs. IUBMB Life 2005;57(1):31-9. doi: 10.1080/15216540400024470	China
28	2005	Li X, Meng Y, Jiang B, Yang XS, Wang WW, Guo D, Lai ZS, Zhang ZS. [Effects of angiotensin II and aldosterone on NF-kappaB binding activity in hepatic stellate cells]. Zhonghua Yi Xue Za Zhi 2005;85(6):374-80. Chinese. PMID: 15854525	China
29	2005	Song YH, Chen XL, Kong XJ, Liu NZ, Li W, Wu XL, Lin JS, Jin YX. Ribozymes against TGFbeta1 reverse character of activated hepatic stellate cells in vitro and inhibit liver fibrosis in rats. J Gene Med. 2005;7(7):965-76. doi: 10.1002/jgm.744	China
30	2005	Ma H, Ma XM, Yin CH, Jia JD, Wang BE. [Effects of gamma-interferon on gene expression of collagen I, III and on the tissue inhibitor of metalloprotenase 1 in HSC-T6 cells]. Zhonghua Gan Zang Bing Za Zhi 2005;13(7):528-30. Chinese. PMID: 16042892	China
31	2005	Li X, Meng Y, Cai SX, Yang XS, Zhang YJ, Wu PS. [Angiotensin II and aldosterone stimulate alpha1-(I) procollagen mRNA expression in hepatic stellate cells via activation of ERK1/2 and AP-1]. Zhonghua Yi Xue Za Zhi 2005;85(26):1831-5. Chinese. PMID: 16253189	China
32	2005	Li X, Meng Y, Yang XS, Wu PS, Zhang ZS. [Aldosterone stimulating PDGF-B expression in HSC via activation of EGR-1]. Zhonghua Gan Zang Bing Za Zhi 2005;13(8):567-70. Chinese. PMID: 16092976	China
33	2005	Chor SY, Hui AY, To KF, Chan KK, Go YY, Chan HL, Leung WK, Sung JJ. Anti- proliferative and pro-apoptotic effects of herbal medicine on hepatic stellate cell. J Ethnopharmacol. 2005;100(1-2):180-6. doi: 10.1016/j.jep.2005.02.036	China
34	2005	Tan Y, Lu ZP, Ou SN, Zhang X. [Experimental study on effect of baoganning on activity of nuclear transcription factor-kappaB in hepatic stellate cells]. Zhongguo Zhong Xi Yi Jie He Za Zhi 2005;25(9):804-7. Chinese. PMID: 16248243	China
35	2005	Zhang Y, Liu Y. [Study on effects of salidroside on lipid peroxidation on oxidative stress in rat hepatic stellate cells]. Zhong Yao Cai 2005;28(9):794-6. Chinese. PMID: 16447872	China

36	2005	Traister A, Breitman I, Bar-Lev E, Zvibel I, Harel A, Halpern Z, Oren R. Nicotinamide induces apoptosis and reduces collagen I and pro-inflammatory cytokines expression in rat hepatic stellate cells. Scand J Gastroenterol. 2005;40(10):1226-34. doi: 10.1080/00365520510023341	Israel
37	2005	Zhang J, Zhang ZT, Wang Y, Wang P, Li JS, Zhou YZ. [Effect of endothelin-1 and its antagonists on the expression of endothelin receptors mRNA in HSC-T6 cells]. Zhonghua Wai Ke Za Zhi. 2005;43(21):1395-7. Chinese. PMID: 16318777	China
38	2005	Jiang Y, Li ZS, Jiang FS, Deng X, Yao CS, Nie G. Effects of different ingredients of zedoary on gene expression of HSC-T6 cells. World J Gastroenterol. 2005;11(43):6780-6. doi: 10.3748/wjg.v11.i43.6780	China
39	2006	Tan Y, Lv ZP, Bai XC, Liu XY, Zhang XF. Traditional Chinese medicine Bao Gan Ning increase phosphorylation of CREB in liver fibrosis in vivo and in vitro. J Ethnopharmacol. 2006;105(1-2):69-75. doi: 10.1016/j.jep.2005.09.040	China
40	2006	Liu X, Wang W, Hu H, Tang N, Zhang C, Liang W, Wang M. Smad3 specific inhibitor, naringenin, decreases the expression of extracellular matrix induced by TGF-beta1 in cultured rat hepatic stellate cells. Pharm Res. 2006;23(1):82-9. doi: 10.1007/s11095-005-9043-5	China
41	2006	Cheng K, Ye Z, Guntaka RV, Mahato RI. Enhanced hepatic uptake and bioactivity of type alpha1(I) collagen gene promoter-specific triplex-forming oligonucleotides after conjugation with cholesterol. J Pharmacol Exp Ther. 2006;317(2):797-805. doi: 10.1124/jpet.105.100347	USA
42	2006	Song YH, Xue XL, Zhao Q, Tian DA, Liu NZ, Huang HJ, Lin JS. [Hammerhead ribozyme-mediated cleavage of transforming growth factor beta1 RNA in a cell- free system and in hepatic stellate cells]. Zhonghua Gan Zang Bing Za Zhi 2006;14(2):93-6. Chinese. PMID: 16494775	China
43	2006	Chong LW, Hsu YC, Chiu YT, Yang KC, Huang YT. Anti-fibrotic effects of thalidomide on hepatic stellate cells and dimethylnitrosamine-intoxicated rats. J Biomed Sci. 2006;13(3):403-18. doi: 10.1007/s11373-006-9079-5	Taiwan
44	2006	Ye Z, Cheng K, Guntaka RV, Mahato RI. Receptor-mediated hepatic uptake of M6P-BSA-conjugated triplex-forming oligonucleotides in rats. Bioconjug Chem. 2006;17(3):823-30. doi: 10.1021/bc060006z	USA
45	2006	Lin YL, Lee TF, Huang YJ, Huang YT. Antiproliferative effect of salvianolic acid A on rat hepatic stellate cells. J Pharm Pharmacol. 2006;58(7):933-9. doi: 10.1211/jpp.58.7.0008	Taiwan
46	2006	Lin YL, Lee TF, Huang YJ, Huang YT. Inhibitory effects of Ligusticum chuanxiong on the proliferation of rat hepatic stellate cells. J Gastroenterol Hepatol. 2006;21(8):1257-65. doi: 10.1111/j.1440-1746.2006.04326.x	Taiwan
47	2006	Cheng Y, Ping J, Xu HD, Fu HJ, Zhou ZH. Synergistic effect of a novel oxymatrine-baicalin combination against hepatitis B virus replication, alpha smooth muscle actin expression and type I collagen synthesis in vitro. World J Gastroenterol.	China

		2006;12(32):5153-9. doi: 10.3748/wjg.v12.i32.5153	
48	2006	Cheng Y, Ping J, Liu C, Tan YZ, Chen GF. Study on effects of extracts from Salvia Miltiorrhiza and Curcuma Longa in inhibiting phosphorylated extracellular signal regulated kinase expression in rat's hepatic stellate cells. Chin J Integr Med. 2006;12(3):207-11. doi: 10.1007/BF02836524	China
49	2006	Oh SH, Cho KH, Yang BS, Roh YK. Natural compounds from Danshen suppress the activity of hepatic stellate cells. Arch Pharm Res. 2006;29(9):762-7. doi: 10.1007/BF02974077	Korea
50	2006	Cong M, Wang P, Liu TH, Xu Y, Lu Y, Tang SZ, Liu XM, Wang BE, Jia JD, You H. [Comparison between the suppression of tissue inhibitor of metalloproteinase-1 gene expression by recombinant adeno-associated virus carrying antisense RNA and small interfering RNA (siRNA) of TIMP-1 in rat hepatic stellate cells]. Zhonghua Gan Zang Bing Za Zhi 2006;14(10):742-7. Chinese. PMID: 17064467	China
51	2006	Zhang C, Zhuo L. Epigallocatechin gallate and genistein attenuate glial fibrillary acidic protein elevation induced by fibrogenic cytokines in hepatic stellate cells. Int J Mol Med. 2006;18(6):1141-51. PMID: 17089019	Singapore
52	2006	Zhang C, Zhu Y, Wan J, Xu H, Shi H, Lu X. Effects of Ginkgo biloba extract on cell proliferation, cytokines and extracellular matrix of hepatic stellate cells. Liver Int. 2006;26(10):1283-90. doi: 10.1111/j.1478-3231.2006.01378.x	China
53	2007	Maubach G, Lim MC, Kumar S, Zhuo L. Expression and upregulation of cathepsin S and other early molecules required for antigen presentation in activated hepatic stellate cells upon IFN-gamma treatment. Biochim Biophys Acta 2007;1773(2):219-31. doi: 10.1016/j.bbamcr.2006.11.005	Singapore
54	2007	Gui M, Zhang YF, Xiao ZY, Sun P, Dai JF, Wang SF, Rui YC, Zhang JP. Inhibitory effect of emodin on tissue inhibitor of metalloproteinases-1 (TIMP-1) expression in rat hepatic stellate cells. Dig Dis Sci. 2007;52(1):200-7. doi: 10.1007/s10620-006-9321-z	China
55	2007	Hsu YC, Chiu YT, Cheng CC, Wu CF, Lin YL, Huang YT. Antifibrotic effects of tetrandrine on hepatic stellate cells and rats with liver fibrosis. J Gastroenterol Hepatol. 2007;22(1):99-111. doi: 10.1111/j.1440-1746.2006.04361.x	Taiwan
56	2007	de Villiers WJ, Song Z, Nasser MS, Deaciuc IV, McClain CJ. 4-Hydroxynonenal- induced apoptosis in rat hepatic stellate cells: mechanistic approach. J Gastroenterol Hepatol. 2007;22(3):414-22. doi: 10.1111/j.1440-1746.2006.04625.x	USA
57	2007	Bruck R, Ashkenazi M, Weiss S, Goldiner I, Shapiro H, Aeed H, Genina O, Helpern Z, Pines M. Prevention of liver cirrhosis in rats by curcumin. Liver Int. 2007;27(3):373-83. doi: 10.1111/j.1478-3231.2007.01453.x	Israel
58	2007	Luk JM, Zhang QS, Lee NP, Wo JY, Leung PP, Liu LX, Hu MY, Cheung KF, Hui CK, Lau GK, Fan ST. Hepatic stellate cell-targeted delivery of M6P-HSA- glycyrrhetinic acid attenuates hepatic fibrogenesis in a bile duct ligation rat model. Liver Int.	China

		2007;27(4):548-57. doi: 10.1111/j.1478-3231.2007.01452.x	
59	2007	Wang LT, Ma H, Zhang P, Jia JD. [Effects of rat recombinant leptin on gene expression of collagen I and tissue inhibitor of metalloproteinase 1 in HSC-T6 cells]. Zhonghua Gan Zang Bing Za Zhi 2007;15(5):394-5. Chinese. PMID: 17524281	China
60	2007	Lee TF, Lin YL, Huang YT. Studies on antiproliferative effects of phthalides from Ligusticum chuanxiong in hepatic stellate cells. Planta Med. 2007;73(6):527-34. doi: 10.1055/s-2007-981520	Taiwan
61	2007	Tajima K, Terai S, Takami T, Kawaguchi K, Okita K, Sakaida I. Importance of inhibitor of DNA binding/differentiation 2 in hepatic stellate cell differentiation and proliferation. Hepatol Res. 2007;37(8):647-55. doi: 10.1111/j.1872-034X.2007.00089.x	Japan
62	2007	Li Y, Wu W, Jiang YF, Wang KK. [Effect of heat shock protein 47 on the expression of collagen I induced by TGF-beta(1) in hepatic stellate cell-T6 cells]. Zhong Nan Da Xue Xue Bao Yi Xue Ban 2007;32(4):650-5. Chinese. PMID: 17767059	China
63	2007	Qu Z, Lou D, Pan Y. The role of IkappaBalpha in TNF-alpha-induced apoptosis in hepatic stellate cell line HSC-T6. J Huazhong Univ Sci Technolog Med Sci. 2007;27(4):407-10. doi: 10.1007/s11596-007-0414-5	China
64	2007	Fan RH, Chen PS, Zhao D, Zhang WD. [Hypoxia induced by CoCl2 influencing the expression and the activity of matrix metalloproteinase-2 in rat hepatic stellate cells]. Zhonghua Gan Zang Bing Za Zhi 2007;15(9):654-7. Chinese. PMID: 17903365	China
65	2007	Ye Z, Guntaka RV, Mahato RI. Sequence-specific triple helix formation with genomic DNA. Biochemistry 2007;46(40):11240-52. doi: 10.1021/bi700580y	USA
66	2008	Yun HS, Do SH, Jeong WI, Yang HJ, Yuan DW, Hong IH, Lee HR, Lee IS, Kim YK, Choi MS, Kim HA, Jeong KS. Cytotoxic effects of the conjugated linoleic acid isomers t10c12, c9t11-CLA and mixed form on rat hepatic stellate cells and CCl4-induced hepatic fibrosis. J Nutr Biochem. 2008;19(3):175-83. doi: 10.1016/j.jnutbio.2007.02.007	Korea
67	2008	Wu LM, Wu XX, Sun Y, Kong XW, Zhang YH, Xu Q. A novel synthetic oleanolic acid derivative (CPU-II2) attenuates liver fibrosis in mice through regulating the function of hepatic stellate cells. J Biomed Sci. 2008;15(2):251-9. doi: 10.1007/s11373-007-9216-9	China
68	2008	Zvibel I, Bar-Zohar D, Kloog Y, Oren R, Reif S. The effect of Ras inhibition on the proliferation, apoptosis and matrix metalloproteases activity in rat hepatic stellate cells. Dig Dis Sci. 2008;53(4):1048-53. doi: 10.1007/s10620-007-9984-0	Israel
69	2008	Yang KL, Chang WT, Chuang CC, Hung KC, Li EI. Antagonizing TGF-beta induced liver fibrosis by a retinoic acid derivative through regulation of ROS and calcium influx. Biochem Biophys Res Commun. 2008;365(3):484-9. doi: 10.1016/j.bbrc.2007.10.203	Taiwan
70	2008	Bruck R, Weiss S, Traister A, Zvibel I, Aeed H, Halpern Z, Oren R. Induced hypothyroidism accelerates the regression of liver	Israel

		fibrosis in rats. J Gastroenterol Hepatol. 2007;22(12):2189-94. doi: 10.1111/j.1440-1746.2006.04777.x	
71	2008	Yang H, Sung SH, Kim YC. Antifibrotic phenanthrenes of Dendrobium nobile stems. J Nat Prod. 2007;70(12):1925-9. doi: 10.1021/np070423f	Korea
72	2008	Lee MK, Ha NR, Yang H, Sung SH, Kim GH, Kim YC. Antiproliferative activity of triterpenoids from Eclipta prostrata on hepatic stellate cells. Phytomedicine 2008;15(9):775-80. doi: 10.1016/j.phymed.2007.10.004	Korea
73	2008	Zhou X, Yu J, Li Q, Qian W, Xu KS. [Effects of transforming growth factor- beta 3 gene transfer on type I collagen synthesis of hepatic stellate cells]. Zhonghua Gan Zang Bing Za Zhi 2008;16(1):43-8. Chinese. PMID: 18226343	China
74	2008	Liu J, Gong H, Zhang ZT, Wang Y. Effect of angiotensin II and angiotensin II type 1 receptor antagonist on the proliferation, contraction and collagen synthesis in rat hepatic stellate cells. Chin Med J. (Engl) 2008;121(2):161-5. PMID: 18272044	China
75	2008	Shen YM, Zhu X, Zhang KH, Xie Y, Chen J, Dai Y, Ou-Yang CH, Li BM. [Effect of ursolic acid on proliferation and apoptosis of hepatic stellate cells in vitro]. Zhonghua Gan Zang Bing Za Zhi 2008;16(4):298-301. Chinese. PMID: 18423155	China
76	2008	Lim MC, Maubach G, Zhuo L. Glial fibrillary acidic protein splice variants in hepatic stellate cells--expression and regulation. Mol Cells 2008;25(3):376-84. PMID: 18443417	Singapore
77	2008	Yuhua Z, Wanhua R, Chenggang S, Jun S, Yanjun W, Chunqing Z. Disruption of connective tissue growth factor by short hairpin RNA inhibits collagen synthesis and extracellular matrix secretion in hepatic stellate cells. Liver Int. 2008;28(5):632-9. doi: 10.1111/j.1478-3231.2008.01730.x	China
78	2008	Li Q, Zhou X, Yu J, Qian W, Xu KS. [Influence of recombinant transforming growth factor-beta3 on collagen synthesis and deposition: experiment with rat cell model of liver fibrosis]. Zhonghua Yi Xue Za Zhi 2008;88(18):1273-8. Chinese. PMID: 18844103	China
79	2008	Li X, Meng Y, Huang ML, Zhang XL, Zhang ZS. [Angiotensin II stimulates platelet-derived growth factor-B expression in hepatic stellate cells by activating EGR-1]. Nan Fang Yi Ke Da Xue Xue Bao 2008;28(6):963-7. Chinese. PMID: 18583239	China
80	2008	Zhang XL, Li X, Xiao B, Huang ML, Meng Y, Li YF, Wang YY, Song WB. [Effect of angiotensin II on Rho-Rock pathway in rat hepatic stellate cell contraction]. Nan Fang Yi Ke Da Xue Xue Bao 2008;28(6):968-71. Chinese. PMID: 18583240	China
81	2008	Lee MK, Yang H, Yoon JS, Jeong EJ, Kim DY, Ha NR, Sung SH, Kim YC. Antifibrotic activity of diterpenes from Biota orientalis leaves on hepatic stellate cells. Arch Pharm Res. 2008;31(7):866-71. doi: 10.1007/s12272-001-1239-9	Korea
82	2008	Lin YL, Wu CF, Huang YT. Phenols from the roots of Rheum palmatum attenuate chemotaxis in rat hepatic stellate cells. Planta Med. 2008;74(10):1246-52. doi: 10.1055/s-2008-1074581	Taiwan

83	2008	Hu PF, Zhu YW, Zhong W, Chen YX, Lin Y, Zhang X, Yin C, Yue HY, Xie WF. Inhibition of plasminogen activator inhibitor-1 expression by siRNA in rat hepatic stellate cells. J Gastroenterol Hepatol. 2008;23(12):1917-25. doi: 10.1111/j.1440-1746.2008.05485.x	China
84	2008	Zhao L, Zhang C, Zhuo L, Zhang Y, Ying JY. Imidazolium salts: a mild reducing and antioxidative reagent. J Am Chem Soc. 2008;130(38):12586-7. doi: 10.1021/ja8037883	Singapore
85	2008	Li G, Li D, Xie Q, Shi Y, Jiang S, Jin Y. RNA interfering connective tissue growth factor prevents rat hepatic stellate cell activation and extracellular matrix production. J Gene Med. 2008;10(9):1039-47. doi: 10.1002/jgm.1223	China
86	2008	Zhang XL, Xiao B, Li X, Huang ML, Meng Y, Li YF, Wang YY, Song WB. [Role of Rho-Rock pathways induced by angiotensin II in hepatic stellate cell contraction]. Zhonghua Yi Xue Za Zhi 2008;88(34):2422-6. Chinese. PMID: 19087721	China
87	2008	Shyu MH, Kao TC, Yen GC. Hsian-tsao (Mesona procumbens Heml.) prevents against rat liver fibrosis induced by CCl(4) via inhibition of hepatic stellate cells activation. Food Chem Toxicol. 2008;46(12):3707-13. doi: 10.1016/j.fct.2008.09.051	Taiwan
88	2008	Deng X, Chen YX, Zhang X, Zhang JP, Yin C, Yue HY, Lin Y, Han ZG, Xie WF. Hepatic stellate cells modulate the differentiation of bone marrow mesenchymal stem cells into hepatocyte-like cells. J Cell Physiol. 2008;217(1):138-44. doi: 10.1002/jcp.21481	China
89	2008	Bruck R, Weiss S, Aeed H, Pines M, Halpern Z, Zvibel I. Additive inhibitory effect of experimentally induced hepatic cirrhosis by agonists of peroxisome proliferator activator receptor gamma and retinoic acid receptor. Dig Dis Sci. 2009;54(2):292-9. doi: 10.1007/s10620-008-0336-5	Israel
90	2008	Lin YL, Wu CF, Huang YT. Effects of rhubarb on migration of rat hepatic stellate cells. J Gastroenterol Hepatol. 2009;24(3):453-61. doi: 10.1111/j.1440-1746.2008.05573.x	Taiwan
91	2008	Li GM, Li DG, Xie Q, Zong CH, Jiang S, Lu HM. [Effects of silencing connective tissue growth factor on rat transforming growth factor beta/Smads signal]. Zhonghua Gan Zang Bing Za Zhi 2008;16(11):840-3. Chinese. PMID: 19032869	China
92	2008	Shi HY, Xu JW, Ren XX. [Effect of genistein on hepatic stellate cell proliferation and lipid peroxidation in vitro]. Nan Fang Yi Ke Da Xue Xue Bao 2008;28(11):2066-8. Chinese. PMID: 19033130	China
93	2008	Ishigaki N, Yamamoto N, Jin H, Uchida K, Terai S, Sakaida I. Continuos intravenous infusion of atrial natriuretic peptide (ANP) prevented liver fibrosis in rat. Biochem Biophys Res Commun. 2009;378(3):354-9. doi: 10.1016/j.bbrc.2008.10.154	Japan
94	2008	Wang AM, Ren CH, Xiang Y. [Growth factors may enhance c-fos and c-jun gene expressions of hepatic stellate cells]. Zhonghua Gan Zang Bing Za Zhi 2008;16(12):902-4. Chinese. PMID: 19105933	China

95	2009	Liu J, Xu XF, Yang WJ. [The effects of Hedgehog-Gli 1 signaling pathway on proliferation and apoptosis of hepatic stellate cells]. Zhonghua Gan Zang Bing Za Zhi 2009;17(1):33-7. Chinese. PMID: 19203449	China
96	2009	Yang N, Ye Z, Li F, Mahato RI. HPMA polymer-based site-specific delivery of oligonucleotides to hepatic stellate cells. Bioconjug Chem. 2009;20(2):213-21. doi: 10.1021/bc800237t	USA
97	2009	Yang L, Dan D, Zhu R, Zhou W, Qian W, Ye J, Hou X. [Beta-elemene inhibits expression of ANG II and RhoA/ROCK signaling in hepatic stellate cells]. Zhongguo Zhong Yao Za Zhi 2009;34(4):458-63. Chinese. PMID: 19459312	China
98	2009	Li X, Zhang YJ, Meng Y, Zhou GS, Zhang ZS. [Effect of angiotensin II type 1 receptor and angiotensin-converting enzyme gene silencing on nuclear factor- kappaB activity in hepatic stellate cells]. Nan Fang Yi Ke Da Xue Xue Bao 2009;29(3):402-4. Chinese. PMID: 19304510	China
99	2009	Li WT, He YW, Xiao ZH, Ma YB. [Effect of beta-catenin on the activation of hepatic stellate cells induced by transforming growth factor-beta1]. Zhonghua Gan Zang Bing Za Zhi 2009;17(3):188-92. Chinese. PMID: 19335981	China
100	2009	Ma J, Li F, Liu L, Cui D, Wu X, Jiang X, Jiang H. Raf kinase inhibitor protein inhibits cell proliferation but promotes cell migration in rat hepatic stellate cells. Liver Int. 2009;29(4):567-74. doi: 10.1111/j.1478-3231.2009.01981.x	China
101	2009	Ying SS, Li X, Huang ML, Meng Y, Zhang ZS. [Effect of angiotensin1-7 on alpha-smooth muscle actin protein expression in rat hepatic stellate cells]. Nan Fang Yi Ke Da Xue Xue Bao 2009;29(4):732-3. Chinese. PMID: 19403408	China
102	2009	Cheng K, Yang N, Mahato RI. TGF-beta1 gene silencing for treating liver fibrosis. Mol Pharm. 2009;6(3):772-9. doi: 10.1021/mp9000469	USA
103	2009	He YJ, Pan J, Shu JC, Shen Y, Lv X, Fang L. [The role and mechanisms of cyclooxygenase-2 inhibitors on the proliferation of hepatic stellate cell]. Zhonghua Gan Zang Bing Za Zhi 2009;17(5):346-9. Chinese. PMID: 19497199	China
104	2009	Yue LP, Ma H, Feng YS, Jia JD. [Effects of rat recombinant leptin on matrix metalloproteinase-2 gene expression, protein level and enzymatic activity in HSC-T6 cells]. Zhonghua Gan Zang Bing Za Zhi 2009;17(5):383-4. Chinese. PMID: 19497207	China
105	2009	Zhao ZD, Huang ZS. [Study on effects of curcumin on expressions of PDGF-BB, PDGFRbeta and ERK1 of HSC]. Zhong Yao Cai 2009;32(5):732-5. Chinese. PMID: 19771848	China
106	2009	Yang MF, Xie J, Gu XY, Zhang XH, Davey AK, Zhang SJ, Wang JP, Zhu RM. Involvement of 90-kuD ribosomal S6 kinase in collagen type I expression in rat hepatic fibrosis. World J Gastroenterol. 2009;15(17):2109-15. doi: 10.3748/wjg.15.2109	China
107	2009	Wang GJ, Huang YJ, Chen DH, Lin YL. Ganoderma lucidum extract attenuates the proliferation of hepatic stellate cells by blocking the PDGF receptor. Phytother Res. 2009;23(6):833-9. doi: 10.1002/ptr.2687	Taiwan

108	2009	Lin YL, Lin CY, Chi CW, Huang YT. Study on antifibrotic effects of curcumin in rat hepatic stellate cells. Phytother Res. 2009;23(7):927-32. doi: 10.1002/ptr.2764	Taiwan
109	2009	Wang AM, Ren CG, Ma ZJ, Xiang Y, Zhang ZW, Zhu ZJ, Wang BE. [Regulation of c-fos gene expression in hepatic stellate cells by transforming growth factor beta]. Nan Fang Yi Ke Da Xue Xue Bao 2009;29(7):1408-9. Chinese. PMID: 19620067	China
110	2009	Liu LX, Huang S, Zhang QQ, Liu Y, Zhang DM, Guo XH, Han DW. Insulin-like growth factor binding protein-7 induces activation and transdifferentiation of hepatic stellate cells in vitro. World J Gastroenterol. 2009;15(26):3246-53. doi: 10.3748/wjg.15.3246	China
111	2009	Weng TC, Shen CC, Chiu YT, Lin YL, Kuo CD, Huang YT. Inhibitory effects of armepavine against hepatic fibrosis in rats. J Biomed Sci. 2009;16(1):78. doi: 10.1186/1423-0127-16-78	Taiwan
112	2009	Qian J, Zhang JS, Wang XQ, Ji JL, Mei S. Fenretinide stimulates the apoptosis of hepatic stellate cells and ameliorates hepatic fibrosis in mice. Hepatol Res. 2009;39(12):1229-47. doi: 10.1111/j.1872-034X.2009.00562.x	China
113	2009	Tang LX, Yang G, Tang JJ. [Smad7 inhibits collagen expression in human hepatic satellite cells in vitro]. Nan Fang Yi Ke Da Xue Xue Bao 2009;29(10):2122-3, 2127. Chinese. PMID: 19861282	China
114	2009	Liu DG, Wang J, Zhang ZT, Wang Y. The urotension II antagonist SB-710411 arrests fibrosis in CCL4 cirrhotic rats. Mol Med Rep. 2009;2(6):953-61. doi: 10.3892/mmr_00000198	China
115	2009	Kim HK, Yang TH, Cho HY. Antifibrotic effects of green tea on in vitro and in vivo models of liver fibrosis. World J Gastroenterol. 2009;15(41):5200-5. doi: 10.3748/wjg.15.5200	Korea
116	2009	Shu JC, He YJ, Lv X, Zhao JR, Zhao J, Shen Y, Ye GR, Wang LX. Effect of curcumin on the proliferation and apoptosis of hepatic stellate cells. Braz J Med Biol Res. 2009;42(12):1173-8. doi: 10.1590/s0100-879x2009005000041	China
117	2009	Lee MK, Lee KY, Jeon HY, Sung SH, Kim YC. Antifibrotic activity of triterpenoids from the aerial parts of Euscaphis japonica on hepatic stellate cells. J Enzyme Inhib Med Chem. 2009;24(6):1276-9. doi: 10.3109/14756360902829709	Korea
118	2010	Wang JH, Shin JW, Son JY, Cho JH, Son CG. Antifibrotic effects of CGX, a traditional herbal formula, and its mechanisms in rats. J Ethnopharmacol. 2010;127(2):534-42. doi: 10.1016/j.jep.2009.10.001	Korea
119	2010	Dai L, Ji H, Kong XW, Zhang YH. Antifibrotic effects of ZK14, a novel nitric oxide-donating biphenyldicarboxylate derivative, on rat HSC-T6 cells and CCl4-induced hepatic fibrosis. Acta Pharmacol Sin. 2010;31(1):27-34. doi: 10.1038/aps.2009.170	China
120	2010	Wang R, Chen WH, Shi YP. ent-kaurane and ent-pimarane diterpenoids from Siegesbeckia pubescens. J Nat Prod. 2010;73(1):17-21. doi: 10.1021/np9005579	China

121	2010	Lim CS, Kim EY, Lee HS, Soh Y, Sohn Y, Kim SY, Sohn NW, Jung HS, Kim YB. Protective effects of Cinnamomum cassia Blume in the fibrogenesis of activated HSC-T6 cells and dimethylnitrosamine-induced acute liver injury in SD rats. Biosci Biotechnol Biochem. 2010;74(3):477-83. doi: 10.1271/bbb.90435	Korea
122	2010	Ge HM, Peng H, Guo ZK, Cui JT, Song YC, Tan RX. Bioactive alkaloids from the plant endophytic fungus *Aspergillus terreus*. Planta Med. 2010;76(8):822-4. doi: 10.1055/s-0029-1240726	China
123	2010	Bae MA, Rhee SD, Jung WH, Ahn JH, Song BJ, Cheon HG. Selective inhibition of activated stellate cells and protection from carbon tetrachloride-induced liver injury in rats by a new PPARgamma agonist KR62776. Arch Pharm Res. 2010;33(3):433-42. doi: 10.1007/s12272-010-0313-3	Korea
124	2010	Zhang Y, Deng Q, Hu GX, Yuan K, Yuan F, Huang YQ. [Effect of hydroxycamptothecin (HCPT) on proliferation and apoptosis of rat hepatic stellate cells]. Zhonghua Gan Zang Bing Za Zhi 2010;18(3):199-203. Chinese. doi: 10.3760/cma.j.issn.1007-3418.2010.03.012	China
125	2010	Cui D, Zhang S, Ma J, Han J, Jiang H. Short interfering RNA targetting NF- kappa B induces apoptosis of hepatic stellate cells and attenuates extracellular matrix production. Dig Liver Dis. 2010;42(11):813-7. doi: 10.1016/j.dld.2010.03.011	China
126	2010	Yu FJ, Dong PH, Fan XF, Lin Z, Chen YP, Li J. Down-regulation of angiotensin II by shRNA reduces collagen synthesis in hepatic stellate cells. Int J Mol Med. 2010;25(5):801-6. doi: 10.3892/ijmm_00000407	China
127	2010	Sun W, Gui S, Wu L, Wang H, Wei W. [Effects of Shaoqiduogan on MMP-13, TIMP-1 expression in liver and hepatic stellate cells of hepatic fibrosis rats]. Zhongguo Zhong Yao Za Zhi 2010;35(11):1447-51. Chinese. PMID: 20822018	China
128	2010	Kim KS, Hur W, Park SJ, Hong SW, Choi JE, Goh EJ, Yoon SK, Hahn SK. Bioimaging for targeted delivery of hyaluronic Acid derivatives to the livers in cirrhotic mice using quantum dots. ACS Nano. 2010;4(6):3005-14. doi: 10.1021/nn100589y	Korea
129	2010	Chen BY, Qu P, Tie R, Zhu MZ, Zhu XX, Yu J. Protecting effects of vasonatrin peptide against carbon tetrachloride-induced liver fibrosis. Regul Pept. 2010;164(2-3):139-43. doi: 10.1016/j.regpep.2010.06.007	China
130	2010	Dong P, Yu F, Fan X, Lin Z, Chen Y, Li J. Inhibition of ATIR by shRNA prevents collagen synthesis in hepatic stellate cells. Mol Cell Biochem. 2010;344(1-2):195-202. doi: 10.1007/s11010-010-0542-2	China
131	2010	Wu JH, Wang YR, Huang WY, Tan RX. Anti-proliferative and pro-apoptotic effects of tectorigenin on hepatic stellate cells. World J Gastroenterol. 2010;16(31):3911-8. doi: 10.3748/wjg.v16.i31.3911	China
132	2010	Dun ZN, Zhang XL, An JY, Zheng LB, Barrett R, Xie SR. Specific shRNA targeting of FAK influenced collagen metabolism in rat hepatic stellate cells. World J Gastroenterol. 2010;16(32):4100-6. doi: 10.3748/wjg.v16.i32.4100	China

133	2010	Li L, Li J, Wang JY, Yang CQ, Jia ML, Jiang W. Role of RhoA in platelet- derived growth factor-BB-induced migration of rat hepatic stellate cells. Chin Med J. (Engl) 2010;123(18):2502-9. PMID: 21034618	China
134	2010	Zhu L, Mahato RI. Targeted delivery of siRNA to hepatocytes and hepatic stellate cells by bioconjugation. Bioconjug Chem. 2010;21(11):2119-27. doi: 10.1021/bc100346n	USA
135	2010	He WH, Li B, Zhu X, Zhang KH, Li BM, Liu ZJ, Liu GY, Wang J. [The role and mechanism of NADPH oxidase in leptin-induced reactive oxygen species production in hepatic stellate cells]. Zhonghua Gan Zang Bing Za Zhi 2010;18(11):849-54. Chinese. doi: 10.3760/cma.j.issn.1007-3418.2010.11.014	China
136	2011	Ding H, Shi J, Wang Y, Guo J, Zhao J, Dong L. Neferine inhibits cultured hepatic stellate cell activation and facilitates apoptosis: A possible molecular mechanism. Eur J Pharmacol. 2011;650(1):163-9. doi: 10.1016/j.ejphar.2010.10.025	China
137	2011	Ping J, Li JT, Liao ZX, Shang L, Wang H. Indole-3-carbinol inhibits hepatic stellate cells proliferation by blocking NADPH oxidase/reactive oxygen species/p38 MAPK pathway. Eur J Pharmacol. 2011;650(2-3):656-62. doi: 10.1016/j.ejphar.2010.10.057	China
138	2011	Choi JH, Hwang YP, Park BH, Choi CY, Chung YC, Jeong HG. Anthocyanins isolated from the purple-fleshed sweet potato attenuate the proliferation of hepatic stellate cells by blocking the PDGF receptor. Environ Toxicol Pharmacol. 2011;31(1):212-9. doi: 10.1016/j.etap.2010.10.011	Korea
139	2011	Shin E, Lee C, Sung SH, Kim YC, Hwang BY, Lee MK. Antifibrotic activity of coumarins from Cnidium monnieri fruits in HSC-T6 hepatic stellate cells. J Nat Med. 2011;65(2):370-4. doi: 10.1007/s11418-010-0485-7	Korea
140	2011	Ming-Ju H, Yih-Shou H, Tzy-Yen C, Hui-Ling C. Hepatitis C virus E2 protein induce reactive oxygen species (ROS)-related fibrogenesis in the HSC-T6 hepatic stellate cell line. J Cell Biochem. 2011;112(1):233-43. doi: 10.1002/jcb.22926	China
141	2011	Zhang JJ, Wang YL, Feng XB, Song XD, Liu WB. Rosmarinic acid inhibits proliferation and induces apoptosis of hepatic stellate cells. Biol Pharm Bull. 2011;34(3):343-8. doi: 10.1248/bpb.34.343	China
142	2011	Li JF, Lu GF, Zou YY. Demethylbellidifolin inhibits proliferation and activation of hepatic stellate cells. J Invest Surg. 2011;24(4):171-7. doi: 10.3109/08941939.2011.568593	China
143	2011	Li J, Fan R, Zhao S, Liu L, Guo S, Wu N, Zhang W, Chen P. Reactive oxygen species released from hypoxic hepatocytes regulates MMP-2 expression in hepatic stellate cells. Int J Mol Sci. 2011;12(4):2434-47. doi: 10.3390/ijms12042434	China
144	2011	Foo NP, Lin SH, Lee YH, Wu MJ, Wang YJ. α-Lipoic acid inhibits liver fibrosis through the attenuation of ROS-triggered signaling in hepatic stellate cells activated by PDGF and TGF-β. Toxicology 2011;282(1-2):39-46. doi: 10.1016/j.tox.2011.01.009	Taiwan
145	2011	Renga B, Mencarelli A, Migliorati M, Cipriani S, D'Amore C, Distrutti E, Fiorucci S. SHP-dependent and -independent	Italy

		induction of peroxisome proliferator- activated receptor-γ by the bile acid sensor farnesoid X receptor counter- regulates the pro-inflammatory phenotype of liver myofibroblasts. Inflamm Res. 2011;60(6):577-87. doi: 10.1007/s00011-010-0306-1	
146	2011	Bartalis J, Halaweish FT. In vitro and QSAR studies of cucurbitacins on HepG2 and HSC-T6 liver cell lines. Bioorg Med Chem. 2011;19(8):2757-66. doi: 10.1016/j.bmc.2011.01.037	USA
147	2011	Yang H, Jeong EJ, Kim J, Sung SH, Kim YC. Antiproliferative triterpenes from the leaves and twigs of Juglans sinensis on HSC-T6 cells. J Nat Prod. 2011;74(4):751-6. doi: 10.1021/np1008202	Korea
148	2011	Pang W, Zhang Y, Wang S, Jia A, Dong W, Cai C, Hua Z, Zhang J. The mPlrp2 and mClps genes are involved in the hydrolysis of retinyl esters in the mouse liver. J Lipid Res. 2011;52(5):934-41. doi: 10.1194/jlr.M010082	China
149	2011	Tsai MK, Lin YL, Huang YT. Differential inhibitory effects of salvianolic acids on activation of rat hepatic stellate cells by platelet-derived growth factor. Planta Med. 2011;77(13):1495-503. doi: 10.1055/s-0030-1270783	Taiwan
150	2011	Wang XD, Gao ZH, Xue X, Cheng YN, Yue P, Fang XW, Qu XJ. N1-acetyl substituted pyrrolidine derivative CIP-A5: a novel compound that could ameliorate liver cirrhosis through modulation of hepatic stellate cell activity. Toxicol In Vitro 2011;25(4):897-904. doi: 10.1016/j.tiv.2011.02.011	China
151	2011	Yang N, Mahato RI. GFAP promoter-driven RNA interference on TGF-β1 to treat liver fibrosis. Pharm Res. 2011;28(4):752-61. doi: 10.1007/s11095-011-0384-y	USA
152	2011	Fu R, Wu J, Ding J, Sheng J, Hong L, Sun Q, Fang H, Xiang D. Targeting transforming growth factor βRII expression inhibits the activation of hepatic stellate cells and reduces collagen synthesis. Exp Biol Med. (Maywood) 2011;236(3):291-7. doi: 10.1258/ebm.2010.010231	China
153	2011	Huang HC, Lin YC, Fazary AE, Lo IW, Liaw CC, Huang YZ, Liou SS, Shen YC. New and bioactive lignans from the fruits of Schisandra sphenanthera. Food Chem. 2011;128(2):348-57. doi: 10.1016/j.foodchem.2011.03.030	Taiwan
154	2011	Wu XX, Wu LM, Fan JJ, Qin Y, Chen G, Wu XF, Shen Y, Sun Y, Xu Q. Cortex Dictamni extract induces apoptosis of activated hepatic stellate cells via STAT1 and attenuates liver fibrosis in mice. J Ethnopharmacol. 2011;135(1):173-8. doi: 10.1016/j.jep.2011.03.010	China
155	2011	Li W, Zhu C, Chen X, Li Y, Gao R, Wu Q. Pokeweed antiviral protein down- regulates Wnt/β-catenin signalling to attenuate liver fibrogenesis in vitro and in vivo. Dig Liver Dis. 2011;43(7):559-66. doi: 10.1016/j.dld.2011.02.016	China
156	2011	Lee M, Lee MK, Kim YC, Sung SH. Antifibrotic constituents of Alnus firma on hepatic stellate cells. Bioorg Med Chem Lett. 2011;21(10):2906-10. doi: 10.1016/j.bmcl.2011.03.074	Korea

157	2011	Qian KL, Xu N, Lang Q, Qi JH, Sun YC, Xiao L, Liu Q, Shi XF. [Construction and identification of siRNA eukaryotic expression vectors targeting on TGFβ1, TIMP-1 and TIMP-2 genes in vitro]. Zhonghua Gan Zang Bing Za Zhi 2011;19(4):291-6. Chinese. doi: 10.3760/cma.j.issn.1007-3418.2011.04.014	China
158	2011	Shukla RS, Qin B, Wan YJ, Cheng K. PCBP2 siRNA reverses the alcohol-induced pro-fibrogenic effects in hepatic stellate cells. Pharm Res. 2011;28(12):3058-68. doi: 10.1007/s11095-011-0475-9	USA
159	2011	Li J, Liu P, Zhang R, Cao L, Qian H, Liao J, Xu W, Wu M, Yin Z. Icaritin induces cell death in activated hepatic stellate cells through mitochondrial activated apoptosis and ameliorates the development of liver fibrosis in rats. J Ethnopharmacol. 2011;137(1):714-23. doi: 10.1016/j.jep.2011.06.030	China
160	2011	Chong LW, Hsu YC, Chiu YT, Yang KC, Huang YT. Antifibrotic effects of triptolide on hepatic stellate cells and dimethylnitrosamine-intoxicated rats. Phytother Res. 2011;25(7):990-9. doi: 10.1002/ptr.3381	Taiwan
161	2011	Chuang HY, Ng LT, Lin LT, Chang JS, Chen JY, Lin TC, Lin CC. Hydrolysable tannins of tropical almond show antifibrotic effects in TGF-β1-induced hepatic stellate cells. J Sci Food Agric. 2011;91(15):2777-84. doi: 10.1002/jsfa.4521	Taiwan
162	2011	Zhang XL, Xiao B, Meng Y, Li X. [Aldosterone stimulates hepatic stellate cells contraction via Ca2+-independent pathways]. Zhonghua Gan Zang Bing Za Zhi 2011;19(7):537-41. Chinese. doi: 10.3760/cma.j.issn.1007-3418.2011.07.016	China
163	2011	Ping J, Gao AM, Xu D, Li RW, Wang H. [Therapeutic effect of indole-3-carbinol on pig serum-induced hepatic fibrosis in rats]. Yao Xue Xue Bao 2011;46(8):915-21. Chinese. PMID: 22007515	China
164	2011	Ping J, Gao AM, Qin HQ, Wei XN, Bai J, Liu L, Li XH, Li RW, Ao Y, Wang H. Indole-3-carbinol enhances the resolution of rat liver fibrosis and stimulates hepatic stellate cell apoptosis by blocking the inhibitor of κB kinase α/inhibitor of κB-α/nuclear factor-κB pathway. J Pharmacol Exp Ther. 2011;339(2):694-703. doi: 10.1124/jpet.111.179820. *Retraction in: J Pharmacol Exp Ther. 2012;342(1):233.* PMID: 21862660.	China
165	2011	Lu P, Liu H, Yin H, Yang L. Expression of angiotensinogen during hepatic fibrogenesis and its effect on hepatic stellate cells. Med Sci Monit. 2011;17(9):BR248-56. doi: 10.12659/msm.881928	China
166	2011	Chin YW, Shin E, Hwang BY, Lee MK. Antifibrotic constituents from Garcinia mangostana. Nat Prod Commun. 2011;6(9):1267-8. PMID: 21941895	Korea
167	2011	Lo YT, Tsai YH, Wu SJ, Chen JR, Chao JC. Ginsenoside Rb1 inhibits cell activation and liver fibrosis in rat hepatic stellate cells. J Med Food. 2011;14(10):1135-43. doi: 10.1089/jmf.2010.1485	Taiwan
168	2011	Deng L, Li Y, Huang JM, Zhou GY, Li Q, Qian W, Xu KS. [Effects of exogenous transforming growth factor-β3 on the activities of its promoter and cAMP- responsive element binding protein-1 in rat hepatic stellate cell]. Zhonghua Yi Xue Za Zhi	China

		2011;91(33):2366-9. Chinese. PMID: 22321755	
169	2011	Ge WS, Wu JX, Fan JG, Wang YJ, Chen YW. Inhibition of high-mobility group box 1 expression by siRNA in rat hepatic stellate cells. World J Gastroenterol. 2011;17(36):4090-8. doi: 10.3748/wjg.v17.i36.4090	China
170	2011	He X, Lv R, Wang K, Huang X, Wu W, Yin L, Liu Y. Cytoglobin exhibits anti- fibrosis activity on liver in vivo and in vitro. Protein J. 2011;30(7):437-46. doi: 10.1007/s10930-011-9340-2	China
171	2011	Li Y, Deng L, Qian W, Zhou JN, Xu KS. [Effects of exogenous TGF-β3 on the expression of endogenous TGF-β3 in hepatic stellate cell-T6 (HSC-T6)]. Zhonghua Gan Zang Bing Za Zhi 2011;19(11):843-7. Chinese. doi: 10.3760/cma.j.issn.1007-3418.2011.11.012	China
172	2011	Liu J, Wang CY, Chen YP, Lin Z, Yang T, Lu XJ. [Dynamic expression of tropomyosin 1 in rat model of hepatic fibrosis and hepatic stellate cells]. Zhonghua Gan Zang Bing Za Zhi 2011;19(11):848-52. Chinese. doi: 10.3760/cma.j.issn.1007-3418.2011.11.013	China
173	2011	Weng TC, Shen CC, Chiu YT, Lin YL, Huang YT. Effects of armepavine against hepatic fibrosis induced by thioacetamide in rats. Phytother Res. 2012;26(3):344-53. doi: 10.1002/ptr.3539	Taiwan
174	2012	Yang H, Lee PJ, Jeong EJ, Kim HP, Kim YC. Selective apoptosis in hepatic stellate cells mediates the antifibrotic effect of phenanthrenes from Dendrobium nobile. Phytother Res. 2012;26(7):974-80. doi: 10.1002/ptr.3632	Korea
175	2012	Pan TL, Wang PW, Leu YL, Wu TH, Wu TS. Inhibitory effects of Scutellaria baicalensis extract on hepatic stellate cells through inducing G2/M cell cycle arrest and activating ERK-dependent apoptosis via Bax and caspase pathway. J Ethnopharmacol. 2012;139(3):829-37. doi: 10.1016/j.jep.2011.12.028. Erratum in: J Ethnopharmacol. 2015;168:381. PMID: 22210104	Taiwan
176	2012	Tang LX, He RH, Yang G, Tan JJ, Zhou L, Meng XM, Huang XR, Lan HY. Asiatic acid inhibits liver fibrosis by blocking TGF-beta/Smad signaling in vivo and in vitro. PLoS One 2012;7(2):e31350. doi: 10.1371/journal.pone.0031350	China
177	2012	Chen H, Zhou Y, Chen KQ, An G, Ji SY, Chen QK. Anti-fibrotic effects via regulation of transcription factor Sp1 on hepatic stellate cells. Cell Physiol Biochem. 2012;29(1-2):51-60. doi: 10.1159/000337586	China
178	2012	Chou MH, Chuang JH, Eng HL, Tsai PC, Hsieh CS, Liu HC, Wang CH, Lin CY, Lin TM. Effects of hepatocyte CD14 upregulation during cholestasis on endotoxin sensitivity. PLoS One 2012;7(4):e34903. doi: 10.1371/journal.pone.0034903	Taiwan
179	2012	Lee M, Park JH, Min DS, Yoo H, Park JH, Kim YC, Sung SH. Antifibrotic activity of diarylheptanoids from Betula platyphylla toward HSC-T6 cells. Biosci Biotechnol Biochem. 2012;76(9):1616-20. doi: 10.1271/bbb.110887	Korea
180	2012	Chen Y, Zheng S, Qi D, Zheng S, Guo J, Zhang S, Weng Z. Inhibition of Notch signaling by a γ-secretase inhibitor attenuates	China

		hepatic fibrosis in rats. PLoS One 2012;7(10):e46512. doi: 10.1371/journal.pone.0046512	
181	2012	Wan Y, Wu YL, Lian LH, Xie WX, Li X, Ouyang BQ, Bai T, Li Q, Yang N, Nan JX. The anti-fibrotic effect of betulinic acid is mediated through the inhibition of NF-κB nuclear protein translocation. Chem Biol Interact. 2012;195(3):215-23. doi: 10.1016/j.cbi.2012.01.002	China
182	2012	Wang B, Li W, Guo K, Xiao Y, Wang Y, Fan J. miR-181b promotes hepatic stellate cells proliferation by targeting p27 and is elevated in the serum of cirrhosis patients. Biochem Biophys Res Commun. 2012;421(1):4-8. doi: 10.1016/j.bbrc.2012.03.025	China
183	2012	Chen YX, Weng ZH, Zhang SL. Notch3 regulates the activation of hepatic stellate cells. World J Gastroenterol. 2012;18(12):1397-403. doi: 10.3748/wjg.v18.i12.1397	China
184	2012	Li L, Wang JY, Yang CQ, Jiang W. Effect of RhoA on transforming growth factor β1-induced rat hepatic stellate cell migration. Liver Int. 2012;32(7):1093-102. doi: 10.1111/j.1478-3231.2012.02809.x	China
185	2012	Tao LL, Cheng YY, Ding D, Mei S, Xu JW, Yu J, Ou-Yang Q, Deng L, Chen Q, Li QQ, Xu ZD, Liu XP. C/EBP-α ameliorates CCl(4)-induced liver fibrosis in mice through promoting apoptosis of hepatic stellate cells with little apoptotic effect on hepatocytes in vitro and in vivo. Apoptosis 2012;17(5):492-502. doi: 10.1007/s10495-012-0700-y	China
186	2012	Qin S, Jiang H, Su S, Wang D, Liang Z, Zhang J, Yang W. Inhibition of hepatic stellate cell proliferation by bone marrow mesenchymal stem cells via regulation of the cell cycle in rat. Exp Ther Med. 2012;4(3):375-80. doi: 10.3892/etm.2012.628	China
187	2012	Hao LS, Zhang XL, Wang J, Li LW, Mo YB, Zhang C, Yao DM, Jiang HQ. [The mechanisms of inhibitory effect of adenovirus-mediated wild-type PTEN gene on the proliferation in activated hepatic stellate cells in vitro]. Zhonghua Gan Zang Bing Za Zhi 2012;20(7):503-6. Chinese. doi: 10.3760/cma.j.issn.1007-3418.2012.07.005	China
188	2012	Zhang L, Zhao LF, Yang H, Zhao JJ. [Effect of reduced glutathione on proliferation of rat hepatic stellate cells and the Nrf2/HO-1 signaling pathway]. Zhonghua Gan Zang Bing Za Zhi. 2012;20(7):507-11. Chinese. doi: 10.3760/cma.j.issn.1007-3418.2012.07.006	China
189	2012	Bian EB, Huang C, Ma TT, Tao H, Zhang H, Cheng C, Lv XW, Li J. DNMT1-mediated PTEN hypermethylation confers hepatic stellate cell activation and liver fibrogenesis in rats. Toxicol Appl Pharmacol. 2012;264(1):13-22. doi: 10.1016/j.taap.2012.06.022	China
190	2012	Yang YP, Guan XQ, Qi MM, Zhu LR. [Effects of resistin on hepatic fibrosis: possible mechanisms in non-alcoholic fatty liver disease in in vitro and in vivo]. Dongwuxue Yanjiu 2012;33(4):367-72. Chinese. doi: 10.3724/SP.J.1141.2012.04367	China
191	2012	Jiang YF, Sun HL, Zhang JJ, Huang F, Liu JQ. [Effect of shRNA-mediated silencing of CTGF and TIMP-1 on mRNA expression of CTGF, TIMP-1, and PC I and secretion of extracellular matrix in rat hepatic stellate cells]. Zhonghua Gan Zang Bing Za Zhi	China

		2012;20(8):576-80. Chinese. doi: 10.3760/cma.j.issn.1007-3418.2012.08.007	
192	2012	Chen YX, Weng ZH, Qi D, Zhang SL. [Effect of Notch signaling on the activation of hepatic stellate cells]. Zhonghua Gan Zang Bing Za Zhi 2012;20(9):677-82. Chinese. doi: 10.3760/cma.j.issn.1007-3418.2012.09.008	China
193	2012	Zhang L, Wu T, Chen JM, Yang LL, Song HY, Ji G. Danshensu inhibits acetaldehyde-induced proliferation and activation of hepatic stellate cell-T6. Zhong Xi Yi Jie He Xue Bao 2012;10(10):1155-61. doi: 10.3736/jcim20121013	China
194	2012	Yang H, Yoo G, Kim HS, Kim JY, Kim SO, Yoo YH, Sung SH. Implication of the stereoisomers of ginsenoside derivatives in the antiproliferative effect of HSC-T6 cells. J Agric Food Chem. 2012;60(47):11759-64. doi: 10.1021/jf303714c	Korea
195	2012	Zhan Q, Zhang F, Sun L, Wu Z, Chen W. Two new oleanane-type triterpenoids from Platycodi Radix and anti-proliferative activity in HSC-T6 cells. Molecules 2012;17(12):14899-907. doi: 10.3390/molecules171214899	China
196	2013	Fan HN, Wang HJ, Yang-Dan CR, Ren L, Wang C, Li YF, Deng Y. Protective effects of hydrogen sulfide on oxidative stress and fibrosis in hepatic stellate cells. Mol Med Rep. 2013;7(1):247-53. doi: 10.3892/mmr.2012.1153	China
197	2013	Chen YC, Liaw CC, Cheng YB, Lin YC, Chen CH, Huang YT, Liou SS, Chen SY, Chien CT, Lee GC, Shen YC. Anti-liver fibrotic lignans from the fruits of Schisandra arisanensis and Schisandra sphenanthera. Bioorg Med Chem Lett. 2013;23(3):880-5. doi: 10.1016/j.bmcl.2012.11.040	Taiwan
198	2013	Liu Q, Ahn JH, Kim SB, Lee C, Hwang BY, Lee MK. Sesquiterpene lactones from the roots of Lindera strychnifolia. Phytochemistry 2013;87:112-8. doi: 10.1016/j.phytochem.2012.11.004	Korea
199	2013	Ding Z, Zhuo L. Attenuation of hepatic fibrosis by an imidazolium salt in thioacetamide-induced mouse model. J Gastroenterol Hepatol. 2013;28(1):188-201. doi: 10.1111/j.1440-1746.2012.07265.x	Singapore
200	2013	Luo Z, Liu H, Sun X, Guo R, Cui R, Ma X, Yan M. RNA interference against discoidin domain receptor 2 ameliorates alcoholic liver disease in rats. PLoS One 2013;8(2):e55860. doi: 10.1371/journal.pone.0055860	China
201	2013	Wang Q, Du H, Li M, Li Y, Liu S, Gao P, Zhang X, Cheng J. MAPK signal transduction pathway regulation: A novel mechanism of rat HSC-T6 cell apoptosis induced by FUZHENGHUAYU tablet. Evid Based Complement Alternat Med. 2013;2013:368103. doi: 10.1155/2013/368103	China
202	2013	Wang Y, Huang Y, Guan F, Xiao Y, Deng J, Chen H, Chen X, Li J, Huang H, Shi C. Hypoxia-inducible factor-1alpha and MAPK co-regulate activation of hepatic stellate cells upon hypoxia stimulation. PLoS One 2013;8(9):e74051. doi: 10.1371/journal.pone.0074051	China
203	2013	Fan X, Zhang Q, Li S, Lv Y, Su H, Jiang H, Hao Z. Attenuation of CCl4-induced hepatic fibrosis in mice by vaccinating against	China

		TGF-β1. PLoS One 2013;8(12):e82190. doi: 10.1371/journal.pone.0082190	
204	2013	Narmada BC, Chia SM, Tucker-Kellogg L, Yu H. HGF regulates the activation of TGF-β1 in rat hepatocytes and hepatic stellate cells. J Cell Physiol. 2013;228(2):393-401. doi: 10.1002/jcp.24143	Singapore
205	2013	Wang YP, He Q, Wu F, Zhu LL, Liu W, Zhang YN, He YW. [Effects of Wnt3a on proliferation, activation and the expression of TGFb/Smad in rat hepatic stellate cells]. Zhonghua Gan Zang Bing Za Zhi. 2013;21(2):111-5. Chinese. doi: 10.3760/cma.j.issn.1007-3418.2013.02.009	China
206	2013	Fan HN, Wang HJ, Ren L, Ren B, Dan CR, Li YF, Hou LZ, Deng Y. Decreased expression of p38 MAPK mediates protective effects of hydrogen sulfide on hepatic fibrosis. Eur Rev Med Pharmacol Sci. 2013;17(5):644-52. PMID: 23543448	China
207	2013	Tang Y, Hu C, Liu Y. Effect of bioactive peptide of Carapax Trionycis on TGF-β1-induced intracellular events in hepatic stellate cells. J Ethnopharmacol. 2013;148(1):69-73. doi: 10.1016/j.jep.2013.03.067	China
208	2013	Zan Y, Zhang Y, Tien P. Hepatitis B virus e antigen induces activation of rat hepatic stellate cells. Biochem Biophys Res Commun. 2013;435(3):391-6. doi: 10.1016/j.bbrc.2013.04.098	China
209	2013	Liu L, Fan H, Qi P, Mei Y, Zhou L, Cai L, Lin X, Lin J. Synthesis and hepatoprotective properties of Acanthus ilicifolius alkaloid A and its derivatives. Exp Ther Med. 2013;6(3):796-802. doi: 10.3892/etm.2013.1189	China
210	2013	Zhang WY, Li Y, Li T, Ning ZW, Li W, Li X. [Aldosterone antagonist inhibits fibrosis-induced NOX4 protein expression in hepatic cells and tissues of rats]. Zhonghua Gan Zang Bing Za Zhi 2013;21(7):519-23. Chinese. doi: 10.3760/cma.j.issn.1007-3418.2013.07.011	China
211	2013	Li XM, Dong L, Shi HT, Gao TJ, Jia M. [Effect of neferine on hepatic stellate cells in collagen-I, TIMP-1 and MMP-2]. Zhongguo Zhong Yao Za Zhi 2013;38(13):2206-9. Chinese. PMID: 24079255	China
212	2013	Bai JX, Dai L, Chen HG, Xu H, Yin RL, Han J, Yuan HL. [Study on rational daily administration frequency of Fufang Biejia Ruangan tablet based on integrated serum pharmacologic and pharmacokinetic model]. Zhongguo Zhong Yao Za Zhi 2013;38(14):2394-8. Chinese. PMID: 24199579	China
213	2013	Xu L, Zheng N, He Q, Li R, Zhang K, Liang T. Puerarin, isolated from Pueraria lobata (Willd.), protects against hepatotoxicity via specific inhibition of the TGF-β1/Smad signaling pathway, thereby leading to anti- fibrotic effect. Phytomedicine 2013;20(13):1172-9. doi: 10.1016/j.phymed.2013.06.008	China
214	2013	Ren ZP, Sun LP, Xia YC, Tong QX. Effect of the protease inhibitor MG132 on the transforming growth factor-β/Smad signaling pathway in HSC-T6 cells. J Huazhong Univ Sci Technolog Med Sci. 2013;33(4):501-4. doi: 10.1007/s11596-013-1149-0	China

215	2013	Liu M, He Y, Zhang J. [Effect of autophagy inhibitor 3-methyladenine on proliferation and activation of hepatic stellate cells]. Xi Bao Yu Fen Zi Mian Yi Xue Za Zhi 2013;29(8):809-12. Chinese. PMID: 23948405	China
216	2013	Fang L, Zhan S, Huang C, Cheng X, Lv X, Si H, Li J. TRPM7 channel regulates PDGF-BB-induced proliferation of hepatic stellate cells via PI3K and ERK pathways. Toxicol Appl Pharmacol. 2013;272(3):713-25. doi: 10.1016/j.taap.2013.08.009	China
217	2013	Yu HC, Bai L, Yue SQ, Wang DS, Wang L, Han H, Dou KF. Notch signal protects non-parenchymal cells from ischemia/reperfusion injury in vitro by repressing ROS. Ann Hepatol. 2013;12(5):815-21. PMID: 24018501	China
218	2013	Chen MF, Huang CC, Liu PS, Chen CH, Shiu LY. Saikosaponin a and saikosaponin d inhibit proliferation and migratory activity of rat HSC-T6 cells. J Med Food. 2013;16(9):793-800. doi: 10.1089/jmf.2013.2762	Taiwan
219	2013	Li Y, Xia JY, Chen W, Deng CL. [Effects of Ling Qi Juan Gan capsule drug- containing serum on PDGF-induced proliferation and JAK/STAT signaling of HSC-T6 cells]. Zhonghua Gan Zang Bing Za Zhi 2013;21(9):663-7. Chinese. doi: 10.3760/cma.j.issn.1007-3418.2013.09.005	China
220	2013	Ohtera A, Miyamae Y, Nakai N, Kawachi A, Kawada K, Han J, Isoda H, Neffati M, Akita T, Maejima K, Masuda S, Kambe T, Mori N, Irie K, Nagao M. Identification of 6-octadecynoic acid from a methanol extract of Marrubium vulgare L. as a peroxisome proliferator-activated receptor γ agonist. Biochem Biophys Res Commun. 2013;440(2):204-9. doi: 10.1016/j.bbrc.2013.09.003	Japan
221	2013	Zhang Z, Gao Z, Hu W, Yin S, Wang C, Zang Y, Chen J, Zhang J, Dong L. 3,3'-Diindolylmethane ameliorates experimental hepatic fibrosis via inhibiting miR-21 expression. Br J Pharmacol. 2013;170(3):649-60. doi: 10.1111/bph.12323	China
222	2013	Hong IH, Park SJ, Goo MJ, Lee HR, Park JK, Ki MR, Kim SH, Lee EM, Kim AY, Jeong KS. JNK1 and JNK2 regulate α-SMA in hepatic stellate cells during CCl4 -induced fibrosis in the rat liver. Pathol Int. 2013;63(10):483-91. doi: 10.1111/pin.12094	Korea
223	2013	Feng YJ, Wang YJ, Zhang FC, Zhou C, Quan QZ. [Effect of Hanfangji compound on proliferation of hepatic stellate cells and expressions of TTR, ITIH1 and SERPINF2]. Zhongguo Zhong Yao Za Zhi 2013;38(19):3338-42. Chinese. PMID: 24422404	China
224	2013	Li Y, Luo Y, Zhang X, Lin X, He M, Liao M. Combined taurine, epigallocatechin gallate and genistein therapy reduces HSC-T6 cell proliferation and modulates the expression of fibrogenic factors. Int J Mol Sci. 2013;14(10):20543-54. doi: 10.3390/ijms141020543. Erratum in: Int J Mol Sci. 2021;22(5): PMID: 24129183	China
225	2014	Hao C, Xie Y, Peng M, Ma L, Zhou Y, Zhang Y, Kang W, Wang J, Bai X, Wang P, Jia Z. Inhibition of connective tissue growth factor suppresses hepatic stellate cell activation in vitro and prevents liver fibrosis in vivo. Clin Exp Med. 2014;14(2):141-50. doi: 10.1007/s10238-013-0229-6	China

226	2014	Li B, Meng X, Zhu L, Jiao X, Zhang J. Application of high-speed counter- current chromatography for isolation of triterpenes from Schisandra Chinensis (Turcz.) Baill and induction apoptosis mechanism of HSC-T6. Biomed Mater Eng. 2014;24(1):969-77. doi: 10.3233/BME-130892	China
227	2014	Dong Y, Qu Y, Xu M, Wang X, Lu L. Catalase ameliorates hepatic fibrosis by inhibition of hepatic stellate cells activation. Front Biosci. (Landmark Ed) 2014;19:535-41. doi: 10.2741/4224	China
228	2014	Jian YC, Wang JJ, Dong S, Hu JW, Hu LJ, Yang GM, Zheng YX, Xiong WJ. Wnt- induced secreted protein 1/CCN4 in liver fibrosis both in vitro and in vivo. Clin Lab. 2014;60(1):29-35. doi: 10.7754/clin.lab.2013.121035	China
229	2014	Wang H, Guan W, Yang W, Wang Q, Zhao H, Yang F, Lv X, Li J. Caffeine inhibits the activation of hepatic stellate cells induced by acetaldehyde via adenosine A2A receptor mediated by the cAMP/PKA/SRC/ERK1/2/P38 MAPK signal pathway. PLoS One 2014;9(3):e92482. doi: 10.1371/journal.pone.0092482	China
230	2014	Zong Y, Zhong M, Li DM, Zhang BJ, Mai ZP, Huo XK, Huang SS, Zhang HL, Wang C, Ma XC, Yu SM, Yang DA. Phenolic constituents from the roots of Phyllodium pulchellum. J Asian Nat Prod Res. 2014;16(7):741-6. doi: 10.1080/10286020.2014.910197	China
231	2014	Tsai TH, Shih SC, Ho TC, Ma HI, Liu MY, Chen SL, Tsao YP. Pigment epithelium-derived factor 34-mer peptide prevents liver fibrosis and hepatic stellate cell activation through down-regulation of the PDGF receptor. PLoS One 2014;9(4):e95443. doi: 10.1371/journal.pone.0095443. Erratum in: PLoS One 2014;9(9): doi: 10.1371/journal.pone.0108835	China
232	2014	Song Y, Zhan L, Yu M, Huang C, Meng X, Ma T, Zhang L, Li J. TRPV4 channel inhibits TGF-β1-induced proliferation of hepatic stellate cells. PLoS One 2014;9(7):e101179. doi: 10.1371/journal.pone.0101179	China
233	2014	Liu YW, Huang YT. Inhibitory effect of tanshinone IIA on rat hepatic stellate cells. PLoS One 2014;9(7):e103229. doi: 10.1371/journal.pone.0103229	Taiwan
234	2014	Yang MC, Wang CJ, Liao PC, Yen CJ, Shan YS. Hepatic stellate cells secretes type I collagen to trigger epithelial mesenchymal transition of hepatoma cells. Am J Cancer Res. 2014;4(6):751-63. PMID: 25520865	China
235	2014	Wang C, Zhong M, Zhang BJ, Huo XK, Huang SS, Yu SM, Ma XC. [Chemical constituents against hepatic fibrosis from Phyllodium pulchellum roots]. Zhong Yao Cai 2014;37(3):424-7. Chinese. PMID: 25174106	China
236	2014	Cui X, Zhang X, Yin Q, Meng A, Su S, Jing X, Li H, Guan X, Li X, Liu S, Cheng M. F-actin cytoskeleton reorganization is associated with hepatic stellate cell activation. Mol Med Rep. 2014;9(5):1641-7. doi: 10.3892/mmr.2014.2036	China
237	2014	Kuo LM, Kuo CY, Lin CY, Hung MF, Shen JJ, Hwang TL. Intracellular glutathione depletion by oridonin leads to apoptosis in	Taiwan

		hepatic stellate cells. Molecules 2014;19(3):3327-44. doi: 10.3390/molecules19033327	
238	2014	Bohanon FJ, Wang X, Ding C, Ding Y, Radhakrishnan GL, Rastellini C, Zhou J, Radhakrishnan RS. Oridonin inhibits hepatic stellate cell proliferation and fibrogenesis. J Surg Res. 2014;190(1):55-63. doi: 10.1016/j.jss.2014.03.036	USA
239	2014	Su KY, Hsieh CY, Chen YW, Chuang CT, Chen CT, Chen YS. Taiwanese Green Propolis and Propolin G Protect the Liver from the Pathogenesis of Fibrosis via Eliminating TGF-β-Induced Smad2/3 Phosphorylation. J Agric Food Chem. 2014;62(14):3192-201. doi: 10.1021/jf500096c	Taiwan
240	2014	Ge WS, Wang YJ, Wu JX, Fan JG, Chen YW, Zhu L. β-catenin is overexpressed in hepatic fibrosis and blockage of Wnt/β-catenin signaling inhibits hepatic stellate cell activation. Mol Med Rep. 2014;9(6):2145-51. doi: 10.3892/mmr.2014.2099	China
241	2014	Wang F, Liu S, DU T, Chen H, Li Z, Yan J. NF-κB inhibition alleviates carbon tetrachloride-induced liver fibrosis via suppression of activated hepatic stellate cells. Exp Ther Med. 2014;8(1):95-9. doi: 10.3892/etm.2014.1682	China
242	2014	Wang XY, Chen GR, Deng ZY, Zhao J, Ge JF, Li N, Chen FH. [Chemical constituents from Bidens bipinnata]. Zhongguo Zhong Yao Za Zhi 2014;39(10):1838-44. Chinese. PMID: 25282892	China
243	2014	Bai T, Yao YL, Jin XJ, Lian LH, Li Q, Yang N, Jin Q, Wu YL, Nan JX. Acanthoic acid, a diterpene in Acanthopanax koreanum, ameliorates the development of liver fibrosis via LXRs signals. Chem Biol Interact. 2014;218:63-70. doi: 10.1016/j.cbi.2014.04.016	China
244	2014	Chen J, Liu DG, Yang G, Kong LJ, Du YJ, Wang HY, Li FD, Pei FH, Song JT, Fan YJ, Liu AY, Wang XH, Li BX. Endostar, a novel human recombinant endostatin, attenuates liver fibrosis in CCl4-induced mice. Exp Biol Med (Maywood) 2014;239(8):998-1006. doi: 10.1177/1535370214532595	China
245	2014	Wang H, Ren Q, Chen W, Li R, Song L, Zhang N, Qi C, Zheng Y. [Role of PI3K/Akt signaling in hydrogen sulfide-induced alteration in expression of collagen I and III in hepatic stellate cells]. Zhonghua Gan Zang Bing Za Zhi 2014;22(6):430-3. Chinese. doi: 10.3760/cma.j.issn.1007-3418.2014.06.007	China
246	2014	Zhang Y, Zhang QQ, Guo XH, Zhang HY, Liu LX. IGFBPrP1 induces liver fibrosis by inducing hepatic stellate cell activation and hepatocyte apoptosis via Smad2/3 signaling. World J Gastroenterol. 2014;20(21):6523-33. doi: 10.3748/wjg.v20.i21.6523	China
247	2014	Kao YH, Lin YC, Tsai MS, Sun CK, Yuan SS, Chang CY, Jawan B, Lee PH. Involvement of the nuclear high mobility group B1 peptides released from injured hepatocytes in murine hepatic fibrogenesis. Biochim Biophys Acta 2014;1842(9):1720-32. doi: 10.1016/j.bbadis.2014.06.017	Taiwan
248	2014	Zhang LJ, Sun MY, Ning BB, Zhang WM, Chen GF, Mu YP, Zhang H, Liu J, Bian YQ, Liu P. Xiayuxue Decoction ([symbols; see text]) attenuates hepatic stellate cell activation and sinusoidal endothelium defenestration in CCl4-induced fibrotic liver of	China

		mice. Chin J Integr Med. 2014;20(7):516-23. doi: 10.1007/s11655-014-1862-y	
249	2014	Qin W, Deng CL, Chen W, Gan Y. [Effects of lingqijuangan capsule medicated serum on apoptosis of activated rat hepatic stellate cells]. Sichuan Da Xue Xue Bao Yi Xue Ban 2014;45(4):595-600. Chinese. PMID: 25286683	China
250	2014	Fu MY, He YJ, Lv X, Liu ZH, Shen Y, Ye GR, Deng YM, Shu JC. Transforming growth factor-β1 reduces apoptosis via autophagy activation in hepatic stellate cells. Mol Med Rep. 2014;10(3):1282-8. doi: 10.3892/mmr.2014.2383	China
251	2014	Huang D, Jiang Y, Chen W, Yao F, Sun L. Polyphenols with anti-proliferative activities from Penthorum chinense Pursh. Molecules 2014;19(8):11045-55. doi: 10.3390/molecules190811045	China
252	2014	Ding X, Chen X, Cao Y, Jia D, Wang D, Zhu Z, Zhang J, Hong Z, Chai Y. Quality improvements of cell membrane chromatographic column. J Chromatogr A. 2014;1359:330-5. doi: 10.1016/j.chroma.2014.07.071	China
253	2014	Yang MH, Kim NH, Heo JD, Sung SH, Jeong EJ. Hepatoprotective effects of Limonium tetragonum, edible medicinal halophyte growing near seashores. Pharmacogn Mag. 2014;10(Suppl 3):S563-8. doi: 10.4103/0973-1296.139783	Korea
254	2014	Fang L, Huang C, Meng X, Wu B, Ma T, Liu X, Zhu Q, Zhan S, Li J. TGF-β1-elevated TRPM7 channel regulates collagen expression in hepatic stellate cells via TGF-β1/Smad pathway. Toxicol Appl Pharmacol. 2014;280(2):335-44. doi: 10.1016/j.taap.2014.08.006	China
255	2014	Chen G, Wang Y, Li M, Xu T, Wang X, Hong B, Niu Y. Curcumol induces HSC-T6 cell death through suppression of Bcl-2: involvement of PI3K and NF-κB pathways. Eur J Pharm Sci. 2014;65:21-8. doi: 10.1016/j.ejps.2014.09.001	China
256	2014	Dai E, Zhang J, Zhang D, Yang L, Wang Y, Jiang X, Ye L, Li X, Liu H, Ma J, Jiang H. Rimonabant inhibits proliferation, collagen secretion and induces apoptosis in hepatic stellate cells. Hepatogastroenterology 2014;61(135):2052-61. PMID: 25713910	China
257	2014	Liu TT, Ding TL, Ma Y, Wei W. Selective α1B- and α1D-adrenoceptor antagonists suppress noradrenaline-induced activation, proliferation and ECM secretion of rat hepatic stellate cells in vitro. Acta Pharmacol Sin. 2014;35(11):1385-92. doi: 10.1038/aps.2014.84	China
258	2014	Jo YH, Shin B, Liu Q, Lee KY, Oh DC, Hwang BY, Lee MK. Antiproliferative prenylated xanthones and benzophenones from the roots of Cudrania tricuspidata in HSC-T6 cells. J Nat Prod. 2014;77(11):2361-6. doi: 10.1021/np5002797	Korea
259	2014	Zhao Y, Ma X, Wang J, Zhu Y, Li R, Wang J, He X, Shan L, Wang R, Wang L, Li Y, Xiao X. Paeoniflorin alleviates liver fibrosis by inhibiting HIF-1α through mTOR-dependent pathway. Fitoterapia 2014;99:318-27. doi: 10.1016/j.fitote.2014.10.009	China
260	2014	Jiang Y, Wang C, Li YY, Wang XC, An JD, Wang YJ, Wang XJ. Mistletoe alkaloid fractions alleviates carbon tetrachloride-induced liver fibrosis through inhibition of hepatic stellate cell activation via TGF-β/Smad interference. J Ethnopharmacol.	China

		2014;158 Pt A:230-8. doi: 10.1016/j.jep.2014.10.028	
261	2014	Mai ZP, Wang C, Wang Y, Zhang HL, Zhang BJ, Wang W, Huo XK, Huang SS, Wang CY, Liu KX, Ma XC, Wang XB. Bioactive metabolites of Schisanlactone E transformed by *Cunninghamella blakesleana* AS 3.970. Fitoterapia 2014;99:352-61. doi: 10.1016/j.fitote.2014.10.016	China
262	2014	Kim HG, Kim JM, Han JM, Lee JS, Choi MK, Lee DS, Park YH, Son CG. Chunggan extract, a traditional herbal formula, ameliorated alcohol-induced hepatic injury in rat model. World J Gastroenterol. 2014;20(42):15703-14. doi: 10.3748/wjg.v20.i42.15703	Korea
263	2015	Li J, Li X, Xu W, Wang S, Hu Z, Zhang Q, Deng X, Wang J, Zhang J, Guo C. Antifibrotic effects of luteolin on hepatic stellate cells and liver fibrosis by targeting AKT/mTOR/p70S6K and TGFβ/Smad signalling pathways. Liver Int. 2015;35(4):1222-33. doi: 10.1111/liv.12638	China
264	2015	Xu T, Pan Z, Dong M, Yu C, Niu Y. Ferulic acid suppresses activation of hepatic stellate cells through ERK1/2 and Smad signaling pathways in vitro. Biochem Pharmacol. 2015;93(1):49-58. doi: 10.1016/j.bcp.2014.10.016	China
265	2015	Luo J, Zhang C, Zhu H, Jin X, Cao S, Jin M, Jiang Z, Zheng M, Li G. A new chromene from the fruiting bodies of Chroogomphus rutilus. Nat Prod Res. 2015;29(8):698-702. doi: 10.1080/14786419.2014.981813	China
266	2015	Zhang DS, Li YY, Chen XJ, Li YJ, Liu ZY, Xie WJ, Sun ZL. BCL2 promotor methylation and miR-15a/16-1 upregulation is associated with sanguinarine- induced apoptotic death in rat HSC-T6 cells. J Pharmacol Sci. 2015;127(1):135-44. doi: 10.1016/j.jphs.2014.11.012	China
267	2015	Jeong EJ, Kim NH, Heo JD, Lee KY, Rho JR, Kim YC, Sung SH. Antifibrotic compounds from Liriodendron tulipifera attenuating HSC-T6 proliferation and TNF-*α* production in RAW264.7 cells. Biol Pharm Bull. 2015;38(2):228-34. doi: 10.1248/bpb.b14-00583	Korea
268	2015	Cheng J, Wang M, Ma H, Li H, Ren J, Wang R. [Adiponectin inhibits oxidative stress and modulates TGF-β1 and COL-1 expression via the AMPK pathway in HSC-T6 cells]. Zhonghua Gan Zang Bing Za Zhi. 2015;23(1):69-72. Chinese. doi: 10.3760/cma.j.issn.1007-3418.2015.01.016	China
269	2015	Xu Y, Peng Z, Ji W, Li X, Lin X, Qian L, Li X, Chai X, Wu Q, Gao Q, Su C. A novel matrine ferivative WM130 inhibits activation of hepatic stellate cells and attenuates dimethylnitrosamine-induced liver fibrosis in rats. Biomed Res Int. 2015;2015:203978. doi: 10.1155/2015/203978	China
270	2015	Yang MF, Li DF, Nie YQ. Research of inhibition of survivin in rat HSC-T6 cell by siRNA interference. Bratisl Lek Listy 2015;116(7):446-50. doi: 10.4149/bll_2015_084	China

271	2015	Stone LC, Thorne LS, Weston CJ, Graham M, Hodges NJ. Cytoglobin expression in the hepatic stellate cell line HSC-T6 is regulated by extracellular matrix proteins dependent on FAK-signalling. Fibrogenesis Tissue Repair 2015;8:15. doi: 10.1186/s13069-015-0032-y	UK
272	2015	He W, Shi F, Zhou ZW, Li B, Zhang K, Zhang X, Ouyang C, Zhou SF, Zhu X. A bioinformatic and mechanistic study elicits the antifibrotic effect of ursolic acid through the attenuation of oxidative stress with the involvement of ERK, PI3K/Akt, and p38 MAPK signaling pathways in human hepatic stellate cells and rat liver. Drug Des Devel Ther. 2015;9:3989-4104. doi: 10.2147/DDDT.S85426	China / USA
273	2015	Hu J, Cao G, Wu X, Cai H, Cai B. Tetramethylpyrazine inhibits activation of hepatic stellate cells through Hedgehog signaling pathways in vitro. Biomed Res Int. 2015;2015:603067. doi: 10.1155/2015/603067	China
274	2015	Chen S, Chen Y, Chen B, Cai YJ, Zou ZL, Wang JG, Lin Z, Wang XD, Fu LY, Hu YR, Chen YP, Chen DZ. Plumbagin ameliorates CCl4-induced hepatic fibrosis in rats via the epidermal growth factor receptor signaling pathway. Evid Based Complement Alternat Med. 2015;2015:645727. doi: 10.1155/2015/645727	China
275	2015	Tao LL, Zhai YZ, Ding D, Yin WH, Liu XP, Yu GY. The role of C/EBP-α expression in human liver and liver fibrosis and its relationship with autophagy. Int J Clin Exp Pathol. 2015;8(10):13102-7. PMID: 26722507	China
276	2015	Xie WL, Jiang R, Shen XL, Chen ZY, Deng XM. Diosgenin attenuates hepatic stellate cell activation through transforming growth factor-β/Smad signaling pathway. Int J Clin Exp Med. 2015;8(11):20323-9. PMID: 26884947	China
277	2015	Li J, Dong N, Cheng S, Li X, Wang W, Xiang Y. Tetramethylpyrazine inhibits CTGF and Smad2/3 expression and proliferation of hepatic stellate cells. Biotechnol Biotechnol Equip. 2015;29(1):124-31. doi: 10.1080/13102818.2014.984382	China
278	2015	Lee PJ, Woo SJ, Jee JG, Sung SH, Kim HP. Bisdemethoxycurcumin Induces apoptosis in activated hepatic stellate cells via cannabinoid receptor 2. Molecules 2015;20(1):1277-92. doi: 10.3390/molecules20011277	Korea
279	2015	Zhan L, Yang Y, Ma TT, Huang C, Meng XM, Zhang L, Li J. Transient receptor potential vanilloid 4 inhibits rat HSC-T6 apoptosis through induction of autophagy. Mol Cell Biochem. 2015;402(1-2):9-22. doi: 10.1007/s11010-014-2298-6	China
280	2015	Zhang K, Zhang YQ, Ai WB, Hu QT, Zhang QJ, Wan LY, Wang XL, Liu CB, Wu JF. Hes1, an important gene for activation of hepatic stellate cells, is regulated by Notch1 and TGF-β/BMP signaling. World J Gastroenterol. 2015;21(3):878-87. doi: 10.3748/wjg.v21.i3.878	China
281	2015	Chong LW, Hsu YC, Lee TF, Lin Y, Chiu YT, Yang KC, Wu JC, Huang YT. Fluvastatin attenuates hepatic steatosis-induced fibrogenesis in rats through inhibiting paracrine effect of hepatocyte on hepatic stellate cells. BMC Gastroenterol. 2015;15:22. doi: 10.1186/s12876-015-0248-8	Taiwan

282	2015	Lu CH, Hou QR, Deng LF, Fei C, Xu WP, Zhang Q, Wu KM, Ning BF, Xie WF, Zhang X. MicroRNA-370 attenuates hepatic fibrogenesis by targeting smoothened. Dig Dis Sci. 2015;60(7):2038-48. doi: 10.1007/s10620-015-3585-0	China
283	2015	Lin X, Kong LN, Huang C, Ma TT, Meng XM, He Y, Wang QQ, Li J. Hesperetin derivative-7 inhibits PDGF-BB-induced hepatic stellate cell activation and proliferation by targeting Wnt/β-catenin pathway. Int Immunopharmacol. 2015;25(2):311-20. doi: 10.1016/j.intimp.2015.02.009	China
284	2015	Xiang XH, Jiang TP, Zhang S, Song J, Li X, Yang JY, Zhou S. Pirfenidone inhibits proliferation, arrests the cell cycle, and downregulates heat shock protein-47 and collagen type I in rat hepatic stellate cells in vitro. Mol Med Rep. 2015;12(1):309-14. doi: 10.3892/mmr.2015.3403	China
285	2015	Wang J, Chu ES, Chen HY, Man K, Go MY, Huang XR, Lan HY, Sung JJ, Yu J. microRNA-29b prevents liver fibrosis by attenuating hepatic stellate cell activation and inducing apoptosis through targeting PI3K/AKT pathway. Oncotarget 2015;6(9):7325-38. doi: 10.18632/oncotarget.2621	China
286	2015	Ren B, Fan HN, Deng Y, Wang HJ, Ren L. [Effect of Echinococcus multilocularis Cyst Fluid on the Expression of Five MAPK-pathway Genes of Rat Hepatic Stellate Cells]. Zhongguo Ji Sheng Chong Xue Yu Ji Sheng Chong Bing Za Zhi 2015;33(2):114-7, 121. Chinese. PMID: 26245121	China
287	2015	Zhang X, Han X, Yin L, Xu L, Qi Y, Xu Y, Sun H, Lin Y, Liu K, Peng J. Potent effects of dioscin against liver fibrosis. Sci Rep. 2015;5:9713. doi: 10.1038/srep09713	China
288	2015	Chen Z, Jin W, Liu H, Zhao Z, Cheng K. Discovery of Peptide ligands for hepatic stellate cells using phage display. Mol Pharm. 2015;12(6):2180-8. doi: 10.1021/acs.molpharmaceut.5b00177	USA
289	2015	Liu K, Guo MG, Lou XL, Li XY, Xu Y, Ji WD, Huang XD, Yang JH, Duan JC. Hepatocyte nuclear factor 4α induces a tendency of differentiation and activation of rat hepatic stellate cells. World J Gastroenterol. 2015;21(19):5856-66. doi: 10.3748/wjg.v21.i19.5856	China
290	2015	He Y, Zhu J, Huang Y, Gao H, Zhao Y. Advanced glycation end product (AGE)-induced hepatic stellate cell activation via autophagy contributes to hepatitis C-related fibrosis. Acta Diabetol. 2015;52(5):959-69. doi: 10.1007/s00592-015-0763-7	China
291	2015	Li Y, Liu F, Ding F, Chen P, Tang M. Inhibition of liver fibrosis using vitamin A-coupled liposomes to deliver matrix metalloproteinase-2 siRNA in vitro. Mol Med Rep. 2015;12(3):3453-61. doi: 10.3892/mmr.2015.3842	China
292	2015	Huang H, Zhou J, Cui Z, Wang B, Hu Y. Angiotensin II type 1 receptor- associated protein plays a role in regulating the local renin-angiotensin system in HSC-T6 cells. Mol Med Rep. 2015;12(3):3763-68. doi: 10.3892/mmr.2015.3849	China

293	2015	Anavi S, Eisenberg-Bord M, Hahn-Obercyger M, Genin O, Pines M, Tirosh O. The role of iNOS in cholesterol-induced liver fibrosis. Lab Invest. 2015;95(8):914-24. doi: 10.1038/labinvest.2015.67	Israel
294	2015	Wang Q, Wen R, Lin Q, Wang N, Lu P, Zhu X. Wogonoside shows antifibrotic effects in an experimental regression model of hepatic fibrosis. Dig Dis Sci. 2015;60(11):3329-39. doi: 10.1007/s10620-015-3751-4	China
295	2015	Wu FR, Jiang L, He XL, Zhu PL, Li J. Effect of hesperidin on TGF-beta1/Smad signaling pathway in HSC. Zhongguo Zhong Yao Za Zhi. 2015;40(13):2639-43. PMID: 26697692	China
296	2015	Liu YW, Chiu YT, Fu SL, Huang YT. Osthole ameliorates hepatic fibrosis and inhibits hepatic stellate cell activation. J Biomed Sci. 2015;22(1):63. doi: 10.1186/s12929-015-0168-5	Taiwan
297	2015	Lin YC, Luo HY, Jin QX. [Study on effect of total flavanones of Sedum sarmentosum on apoptosis of hepatic stellate cells and its mechanism]. Zhongguo Zhong Yao Za Zhi 2015;40(16):3273-7. Chinese. PMID: 26790306	China
298	2015	Bohanon FJ, Wang X, Graham BM, Ding C, Ding Y, Radhakrishnan GL, Rastellini C, Zhou J, Radhakrishnan RS. Enhanced effects of novel oridonin analog CYD0682 for hepatic fibrosis. J Surg Res. 2015;199(2):441-9. doi: 10.1016/j.jss.2015.07.042	USA
299	2015	Lu L, Wang J, Lu H, Zhang G, Liu Y, Wang J, Zhang Y, Shang H, Ji H, Chen X, Duan Y, Li Y. MicroRNA-130a and -130b enhance activation of hepatic stellate cells by suppressing PPARγ expression: A rat fibrosis model study. Biochem Biophys Res Commun. 2015;465(3):387-93. doi: 10.1016/j.bbrc.2015.08.012	China
300	2015	Liu LH, Lai QN, Chen JY, Zhang JX, Cheng B. Overexpression of pim-3 and protective role in lipopolysaccharide-stimulated hepatic stellate cells. World J Gastroenterol. 2015;21(29):8858-67. doi: 10.3748/wjg.v21.i29.8858	China
301	2015	Wang Y, Zhang X, Yang Y, Yang X, Ye B. Study on the antifibrotic effects of recombinant shark hepatical stimulator analogue (r-sHSA) in vitro and in vivo. Mar Drugs 2015;13(8):5201-18. doi: 10.3390/md13085201	China
302	2015	Lu S, Cheng M, Yang D, Liu Y, Guan L, Wu J. [Effects of blueberry on apoptosis and expression of Bcl-2 and Bax in HSC-T6]. Zhonghua Yi Xue Za Zhi 2015;95(31):2560-4. Chinese. PMID: 26711393	China
303	2015	Eichmann TO, Grumet L, Taschler U, Hartler J, Heier C, Woblistin A, Pajed L, Kollroser M, Rechberger G, Thallinger GG, Zechner R, Haemmerle G, Zimmermann R, Lass A. ATGL and CGI-58 are lipid droplet proteins of the hepatic stellate cell line HSC-T6. J Lipid Res. 2015;56(10):1972-84. doi: 10.1194/jlr.M062372	Austria
304	2015	Bohanon FJ, Wang X, Graham BM, Prasai A, Vasudevan SJ, Ding C, Ding Y, Radhakrishnan GL, Rastellini C, Zhou J, Radhakrishnan RS. Enhanced anti- fibrogenic effects of novel oridonin derivative CYD0692 in hepatic stellate cells. Mol Cell Biochem. 2015;410(1-2):293-300. doi: 10.1007/s11010-015-2562-4	USA

305	2015	Choi JS, Kim JK, Yang YJ, Kim Y, Kim P, Park SG, Cho EY, Lee DH, Choi JW. Identification of cromolyn sodium as an anti-fibrotic agent targeting both hepatocytes and hepatic stellate cells. Pharmacol Res. 2015;102:176-83. doi: 10.1016/j.phrs.2015.10.002	Korea
306	2015	He YJ, Kuchta K, Lv X, Lin Y, Ye GR, Liu XY, Song HD, Wang LX, Kobayashi Y, Shu JC. Curcumin, the main active constituent of turmeric (Curcuma longa L.), induces apoptosis in hepatic stellate cells by modulating the abundance of apoptosis-related growth factors. Z Naturforsch C J Biosci. 2015;70(11-12):281-5. doi: 10.1515/znc-2015-4143	China / Japan
307	2015	You SP, Zhao J, Ma L, Tudimat M, Zhang SL, Liu T. Preventive effects of phenylethanol glycosides from Cistanche tubulosa on bovine serum albumin-induced hepatic fibrosis in rats. Daru 2015;23:52. doi: 10.1186/s40199-015-0135-4	China
308	2015	Liu M, Xu Y, Han X, Yin L, Xu L, Qi Y, Zhao Y, Liu K, Peng J. Dioscin alleviates alcoholic liver fibrosis by attenuating hepatic stellate cell activation via the TLR4/MyD88/NF-κB signaling pathway. Sci Rep. 2015;5:18038. doi: 10.1038/srep18038. Erratum in: Sci Rep. 2020;10(1):18384. PMID: 26655640	China
309	2016	Chen J, Liu DG, Wang H, Wu XN, Cong M, You H, Jia JD. NIM811 downregulates transforming growth factor-β signal transduction in vivo and in vitro. Mol Med Rep. 2016;13(1):522-8. doi: 10.3892/mmr.2015.4572	China
310	2016	Liu Z, Dou W, Zheng Y, Wen Q, Qin M, Wang X, Tang H, Zhang R, Lv D, Wang J, Zhao S. Curcumin upregulates Nrf2 nuclear translocation and protects rat hepatic stellate cells against oxidative stress. Mol Med Rep. 2016;13(2):1717-24. doi: 10.3892/mmr.2015.4690	China
311	2016	Chen PJ, Cai SP, Yang Y, Li WX, Huang C, Meng XM, Li J. PTP1B confers liver fibrosis by regulating the activation of hepatic stellate cells. Toxicol Appl Pharmacol. 2016;292:8-18. doi: 10.1016/j.taap.2015.12.021	China
312	2016	Gu L, Tao X, Xu Y, Han X, Qi Y, Xu L, Yin L, Peng J. Dioscin alleviates BDL- and DMN-induced hepatic fibrosis via Sirt1/Nrf2-mediated inhibition of p38 MAPK pathway. Toxicol Appl Pharmacol. 2016;292:19-29. doi: 10.1016/j.taap.2015.12.024. Erratum in: Toxicol Appl Pharmacol. 2019;380:114708. PMID: 26747300	China
313	2016	Jia D, Ni YR, Zhang YQ, Rao C, Hou J, Tang HQ, Liu CB, Wu JF. SP1 and UTE1 Decoy ODNs inhibit activation and proliferation of hepatic stellate cells by targeting tissue inhibitors of metalloproteinase 1. Cell Biosci. 2016;6:31. doi: 10.1186/s13578-016-0094-6	China
314	2016	Su M, Chao G, Liang M, Song J, Wu K. Anticytoproliferative effect of Vitamin C on rat hepatic stellate cell. Am J Transl Res. 2016;8(6):2820-5. PMID: 27398165	China
315	2016	Peterová E, Podmolíková L, Řezáčová M, Mrkvicová A. Fibroblast growth factor-1 suppresses TGF-β-mediated myofibroblastic differentiation of rat hepatic stellate cells. Acta Medica (Hradec Kralove) 2016;59(4):124-32. doi: 10.14712/18059694.2017.39	Czech Republic

316	2016	Yu F, Lu Z, Huang K, Wang X, Xu Z, Chen B, Dong P, Zheng J. MicroRNA-17-5p-activated Wnt/β-catenin pathway contributes to the progression of liver fibrosis. Oncotarget 2016;7(1):81-93. doi: 10.18632/oncotarget.6447	China
317	2016	Wang H, Liu S, Wang Y, Chang B, Wang B. Nod-like receptor protein 3 inflammasome activation by Escherichia coli RNA induces transforming growth factor beta 1 secretion in hepatic stellate cells. Bosn J Basic Med Sci. 2016;16(2):126-31. doi: 10.17305/bjbms.2016.699	China
318	2016	Zhang X, Zhang J, Jia L, Xiao S. Dicliptera Chinensis polysaccharides target TGF-β/Smad pathway and inhibit stellate cells activation in rats with dimethylnitrosamine-induced hepatic fibrosis. Cell Mol Biol. (Noisy-le-grand) 2016;62(1):99-103. PMID: 26828995	China
319	2016	Chen L, Guo YZ, Li AD, Ma JJ, Hao HY, Zhang D, Wang Y, Ji CG, Qi W, Wang J, Jiang HQ. Knockdown of astrocyte elevated gene-1 inhibits activation of hepatic stellate cells. Dig Dis Sci. 2016;61(7):1961-71. doi: 10.1007/s10620-016-4075-8	China
320	2016	Zhou D, He LN, Wang J, Ding YN, Chen YW, Fan JG. [Binding characteristics of chemosynthetic Ac-SDKP analogue FAM-Aca-SDKP to hepatic stellate cell-T6 cells]. Zhonghua Gan Zang Bing Za Zhi 2016;24(3):186-90. Chinese. doi: 10.3760/cma.j.issn.1007-3418.2016.03.006	China
321	2016	Yuan J, Liu YY, Ma XJ, Li JB. [Effect of high fat on fibrosis in rat hepatic stellate cells]. Zhonghua Gan Zang Bing Za Zhi 2016;24(3):191-5. Chinese. doi: 10.3760/cma.j.issn.1007-3418.2016.03.007	China
322	2016	Tai CJ, Choong CY, Lin YC, Shi YC, Tai CJ. The anti-hepatic fibrosis activity of ergosterol depended on upregulation of PPARgamma in HSC-T6 cells. Food Funct. 2016;7(4):1915-23. doi: 10.1039/c6fo00117c	Taiwan
323	2016	Wu P, Huang R, Xiong YL, Wu C. Protective effects of curcumin against liver fibrosis through modulating DNA methylation. Chin J Nat Med. 2016;14(4):255-64. doi: 10.1016/S1875-5364(16)30025-5	China
324	2016	Jia Y, Yuan L, Xu T, Li H, Yang G, Jiang M, Zhang C, Li C. Herbal medicine Gan-fu-kang downregulates Wnt/Ca2+ signaling to attenuate liver fibrogenesis in vitro and in vivo. Mol Med Rep. 2016;13(6):4705-14. doi: 10.3892/mmr.2016.5148	China
325	2016	Zhang Y, Zhao X, Chang Y, Zhang Y, Chu X, Zhang X, Liu Z, Guo H, Wang N, Gao Y, Zhang J, Chu L. Calcium channel blockers ameliorate iron overload- associated hepatic fibrosis by altering iron transport and stellate cell apoptosis. Toxicol Appl Pharmacol. 2016;301:50-60. doi: 10.1016/j.taap.2016.04.008	China
326	2016	He YH, Li Z, Ni MM, Zhang XY, Li MF, Meng XM, Huang C, Li J. Cryptolepine derivative-6h inhibits liver fibrosis in TGF-β1-induced HSC-T6 cells by targeting the Shh pathway. Can J Physiol Pharmacol. 2016;94(9):987-95. doi: 10.1139/cjpp-2016-0157	China
327	2016	Li X, Jin Q, Wu YL, Sun P, Jiang S, Zhang Y, Zhang DQ, Zhang YJ, Lian LH, Nan JX. Tetrandrine regulates hepatic stellate cell	China

		activation via TAK1 and NF- κB signaling. Int Immunopharmacol. 2016;36:263-70. doi: 10.1016/j.intimp.2016.04.039	
328	2016	Yang JJ, Liu LP, Tao H, Hu W, Shi P, Deng ZY, Li J. MeCP2 silencing of LncRNA H19 controls hepatic stellate cell proliferation by targeting IGF1R. Toxicology 2016;359-360:39-46. doi: 10.1016/j.tox.2016.06.016	China
329	2016	Miyamae Y, Nishito Y, Nakai N, Nagumo Y, Usui T, Masuda S, Kambe T, Nagao M. Tetrandrine induces lipid accumulation through blockade of autophagy in a hepatic stellate cell line. Biochem Biophys Res Commun. 2016;477(1):40-6. doi: 10.1016/j.bbrc.2016.06.018	Japan
330	2016	Zhou DD, Wang X, Wang Y, Xiang XJ, Liang ZC, Zhou Y, Xu A, Bi CH, Zhang L. MicroRNA-145 inhibits hepatic stellate cell activation and proliferation by targeting ZEB2 through Wnt/β-catenin pathway. Mol Immunol. 2016;75:151-60. doi: 10.1016/j.molimm.2016.05.018	China
331	2016	Huang Q, Wei L, Liang C, Nie J, Lu S, Lu C, Tan S, Lv S, Zhuo L, Lu Z, Lin X. Loss of Raf kinase inhibitor protein is associated with malignant progression in hepatic fibrosis. Biomed Pharmacother. 2016;82:669-76. doi: 10.1016/j.biopha.2016.06.007	China
332	2016	Feng Y, Ying HY, Qu Y, Cai XB, Xu MY, Lu LG. Novel matrine derivative MD-1 attenuates hepatic fibrosis by inhibiting EGFR activation of hepatic stellate cells. Protein Cell. 2016;7(9):662-72. doi: 10.1007/s13238-016-0285-2	China
333	2016	Wu YL, Zhang YJ, Yao YL, Li ZM, Han X, Lian LH, Zhao YQ, Nan JX. Cucurbitacin E ameliorates hepatic fibrosis in vivo and in vitro through activation of AMPK and blocking mTOR-dependent signaling pathway. Toxicol Lett. 2016;258:147-58. doi: 10.1016/j.toxlet.2016.06.2102	China
334	2016	Wan LY, Zhang YQ, Li JM, Tang HQ, Chen MD, Ni YR, Huang H, Liu CB, Wu JF. Liganded vitamin D receptor through its interacting repressor inhibits the expression of type I collagen α1. DNA Cell Biol. 2016;35(9):498-505. doi: 10.1089/dna.2016.3367	China
335	2016	Lee SY, Lee J, Lee H, Kim B, Lew J, Baek N, Kim SH. MicroRNA134 mediated upregulation of JNK and downregulation of NFkB signalings are critically involved in dieckol induced antihepatic fibrosis. J Agric Food Chem. 2016;64(27):5508-14. doi: 10.1021/acs.jafc.6b01945	Korea
336	2016	Li X, Wu XQ, Xu T, Li XF, Yang Y, Li WX, Huang C, Meng XM, Li J. Role of histone deacetylases(HDACs) in progression and reversal of liver fibrosis. Toxicol Appl Pharmacol. 2016;306:58-68. doi: 10.1016/j.taap.2016.07.003	China
337	2016	Twu YC, Lee TS, Lin YL, Hsu SM, Wang YH, Liao CY, Wang CK, Liang YC, Liao YJ. Niemann-Pick type C2 protein mediates hepatic stellate cells activation by regulating free cholesterol accumulation. Int J Mol Sci. 2016;17(7):1122. doi: 10.3390/ijms17071122	Taiwan
338	2016	Lee HS, Shin HS, Choi J, Bae SJ, Wee HJ, Son T, Seo JH, Park JH, Kim SW, Kim KW. AMP-activated protein kinase activator, HL156A reduces thioacetamide- induced liver fibrosis in mice and inhibits the activation of cultured hepatic stellate cells and	Korea

		macrophages. Int J Oncol. 2016;49(4):1407-14. doi: 10.3892/ijo.2016.3627	
339	2016	Chen MF, Huang SJ, Huang CC, Liu PS, Lin KI, Liu CW, Hsieh WC, Shiu LY, Chen CH. Saikosaponin d induces cell death through caspase-3-dependent, caspase-3-independent and mitochondrial pathways in mammalian hepatic stellate cells. BMC Cancer 2016;16:532. doi: 10.1186/s12885-016-2599-0	Taiwan
340	2016	Fan EY, He SQ, Wen B, Sun HT, Jia WY, Chen GX. [Effects of Biejiajian Pill on Proliferation and Apoptosis of Hepatic Stellate Cells in Mice]. Zhongguo Zhong Xi Yi Jie He Za Zhi 2016;36(8):960-6. Chinese. PMID: 30640992	China
341	2016	Liu XY, Liu RX, Hou F, Cui LJ, Li CY, Chi C, Yi E, Wen Y, Yin CH. Fibronectin expression is critical for liver fibrogenesis in vivo and in vitro. Mol Med Rep. 2016;14(4):3669-75. doi: 10.3892/mmr.2016.5673	China
342	2016	Liu Y, Yang P, Chen N, Lin S, Liu M. Effects of recombinant human adenovirus-p53 on the regression of hepatic fibrosis. Int J Mol Med. 2016;38(4):1093-100. doi: 10.3892/ijmm.2016.2716	China
343	2016	Tseng TH, Lin WL, Chen ZH, Lee YJ, Shie MS, Lee KF, Shen CH, Kuo HC. Moniliformediquinone as a potential therapeutic agent, inactivation of hepatic stellate cell and inhibition of liver fibrosis in vivo. J Transl Med. 2016;14(1):263. doi: 10.1186/s12967-016-1022-6	Taiwan
344	2016	Bai G, Yan G, Wang G, Wan P, Zhang R. Anti-hepatic fibrosis effects of a novel turtle shell decoction by inhibiting hepatic stellate cell proliferation and blocking TGF-β1/Smad signaling pathway in rats. Oncol Rep. 2016;36(5):2902-10. doi: 10.3892/or.2016.5078	China
345	2016	He L, Li Z, Zhou D, Ding Y, Xu L, Chen Y, Fan J. Galanin receptor 2 mediates antifibrogenic effects of galanin on hepatic stellate cells. Exp Ther Med. 2016;12(5):3375-80. doi: 10.3892/etm.2016.3764	China
346	2016	Zhao J, Peng L, Cui R, Guo X, Yan M. Dimethyl α-ketoglutarate reduces CCl4-induced liver fibrosis through inhibition of autophagy in hepatic stellate cells. Biochem Biophys Res Commun. 2016;481(1-2):90-6. doi: 10.1016/j.bbrc.2016.11.010	China
347	2016	Cai SP, Cheng XY, Chen PJ, Pan XY, Xu T, Huang C, Meng XM, Li J. Transmembrane protein 88 attenuates liver fibrosis by promoting apoptosis and reversion of activated hepatic stellate cells. Mol Immunol. 2016;80:58-67. doi: 10.1016/j.molimm.2016.11.002	China
348	2016	Bi S, Chu F, Wang M, Li B, Mao P, Zhang H, Wang P, Guo W, Xu L, Ren L, Lei H, Zhang Y. Ligustrazine-oleanolic acid glycine derivative, G-TOA, selectively inhibited the proliferation and induced apoptosis of activated HSC-T6 cells. Molecules 2016;21(11):1599. doi: 10.3390/molecules21111599	China
349	2016	Cheng CF, Pan TM. Ankaflavin and Monascin Induce Apoptosis in activated hepatic stellate cells through suppression of the	Taiwan

		Akt/NF-κB/p38 signaling pathway. J Agric Food Chem. 2016;64(49):9326-34. doi: 10.1021/acs.jafc.6b03700	
350	2017	Li WX, Chen X, Yang Y, Huang HM, Li HD, Huang C, Meng XM, Li J. Hesperitin derivative-11 suppress hepatic stellate cell activation and proliferation by targeting PTEN/AKT pathway. Toxicology 2017;381:75-86. doi: 10.1016/j.tox.2016.11.004	China
351	2017	351: Wu Y, Bu F, Yu H, Li W, Huang C, Meng X, Zhang L, Ma T, Li J. Methylation of Septin9 mediated by DNMT3a enhances hepatic stellate cells activation and liver fibrogenesis. Toxicol Appl Pharmacol. 2017;315:35-49. doi: 10.1016/j.taap.2016.12.002	China
352	2017	Chen G, Li J, Yan S, Lin H, Wu J, Zhai X, Song Y, Li J. Biotransformation of 20(R)-panaxatriol by Mucor racemosus and the anti-hepatic fibrosis activity of some products. Nat Prod Res. 2017;31(16):1880-5. doi: 10.1080/14786419.2016.1263850	China
353	2017	Chen CH, Chen MF, Huang SJ, Huang CY, Wang HK, Hsieh WC, Huang CH, Liu LF, Shiu LY. Saikosaponin A induces apoptosis through mitochondria-dependent pathway in hepatic stellate cells. Am J Chin Med. 2017;45(2):351-68. doi: 10.1142/S0192415X17500227	Taiwan
354	2017	Li B, Cong M, Zhu Y, Xiong Y, Jin W, Wan Y, Zhou Y, Ao Y, Wang H. Indole-3-carbinol induces apoptosis of hepatic stellate cells through K63 de-ubiquitination of RIP1 in rats. Cell Physiol Biochem. 2017;41(4):1481-90. doi: 10.1159/000470650	China
355	2017	Liu J, Li Y, Liu L, Wang Z, Shi C, Cheng Z, Zhang X, Ding F, Chen PS. Double knockdown of PHD1 and Keap1 attenuated hypoxia-induced injuries in hepatocytes. Front Physiol. 2017;8:291. doi: 10.3389/fphys.2017.00291	China
356	2017	Chen Q, Zhang H, Cao Y, Li Y, Sun S, Zhang J, Zhang G. Schisandrin B attenuates CCl4-induced liver fibrosis in rats by regulation of Nrf2-ARE and TGF-β/Smad signaling pathways. Drug Des Devel Ther. 2017;11:2179-91. doi: 10.2147/DDDT.S137507	China
357	2017	Chen Z, Liu H, Jain A, Zhang L, Liu C, Cheng K. Discovery of aptamer ligands for hepatic stellate cells using SELEX. Theranostics 2017;7(12):2982-95. doi: 10.7150/thno.19374	USA
358	2017	Song LY, Ma YT, Wu CF, Wang CJ, Fang WJ, Liu SK. MicroRNA-195 activates hepatic stellate cells in vitro by targeting Smad7. Biomed Res Int. 2017;2017:1945631. doi: 10.1155/2017/1945631	China
359	2017	Yin L, Qi Y, Xu Y, Xu L, Han X, Tao X, Song S, Peng J. Dioscin inhibits HSC-T6 cell migration via adjusting SDC-4 expression: Insights from iTRAQ-based quantitative proteomics. Front Pharmacol. 2017;8:665. doi: 10.3389/fphar.2017.00665. Erratum in: Front Pharmacol. 2019;10:1036. PMID: 29033837	China
360	2017	Bai F, Huang Q, Nie J, Lu S, Lu C, Zhu X, Wang Y, Zhuo L, Lu Z, Lin X. Trolline ameliorates liver fibrosis by inhibiting the NF-κB pathway, promoting HSC apoptosis and suppressing autophagy. Cell Physiol Biochem. 2017;44(2):436-46. doi: 10.1159/000485009	China

361	2017	Wang YH, Li RK, Fu Y, Li J, Yang XM, Zhang YL, Zhu L, Yang Q, Gu JR, Xing X, Zhang ZG. Exemestane attenuates hepatic fibrosis in rats by inhibiting activation of hepatic stellate cells and promoting the secretion of interleukin 10. J Immunol Res. 2017;2017:3072745. doi: 10.1155/2017/3072745	China
362	2017	Zhou D, Wang J, He LN, Li BH, Ding YN, Chen YW, Fan JG. Prolyl oligopeptidase attenuates hepatic stellate cell activation through induction of Smad7 and PPAR-γ. Exp Ther Med. 2017;13(2):780-6. doi: 10.3892/etm.2017.4033	China
363	2017	Chang YC, Hwang TL, Kuo LM, Sung PJ. Pinnisterols D-J, New 11-Acetoxy-9,11-secosterols with a 1,4-Quinone Moiety from Formosan Gorgonian Coral Pinnigorgia sp. (Gorgoniidae). Mar Drugs 2017;15(1):11. doi: 10.3390/md15010011	Taiwan
364	2017	Jain A, Barve A, Zhao Z, Jin W, Cheng K. Comparison of Avidin, Neutravidin, and streptavidin as nanocarriers for efficient siRNA delivery. Mol Pharm. 2017;14(5):1517-27. doi: 10.1021/acs.molpharmaceut.6b00933	USA
365	2017	Chen XJ, Liu WJ, Wen ML, Liang H, Wu SM, Zhu YZ, Zhao JY, Dong XQ, Li MG, Bian L, Zou CG, Ma LQ. Ameliorative effects of Compound K and ginsenoside Rh1 on non-alcoholic fatty liver disease in rats. Sci Rep. 2017;7:41144. doi: 10.1038/srep41144	China
366	2017	Lu C, Zou Y, Liu Y, Niu Y. Rosmarinic acid counteracts activation of hepatic stellate cells via inhibiting the ROS-dependent MMP-2 activity: Involvement of Nrf2 antioxidant system. Toxicol Appl Pharmacol. 2017;318:69-78. doi: 10.1016/j.taap.2017.01.008	China
367	2017	Ni YH, Huo LJ, Li TT. [Effect of interleukin-22 on proliferation and activation of hepatic stellate cells induced by acetaldehyde and related mechanism]. Zhonghua Gan Zang Bing Za Zhi 2017;25(1):9-14. Chinese. doi: 10.3760/cma.j.issn.1007-3418.2017.01.004	China
368	2017	Hao LS, Liu YL, Zhang GL, Chen J, Song XJ, Wang YL, Wang J, Jin LM. [Effects of wild-type PTEN overexpression and its mutation on F-actin in activated hepatic stellate cells]. Zhonghua Gan Zang Bing Za Zhi 2017;25(1):21-6. Chinese. doi: 10.3760/cma.j.issn.1007-3418.2017.01.006	China
369	2017	Cipriani S, Carino A, Masullo D, Zampella A, Distrutti E, Fiorucci S. Decoding the role of the nuclear receptor SHP in regulating hepatic stellate cells and liver fibrogenesis. Sci Rep. 2017;7:41055. doi: 10.1038/srep41055	Italy
370	2017	Yang N, Dang S, Shi J, Wu F, Li M, Zhang X, Li Y, Jia X, Zhai S. Caffeic acid phenethyl ester attenuates liver fibrosis via inhibition of TGF-β1/Smad3 pathway and induction of autophagy pathway. Biochem Biophys Res Commun. 2017;486(1):22-8. doi: 10.1016/j.bbrc.2017.02.057	China
371	2017	Yang N, Shi JJ, Wu FP, Li M, Zhang X, Li YP, Zhai S, Jia XL, Dang SS. Caffeic acid phenethyl ester up-regulates antioxidant levels in hepatic stellate cell line T6 via an Nrf2-mediated mitogen activated protein kinases pathway. World J Gastroenterol. 2017;23(7):1203-14. doi: 10.3748/wjg.v23.i7.1203	China

372	2017	Kang KB, Kim HW, Kim JW, Oh WK, Kim J, Sung SH. Catechin-Bound Ceanothane- Type Triterpenoid Derivatives from the Roots of Zizyphus jujuba. J Nat Prod. 2017;80(4):1048-54. doi: 10.1021/acs.jnatprod.6b01103	Korea
373	2017	Kim JY, An HJ, Kim WH, Park YY, Park KD, Park KK. Apamin suppresses biliary fibrosis and activation of hepatic stellate cells. Int J Mol Med. 2017;39(5):1188-94. doi: 10.3892/ijmm.2017.2922	Korea
374	2017	Cheng C, Yu S, Kong R, Yuan Q, Ma Y, Yang W, Cao G, Xie L. CTRP3 attenuates hepatic stellate cell activation through transforming growth factor-β/Smad signaling pathway. Biomed Pharmacother. 2017;89:1387-91. doi: 10.1016/j.biopha.2017.03.021	China
375	2017	Hao LS, Zhang JQ, Liu B, Zhang GL, Chen J, Wang YL, Zhang MT, Zhang PL. [Influence of PTEN down-regulation by in vitro RNA interference on the migration of HSC-T6 cell line]. Zhonghua Gan Zang Bing Za Zhi 2017;25(3):223-5. Chinese. doi: 10.3760/cma.j.issn.1007-3418.2017.03.012	China
376	2017	Ni YH, Huo LJ, Li TT. Antioxidant axis Nrf2-keap1-ARE in inhibition of alcoholic liver fibrosis by IL-22. World J Gastroenterol. 2017;23(11):2002-11. doi: 10.3748/wjg.v23.i11.2002	China
377	2017	Yang Y, Chen XX, Li WX, Wu XQ, Huang C, Xie J, Zhao YX, Meng XM, Li J. EZH2-mediated repression of Dkk1 promotes hepatic stellate cell activation and hepatic fibrosis. J Cell Mol Med. 2017;21(10):2317-28. doi: 10.1111/jcmm.13153	China
378	2017	Shi B, Shi J, Qin H. Effect of medicated serum of Curcumae Radix extract on mRNA expression of TIMP-1, MMPs-13 and aI-collagen of HSC-T6 cell. Saudi Pharm J. 2017;25(4):509-12. doi: 10.1016/j.jsps.2017.04.015	China
379	2017	El-Lakkany NM, El-Maadawy WH, Seif El-Din SH, Hammam OA, Mohamed SH, Ezzat SM, Safar MM, Saleh S. Rosmarinic acid attenuates hepatic fibrogenesis via suppression of hepatic stellate cell activation/proliferation and induction of apoptosis. Asian Pac J Trop Med. 2017;10(5):444-53. doi: 10.1016/j.apjtm.2017.05.012	Egypt
380	2017	Zhang YQ, Wan LY, He XM, Ni YR, Wang C, Liu CB, Wu JF. Gremlin1 accelerates hepatic stellate cell activation through upregulation of TGF-beta expression. DNA Cell Biol. 2017;36(7):603-10. doi: 10.1089/dna.2017.3707	China
381	2017	Guo Z, Li D, Peng H, Kang J, Jiang X, Xie X, Sun D, Jiang H. Specific hepatic stellate cell-penetrating peptide targeted delivery of a KLA peptide reduces collagen accumulation by inducing apoptosis. J Drug Target 2017;25(8):715-23. doi: 10.1080/1061186X.2017.1322598	China
382	2017	Liu X, Su J, Shi Y, Guo Y, Suheryani I, Zhao S, Deng Y, Meng W, Chen Y, Sun L, Dai R. Herbal formula, Baogan Yihao (BGYH), prevented dimethylnitrosamine(DMN)-induced liver injury in rats. Drug Dev Res. 2017;78(3-4):155-63. doi: 10.1002/ddr.21388	China
383	2017	Liu H, Chen Z, Jin W, Barve A, Wan YY, Cheng K. Silencing of α-complex protein-2 reverses alcohol- and cytokine-induced	USA

		fibrogenesis in hepatic stellate cells. Liver Res. 2017;1(1):70-9. doi: 10.1016/j.livres.2017.05.003	
384	2017	Hu D, Hu Y, Xu W, Yu H, Yang N, Ni S, Fu R. miR-203 inhibits the expression of collagen-related genes and the proliferation of hepatic stellate cells through a SMAD3-dependent mechanism. Mol Med Rep. 2017;16(2):1248-54. doi: 10.3892/mmr.2017.6702	China
385	2017	Yan S, Wan LY, Ju XJ, Wu JF, Zhang L, Li M, Liu Z, Wang W, Xie R, Chu LY. K^+-responsive block copolymer micelles for targeted intracellular drug delivery. Macromol Biosci. 2017;17(9). doi: 10.1002/mabi.201700143	China
386	2017	Ge S, Xiong Y, Wu X, Xie J, Liu F, He J, Xiang T, Cheng N, Lai L, Zhong Y. Role of growth factor receptor-bound 2 in CCl4-induced hepatic fibrosis. Biomed Pharmacother. 2017;92:942-51. doi: 10.1016/j.biopha.2017.05.142	China
387	2017	Liu X, Zhao X. Scoparone attenuates hepatic stellate cell activation through inhibiting TGF-β/Smad signaling pathway. Biomed Pharmacother. 2017;93:57-61. doi: 10.1016/j.biopha.2017.06.006	China
388	2017	He YJ, Kuchta K, Deng YM, Cameron S, Lin Y, Liu XY, Ye GR, Lv X, Kobayashi Y, Shu JC. Curcumin promotes apoptosis of activated hepatic stellate cells by inhibiting protein expression of the MyD88 pathway. Planta Med. 2017;83(18):1392-6. doi: 10.1055/s-0043-113044	Japan
389	2017	Peng L, Jia X, Zhao J, Cui R, Yan M. Substance P promotes hepatic stellate cell proliferation and activation via the TGF-β1/Smad-3 signaling pathway. Toxicol Appl Pharmacol. 2017;329:293-300. doi: 10.1016/j.taap.2017.06.020	China / USA
390	2017	Zhang J, Song Y, Wang QH, Li L, Ji D, Gu W, Hao M, Lu TL, Mao CQ. [Effects of Curcumae Rhizoma/vinegar-processed Curcumae Rhizoma on immune hepatic fibrosis, proliferation of HSC-T6 and expression of α-SMA and Procollagen I]. Zhongguo Zhong Yao Za Zhi 2017;42(13):2538-45. Chinese. doi: 10.19540/j.cnki.cjcmm.20170610.001	China
391	2017	Kim JY, An HJ, Kim WH, Gwon MG, Gu H, Park YY, Park KK. Anti-fibrotic effects of synthetic oligodeoxynucleotide for TGF-β1 and Smad in an animal model of liver cirrhosis. Mol Ther Nucleic Acids 2017;8:250-63. doi: 10.1016/j.omtn.2017.06.022	Korea
392	2017	Kang KB, Jun JB, Kim JW, Kim HW, Sung SH. Ceanothane- and lupane-type triterpene esters from the roots of Hovenia dulcis and their antiproliferative activity on HSC-T6 cells. Phytochemistry 2017;142:60-7. doi: 10.1016/j.phytochem.2017.06.014	Korea
393	2017	Kao YH, Chen PH, Wu TY, Lin YC, Tsai MS, Lee PH, Tai TS, Chang HR, Sun CK. Lipopolysaccharides induce Smad2 phosphorylation through PI3K/Akt and MAPK cascades in HSC-T6 hepatic stellate cells. Life Sci. 2017;184:37-46. doi: 10.1016/j.lfs.2017.07.004	Taiwan
394	2017	Bai GP, Yan GH, Wang GJ, Wan P, Liu XF, Wang H, Zhang RH. Effects of lentiviral vector-mediated shRNA silencing of TGFβ1 on the expression of Col1a1 in rat hepatic stellate cells. Mol Med Rep. 2017;16(3):2785-90. doi: 10.3892/mmr.2017.6945	China
395	2017	Jiang M, Wu YL, Li X, Zhang Y, Xia KL, Cui BW, Lian LH, Nan JX. Oligomeric proanthocyanidin derived from grape seeds	China

		inhibited NF-κB signaling in activated HSC: Involvement of JNK/ERK MAPK and PI3K/Akt pathways. Biomed Pharmacother. 2017;93:674-80. doi: 10.1016/j.biopha.2017.06.105	
396	2017	Chen J, Yu Y, Li S, Liu Y, Zhou S, Cao S, Yin J, Li G. MicroRNA-30a ameliorates hepatic fibrosis by inhibiting Beclin1-mediated autophagy. J Cell Mol Med. 2017;21(12):3679-92. doi: 10.1111/jcmm.13278	China
397	2017	Hu Z, You P, Xiong S, Gao J, Tang Y, Ye X, Xia Y, Zhang D, Liu Y. Carapax Trionycis extracts inhibit fibrogenesis of activated hepatic stellate cells via TGF-β1/Smad and NFκB signaling. Biomed Pharmacother. 2017;95:11-7. doi: 10.1016/j.biopha.2017.08.011	China
398	2017	Liu Y, Pan X, Li S, Yu Y, Chen J, Yin J, Li G. Endoplasmic reticulum stress restrains hepatocyte growth factor expression in hepatic stellate cells and rat acute liver failure model. Chem Biol Interact. 2017;277:43-54. doi: 10.1016/j.cbi.2017.08.015	China
399	2017	Tao LL, Ding D, Yin WH, Peng JY, Hou CJ, Liu XP, Chen YL. TSA increases C/EBP-α expression by increasing its lysine acetylation in hepatic stellate cells. Mol Med Rep. 2017;16(5):6088-93. doi: 10.3892/mmr.2017.7358	China
400	2017	Koppula S, Yum MJ, Kim JS, Shin GM, Chae YJ, Yoon T, Chun CS, Lee JD, Song M. Anti-fibrotic effects of Orostachys japonicus A. Berger (Crassulaceae) on hepatic stellate cells and thioacetamide-induced fibrosis in rats. Nutr Res Pract. 2017;11(6):470-8. doi: 10.4162/nrp.2017.11.6.470	Korea
401	2017	Yum MJ, Koppula S, Kim JS, Shin GM, Chae YJ, Yoon T, Chun CS, Lee JD, Song M. Protective effects of *Ampelopsis brevipedunculata* against in vitro hepatic stellate cells system and thioacetamide-induced liver fibrosis rat model. Pharm Biol. 2017;55(1):1577-85. doi: 10.1080/13880209.2017.1311928	Korea
402	2017	Kim JS, Koppula S, Yum MJ, Shin GM, Chae YJ, Hong SM, Lee JD, Song M. Anti- fibrotic effects of Cuscuta chinensis with in vitro hepatic stellate cells and a thioacetamide-induced experimental rat model. Pharm Biol. 2017;55(1):1909-19. doi: 10.1080/13880209.2017.1340965	Korea
403	2018	Liu H, Wang X, Han R, Zuo K, Yuan X, Li Y, Zhou J, Yan L, Chu Y. Isolation and molecular cloning of hepatocyte growth factor from guinea pig (gHGF), and expression of truncated variant of gHGF with improved anti-fibrotic activity in *Escherichia coli*. Int J Biol Macromol. 2018;106:908-16. doi: 10.1016/j.ijbiomac.2017.08.087	China
404	2018	Zhao Z, Li Y, Jain A, Chen Z, Liu H, Jin W, Cheng K. Development of a peptide-modified siRNA nanocomplex for hepatic stellate cells. Nanomedicine 2018;14(1):51-61. doi: 10.1016/j.nano.2017.08.017	USA
405	2018	Wu L, Qiu W, Sun J, Wang J. SENP1 attenuates the liver fibrosis through down-regulating the expression of SMAD2. Biochem Biophys Res Commun. 2018;495(1):755-60. doi: 10.1016/j.bbrc.2017.11.047	China

406	2018	Xu DD, Li XF, Li YH, Liu YH, Huang C, Meng XM, Li J. TIPE2 attenuates liver fibrosis by reversing the activated hepatic stellate cells. Biochem Biophys Res Commun. 2018498(1):199-206. doi: 10.1016/j.bbrc.2017.11.178	China
407	2018	Que R, Shen Y, Ren J, Tao Z, Zhu X, Li Y. Estrogen receptor-β-dependent effects of saikosaponin-d on the suppression of oxidative stress-induced rat hepatic stellate cell activation. Int J Mol Med. 2018;41(3):1357-64. doi: 10.3892/ijmm.2017.3349	China
408	2018	Zhang F, Lu S, He J, Jin H, Wang F, Wu L, Shao J, Chen A, Zheng S. Ligand activation of PPARγ by ligustrazine suppresses pericyte functions of hepatic stellate cells via SMRT-mediated transrepression of HIF-1α. Theranostics 2018;8(3):610-26. doi: 10.7150/thno.22237	China / USA
409	2018	Cao H, Li S, Xie R, Xu N, Qian Y, Chen H, Hu Q, Quan Y, Yu Z, Liu J, Xiang M. Exploring the mechanism of dangguiliuhuang decoction against hepatic fibrosis by network pharmacology and experimental validation. Front Pharmacol. 2018;9:187. doi: 10.3389/fphar.2018.00187	China
410	2018	Pan XY, Yang Y, Meng HW, Li HD, Chen X, Huang HM, Bu FT, Yu HX, Wang Q, Huang C, Meng XM, Li J. DNA methylation of PTGIS enhances hepatic stellate cells activation and liver fibrogenesis. Front Pharmacol. 2018;9:553. doi: 10.3389/fphar.2018.00553	China
411	2018	Jiang ZJ, Shen QH, Chen HY, Yang Z, Shuai MQ, Zheng S. Galectin-1 restores immune tolerance to liver transplantation through activation of hepatic stellate cells. Cell Physiol Biochem. 2018;48(3):863-79. doi: 10.1159/000491955	China
412	2018	Zhang XL, Chen ZN, Huang QF, Bai FC, Nie JL, Lu SJ, Wei JB, Lin X. Methyl helicterate inhibits hepatic stellate cell activation through modulation of apoptosis and autophagy. Cell Physiol Biochem. 2018;51(2):897-908. doi: 10.1159/000495390	China
413	2018	Hu YB, Ye XT, Zhou QQ, Fu RQ. Sestrin 2 attenuates rat hepatic stellate cell (HSC) activation and liver fibrosis via an mTOR/AMPK-dependent mechanism. Cell Physiol Biochem. 2018;51(5):2111-22. doi: 10.1159/000495829	China
414	2018	Xie ZY, Xiao ZH, Wang FF. Inhibition of autophagy reverses alcohol-induced hepatic stellate cells activation through activation of Nrf2-Keap1-ARE signaling pathway. Biochimie 2018;147:55-62. doi: 10.1016/j.biochi.2017.12.013	China
415	2018	Kuo LM, Chen PJ, Sung PJ, Chang YC, Ho CT, Wu YH, Hwang TL. The bioactive extract of Pinnigorgia sp. induces apoptosis of hepatic stellate cells via ROS-ERK/JNK-caspase-3 signaling. Mar Drugs 2018;16(1):19. doi: 10.3390/md16010019	Taiwan
416	2018	Dong Z, Gao Q, Guo H. Glaucocalyxin A attenuates the activation of hepatic stellate cells through the TGF-β1/Smad signaling pathway. DNA Cell Biol. 2018;37(3):227-32. doi: 10.1089/dna.2017.3992	China
417	2018	Song J, Han X, Yao YL, Li YM, Zhang J, Shao DY, Hou LS, Fan Y, Song SZ, Lian LH, Nan JX, Wu YL. Acanthoic acid suppresses lipin1/2 via TLR4 and IRAK4 signalling pathways in EtOH- and lipopolysaccharide-induced hepatic lipogenesis. J Pharm	China

		Pharmacol. 2018;70(3):393-403. doi: 10.1111/jphp.12877	
418	2018	Wu X, Zhi F, Lun W, Deng Q, Zhang W. Baicalin inhibits PDGF-BB-induced hepatic stellate cell proliferation, apoptosis, invasion, migration and activation via the miR-3595/ACSL4 axis. Int J Mol Med. 2018;41(4):1992-2002. doi: 10.3892/ijmm.2018.3427	China
419	2018	Chen E, Cen Y, Lu D, Luo W, Jiang H. IL-22 inactivates hepatic stellate cells via downregulation of the TGF-β1/Notch signaling pathway. Mol Med Rep. 2018;17(4):5449-53. doi: 10.3892/mmr.2018.8516	China
420	2018	Zhou F, Wang A, Li D, Wang Y, Lin L. Pinocembrin from Penthorum chinense Pursh suppresses hepatic stellate cells activation through a unified SIRT3-TGF-β-Smad signaling pathway. Toxicol Appl Pharmacol. 2018;341:38-50. doi: 10.1016/j.taap.2018.01.009	China
421	2018	Sun H, Chen G, Wen B, Sun J, An H, Pang J, Xu W, Yang X, He S. Oligo- peptide I-C-F-6 inhibits hepatic stellate cell activation and ameliorates CCl4-induced liver fibrosis by suppressing NF-κB signaling and Wnt/β-catenin signaling. J Pharmacol Sci. 2018;136(3):133-41. doi: 10.1016/j.jphs.2018.01.003	China
422	2018	Luo H, Zhao F, Zhang F, Liu N. Influence of amygdalin on PDG, IGF and PDGFR expression in HSC-T6 cells. Exp Ther Med. 2018;15(4):3693-8. doi: 10.3892/etm.2018.5886	China
423	2018	Wang XY, Gao H, Xie XJ, Jurhiin J, Zhang MZ, Zhou YP, Liu R, Ning M, Han J, Tang HF. Triterpenoid Saponins from Anemone rivularis var. Flore-Minore and Their Anti-Proliferative Activity on HSC-T6 Cells. Molecules. 2018;23(2):491. doi: 10.3390/molecules23020491	China
424	2018	Bu FT, Chen Y, Yu HX, Chen X, Yang Y, Pan XY, Wang Q, Wu YT, Huang C, Meng XM, Li J. SENP2 alleviates CCl4-induced liver fibrosis by promoting activated hepatic stellate cell apoptosis and reversion. Toxicol Lett. 2018;289:86-98. doi: 10.1016/j.toxlet.2018.03.010	China
425	2018	Qin L, Qin J, Zhen X, Yang Q, Huang L. Curcumin protects against hepatic stellate cells activation and migration by inhibiting the CXCL12/CXCR4 biological axis in liver fibrosis：A study in vitro and in vivo. Biomed Pharmacother. 2018;101:599-607. doi: 10.1016/j.biopha.2018.02.091	China
426	2018	El-Lakkany NM, El-Maadawy WH, Seif El-Din SH, Saleh S, Safar MM, Ezzat SM, Mohamed SH, Botros SS, Demerdash Z, Hammam OA. Antifibrotic effects of gallic acid on hepatic stellate cells: In vitro and in vivo mechanistic study. J Tradit Complement Med. 2018;9(1):45-53. doi: 10.1016/j.jtcme.2018.01.010	Egypt
427	2018	Zhang Y, Yu X, Wang Z, Yin M, Zhao Z, Li Y, Li W. Pokeweed antiviral protein attenuates liver fibrosis in mice through regulating Wnt/Jnk mediated glucose metabolism. Saudi J Gastroenterol. 2018;24(3):157-64. doi: 10.4103/sjg.SJG_470_17	China

428	2018	Gong Z, Tang J, Xiang T, Lin J, Deng C, Peng Y, Zheng J, Hu G. Genome-wide identification of long noncoding RNAs in CCl4-induced liver fibrosis via RNA sequencing. Mol Med Rep. 2018;18(1):299-307. doi: 10.3892/mmr.2018.8986	China
429	2018	Wei X, Chen Y, Huang W. Ginsenoside Rg1 ameliorates liver fibrosis via suppressing epithelial to mesenchymal transition and reactive oxygen species production in vitro and in vivo. Biofactors 2018;44(4):327-35. doi: 10.1002/biof.1432	China
430	2018	Chung YH, Huang YH, Chu TH, Chen CL, Lin PR, Huang SC, Wu DC, Huang CC, Hu TH, Kao YH, Tai MH. BMP-2 restoration aids in recovery from liver fibrosis by attenuating TGF-β1 signaling. Lab Invest. 2018;98(8):999-1013. doi: 10.1038/s41374-018-0069-9	Taiwan
431	2018	Cummins CB, Wang X, Nunez Lopez O, Graham G, Tie HY, Zhou J, Radhakrishnan RS. Luteolin-mediated inhibition of hepatic stellate cell activation via suppression of the STAT3 pathway. Int J Mol Sci. 2018;19(6):1567. doi: 10.3390/ijms19061567	China / USA
432	2018	Ma X, Luo Q, Zhu H, Liu X, Dong Z, Zhang K, Zou Y, Wu J, Ge J, Sun A. Aldehyde dehydrogenase 2 activation ameliorates CCl4-induced chronic liver fibrosis in mice by up-regulating Nrf2/HO-1 antioxidant pathway. J Cell Mol Med. 2018;22(8):3965-78. doi: 10.1111/jcmm.13677	China
433	2018	Cummins CB, Wang X, Xu J, Hughes BD, Ding Y, Chen H, Zhou J, Radhakrishnan RS. Antifibrosis effect of novel oridonin analog CYD0618 via suppression of the NF-κB pathway. J Surg Res. 2018;232:283-92. doi: 10.1016/j.jss.2018.06.040	USA
434	2018	Qin GJ, Zhao YZ, Liu YX, Li C, Cao J, Cheng QC, Xiao XH, Lu Q. [Study on the effects of total flavonoids from litchi nucleus on nuclear translocation of nuclear factor-kappa B and related protein expression in rat hepatic stellate cell]. Zhonghua Gan Zang Bing Za Zhi. 201820;26(7):535-9. Chinese. doi: 10.3760/cma.j.issn.1007-3418.2018.07.011	China
435	2018	Cai X, Li Z, Zhang Q, Qu Y, Xu M, Wan X, Lu L. CXCL6-EGFR-induced Kupffer cells secrete TGF-β1 promoting hepatic stellate cell activation via the SMAD2/BRD4/C-MYC/EZH2 pathway in liver fibrosis. J Cell Mol Med. 2018;22(10):5050-61. doi: 10.1111/jcmm.13787	China
436	2018	Wang X, Niu C, Zhang X, Dong M. Emodin suppresses activation of hepatic stellate cells through p38 mitogen-activated protein kinase and Smad signaling pathways in vitro. Phytother Res. 2018;32(12):2436-6. doi: 10.1002/ptr.6182	China
437	2018	Hu Y, Hu D, Yu H, Xu W, Fu R. Hypoxia-inducible factor 1α and ROCK1 regulate proliferation and collagen synthesis in hepatic stellate cells under hypoxia. Mol Med Rep. 2018;18(4):3997-4003. doi: 10.3892/mmr.2018.9397	China
438	2018	Li Y, Zhu M, Huo Y, Zhang X, Liao M. Anti-fibrosis activity of combination therapy with epigallocatechin gallate, taurine and genistein by regulating glycolysis, gluconeogenesis, and ribosomal and lysosomal signaling pathways in HSC-T6 cells. Exp Ther Med. 2018;16(6):4329-38. doi: 10.3892/etm.2018.6743	China

439	2018	Shi Y, Wu YF, Long CZ, He P, Gu JY, Yang L, Wang YP. [Study of antioxidant effect of cannabinoid receptor type 2 agonist on rat hepatic stellate cell line]. Zhonghua Gan Zang Bing Za Zhi 2018;26(9):660-5. Chinese. doi: 10.3760/cma.j.issn.1007-3418.2018.09.005	China
440	2018	Chen X, Li WX, Chen Y, Li XF, Li HD, Huang HM, Bu FT, Pan XY, Yang Y, Huang C, Meng XM, Li J. Suppression of SUN2 by DNA methylation is associated with HSCs activation and hepatic fibrosis. Cell Death Dis. 2018;9(10):1021. doi: 10.1038/s41419-018-1032-9	China
441	2018	Yu Q, Xiong XQ, Zhao L, Xu TT, Bi H, Fu R, Wang QH. Biodistribution and toxicity assessment of superparamagnetic iron oxide nanoparticles in vitro and in vivo. Curr Med Sci. 2018;38(6):1096-102. doi: 10.1007/s11596-018-1989-8	China
442	2018	Shin GM, Koppula S, Chae YJ, Kim HS, Lee JD, Kim MK, Song M. Anti- hepatofibrosis effect of Allium senescens in activated hepatic stellate cells and thioacetamide-induced fibrosis rat model. Pharm Biol. 2018;56(1):632-42. doi: 10.1080/13880209.2018.1529801	Korea
443	2019	Zhang M, Zhao X, Geng J, Liu H, Zeng F, Qin Y, Li J, Liu C, Wang H. Efficient penetration of Scp01-b and its DNA transfer abilities into cells. J Cell Physiol. 2019;234(5):6539-47. doi: 10.1002/jcp.27392	China / USA
444	2019	Wang Y, Sun Y, Zuo L, Wang Y, Huang Y. ASIC1a promotes high glucose and PDGF-induced hepatic stellate cell activation by inducing autophagy through CaMKKβ/ERK signaling pathway. Toxicol Lett. 2019;300:1-9. doi: 10.1016/j.toxlet.2018.10.003	China
445	2019	Fan Y, Du Z, Steib CJ, Ding Q, Lu P, Tian D, Liu M. Effect of SEPT6 on the biological behavior of hepatic stellate cells and liver fibrosis in rats and its mechanism. Lab Invest. 2019;99(1):17-36. doi: 10.1038/s41374-018-0133-5	China / Germany
446	2019	Lin IY, Chiou YS, Wu LC, Tsai CY, Chen CT, Chuang WC, Lee MC, Lin CC, Lin TT, Chen SC, Pan MH, Ma N. CCM111 prevents hepatic fibrosis via cooperative inhibition of TGF-β, Wnt and STAT3 signaling pathways. J Food Drug Anal. 2019;27(1):184-94. doi: 10.1016/j.jfda.2018.09.008	Taiwan
447	2019	Huang Q, Zhang X, Bai F, Nie J, Wen S, Wei Y, Wei J, Huang R, He M, Lu Z, Lin X. Methyl helicterte ameliorates liver fibrosis by regulating miR-21-mediated ERK and TGF-β1/Smads pathways. Int Immunopharmacol. 2019;66:41-51. doi: 10.1016/j.intimp.2018.11.006. Erratum in: Int Immunopharmacol. 2020;79:106117. PMID: 30419452	China / USA
448	2019	Ullah A, Wang K, Wu P, Oupicky D, Sun M. CXCR4-targeted liposomal mediated co-delivery of pirfenidone and AMD3100 for the treatment of TGFβ-induced HSC-T6 cells activation. Int J Nanomedicine 2019;14:2927-44. doi: 10.2147/IJN.S171280	China / USA
449	2019	Huang C, Gan D, Luo F, Wan S, Chen J, Wang A, Li B, Zhu X. Interaction mechanisms between the NOX4/ROS and RhoA/ROCK1 signaling pathways as new anti-fibrosis targets of ursolic acid in hepatic stellate cells. Front Pharmacol. 2019;10:431. doi: 10.3389/fphar.2019.00431	China

450	2019	Chen Y, Zhou G, Ma B, Tong J, Wang Y. Active constituent in the ethyl acetate extract fraction of Terminalia bellirica fruit exhibits antioxidation, antifibrosis, and proapoptosis capabilities in vitro. Oxid Med Cell Longev. 2019;2019:5176090. doi: 10.1155/2019/5176090	China
451	2019	Li Y, Zhang T, Liu Q, Zhang J, Li R, Pu S, Wu T, Ma L, He J. Mixed micelles loaded with the 5-benzylidenethiazolidine-2,4-dione derivative SKLB023 for efficient treatment of non-alcoholic steatohepatitis. Int J Nanomedicine 2019;14:3943-53. doi: 10.2147/IJN.S202821	China
452	2019	Pan XY, You HM, Wang L, Bi YH, Yang Y, Meng HW, Meng XM, Ma TT, Huang C, Li J. Methylation of RCAN1.4 mediated by DNMT1 and DNMT3b enhances hepatic stellate cell activation and liver fibrogenesis through Calcineurin/NFAT3 signaling. Theranostics 2019;9(15):4308-23. doi: 10.7150/thno.32710	China
453	2019	Yin L, Qi Y, Xu Y, Xu L, Han X, Tao X, Song S, Peng J. Corrigendum: Dioscin inhibits HSC-T6 cell migration via adjusting SDC-4 expression: Insights from iTRAQ-based quantitative proteomics. Front Pharmacol. 2019;10:1036. doi: 10.3389/fphar.2019.01036. Erratum for: Front Pharmacol. 2017;8:665. PMID: 31579122	China
454	2019	Kumar V, Dong Y, Kumar V, Almawash S, Mahato RI. The use of micelles to deliver potential hedgehog pathway inhibitor for the treatment of liver fibrosis. Theranostics 2019;9(25):7537-55. doi: 10.7150/thno.38913	USA
455	2019	Tan YF, Tang L, OuYang WX, Jiang T, Zhang H, Li SJ. β-catenin-coordinated lncRNA MALAT1 up-regulation of ZEB-1 could enhance the telomerase activity in HGF-mediated differentiation of bone marrow mesenchymal stem cells into hepatocytes. Pathol Res Pract. 2019;215(3):546-54. doi: 10.1016/j.prp.2019.01.002	China
456	2019	Yu HX, Yao Y, Bu FT, Chen Y, Wu YT, Yang Y, Chen X, Zhu Y, Wang Q, Pan XY, Meng XM, Huang C, Li J. Blockade of YAP alleviates hepatic fibrosis through accelerating apoptosis and reversion of activated hepatic stellate cells. Mol Immunol. 2019;107:29-40. doi: 10.1016/j.molimm.2019.01.004	China
457	2019	Liu Q, Sun F, Deng Y, Dai R, Lv F. HPLC-ESI-MSn identification and NMR characterization of glucosyloxybenzyl 2R-benzylmalate deriva- Tives from Arundina Graminifolia and their anti-liver fibrotic effects in vitro. Molecules 2019;24(3):525. doi: 10.3390/molecules24030525	China
458	2019	Zuo JJ, Shen CY, Shen BD, Liu Y, Zhong RN, Liu X, Wang XH, Yuan HL. [Preparation and in vitro anti-hepatic fibrosis evaluation of herpetone nanosuspensions]. Zhongguo Zhong Yao Za Zhi 2019;44(6):1164-9. Chinese. doi: 10.19540/j.cnki.cjcmm.20181224.001	China
459	2019	Zhang X, Zhou J, Zhu Y, He L, Pang Z, Wang Z, Xu C, Zhang C, Hao Q, Li W, Zhang W, Zhang Y, Li M. d-amino acid modification protects N-acetyl-seryl- aspartyl-lysyl-proline from physiological hydroxylation and increases its antifibrotic	China

		effects on hepatic fibrosis. IUBMB Life 2019;71(9):1302-12. doi: 10.1002/iub.2037	
460	2019	Wang YZ, Zhang W, Wang YH, Fu XL, Xue CQ. Repression of liver cirrhosis achieved by inhibitory effect of miR-454 on hepatic stellate cells activation and proliferation via Wnt10a. J Biochem. 2019;165(4):361-7. doi: 10.1093/jb/mvy111	China
461	2019	Lin X, Li J, Xing YQ. Geniposide, a sonic hedgehog signaling inhibitor, inhibits the activation of hepatic stellate cell. Int Immunopharmacol. 2019;72:330-8. doi: 10.1016/j.intimp.2019.04.016	China
462	2019	Wei Y, Zhang X, Wen S, Huang S, Huang Q, Lu S, Bai F, Nie J, Wei J, Lu Z, Lin X. Methyl helicterate inhibits hepatic stellate cell activation through downregulating the ERK1/2 signaling pathway. J Cell Biochem. 2019;120(9):14936-45. doi: 10.1002/jcb.28756	China / USA
463	2019	Han J, Zhang X, Lau JK, Fu K, Lau HC, Xu W, Chu ES, Lan H, Yu J. Bone marrow-derived macrophage contributes to fibrosing steatohepatitis through activating hepatic stellate cells. J Pathol. 2019;248(4):488-500. doi: 10.1002/path.5275	China
464	2019	Hsu WH, Liao SC, Chyan YJ, Huang KW, Hsu SL, Chen YC, Siu ML, Chang CC, Chung YS, Huang CF. Graptopetalum paraguayense inhibits liver fibrosis by blocking TGF-β signaling in vivo and in vitro. Int J Mol Sci. 2019;20(10):2592. doi: 10.3390/ijms20102592	Taiwan
465	2019	Liu X, Zhang M, Zhang H, Zhao A, Sun J, Tang W. [Role of PPAR-γ-regulated autophagy in genistein-induced inhibition of hepatic stellate cell activation]. Nan Fang Yi Ke Da Xue Xue Bao 2019;39(5):561-5. Chinese. doi: 10.12122/j.issn.1673-4254.2019.05.10	China
466	2019	Moon MY, Kim HJ, Kim MJ, Uhm S, Park JW, Suk KT, Park JB, Kim DJ, Kim SE. Rap1 regulates hepatic stellate cell migration through the modulation of RhoA activity in response to TGF-β1. Int J Mol Med. 2019;44(2):491-502. doi: 10.3892/ijmm.2019.4215	Korea
467	2019	Wu SM, Li TH, Yun H, Ai HW, Zhang KH. miR-140-3p knockdown suppresses cell proliferation and fibrogenesis in hepatic stellate cells via PTEN-mediated AKT/mTOR signaling. Yonsei Med J. 2019;60(6):561-9. doi: 10.3349/ymj.2019.60.6.561	China
468	2019	Song LY, Ma YT, Fang WJ, He Y, Wu JL, Zuo SR, Deng ZZ, Wang SF, Liu SK. Inhibitory effects of oxymatrine on hepatic stellate cells activation through TGF-β/miR-195/Smad signaling pathway. BMC Complement Altern Med. 2019;19(1):138. doi: 10.1186/s12906-019-2560-2	China
469	2019	Cao G, Zhu R, Jiang T, Tang D, Kwan HY, Su T. Danshensu, a novel indoleamine 2,3-dioxygenase1 inhibitor, exerts anti-hepatic fibrosis effects via inhibition of JAK2-STAT3 signaling. Phytomedicine 2019;63:153055. doi: 10.1016/j.phymed.2019.153055	China
470	2019	Du XS, Li HD, Yang XJ, Li JJ, Xu JJ, Chen Y, Xu QQ, Yang L, He CS, Huang C, Meng XM, Li J. Wogonin attenuates liver fibrosis via regulating hepatic stellate cell activation and apoptosis. Int Immunopharmacol. 2019;75:105671. doi:	China

		10.1016/j.intimp.2019.05.056	
471	2019	Wang Y, Yan D. Plantamajoside exerts antifibrosis effects in the liver by inhibiting hepatic stellate cell activation. Exp Ther Med. 2019;18(4):2421-8. doi: 10.3892/etm.2019.7843	China
472	2019	Ganbold M, Shimamoto Y, Ferdousi F, Tominaga K, Isoda H. Antifibrotic effect of methylated quercetin derivatives on TGFβ-induced hepatic stellate cells. Biochem Biophys Rep. 2019;20:100678. doi: 10.1016/j.bbrep.2019.100678	Japan
473	2019	Ji H, Ji H, Wang B. [Aldosterone promotes migration of rat hepatic stellate cells via activation of RhoA/ROCK signaling pathway]. Xi Bao Yu Fen Zi Mian Yi Xue Za Zhi 2019;35(9):812-6. Chinese. PMID: 31750823	China
474	2019	Chen Z, Yao L, Liu Y, Pan Z, Peng S, Wan G, Cheng J, Wang J, Cao W. Astragaloside IV regulates NF-κB-mediated cellular senescence and apoptosis of hepatic stellate cells to suppress PDGF-BB-induced activation. Exp Ther Med. 2019;18(5):3741-50. doi: 10.3892/etm.2019.8047	China
475	2019	He H, Dai J, Feng J, He Q, Chen X, Dai W, Xu A, Huang H. FBXO31 modulates activation of hepatic stellate cells and liver fibrogenesis by promoting ubiquitination of Smad7. J Cell Biochem. 2019;121(8-9):3711-9. doi: 10.1002/jcb.29528	China
476	2019	Choi JH, Kim SM, Lee GH, Jin SW, Lee HS, Chung YC, Jeong HG. Platyconic acid A, Platycodi Radix-derived saponin, suppresses TGF-1-induced activation of hepatic stellate cells via blocking SMAD and activating the PPAR signaling pathway. Cells 2019;8(12):1544. doi: 10.3390/cells8121544	Korea
477	2019	Zhang T, Hao H, Zhou ZQ, Zeng T, Zhang JM, Zhou XY. Lipoxin A4 inhibited the activation of hepatic stellate cells -T6 cells by modulating profibrotic cytokines and NF-κB signaling pathway. Prostaglandins Other Lipid Mediat. 2020;146:106380. doi: 10.1016/j.prostaglandins.2019.106380	China
478	2019	Tao Y, Qiu T, Yao X, Jiang L, Wang N, Jia X, Wei S, Wang Z, Pei P, Zhang J, Zhu Y, Yang G, Liu X, Liu S, Sun X. Autophagic-CTSB-inflammasome axis modulates hepatic stellate cells activation in arsenic-induced liver fibrosis. Chemosphere 2020;242:124959. doi: 10.1016/j.chemosphere.2019.124959	China
479	2019	Huang D, Dong Z, Sun L, Chen W, Sun L. Two neolignans from Penthorum Chinense and their antiproliferative activities. Nat Prod Res. 2020;34(11):1515-20. doi: 10.1080/14786419.2018.1517261	China
480	2019	Hao LS, Zhang PL, Liu B, Zhang GL, Chen J, Song J, Zhang MT, Jin LM. [Effect of downregulation of phosphatase and tensin homolog gene expression on p130crk- related substrate protein and paxillin signal transduction in activated hepatic stellate cells in vitro]. Zhonghua Gan Zang Bing Za Zhi 2019;27(12):989-93. Chinese. doi: 10.3760/cma.j.issn.1007-3418.2019.12.011	China
481	2020	Rao C, Ni YR, Zhao YM, Zhang YQ, Zhou RT, Liu CB, Han L, Wu JF. Class C1 decoy oligodeoxynucleotide inhibits profibrotic	China

		genes expression in rat hepatic stellate cells. Mol Med Rep. 2020;21(2):667-74. doi: 10.3892/mmr.2019.10881	
482	2020	Zhu H, Shan Y, Ge K, Lu J, Kong W, Jia C. Specific overexpression of mitofusin-2 in hepatic stellate cells ameliorates liver fibrosis in mice model. Hum Gene Ther. 2020;31(1-2):103-9. doi: 10.1089/hum.2019.153	China
483	2020	Yu L, Wang L, Yi H, Wu X. LRP6-CRISPR prevents activation of hepatic stellate cells and liver fibrogenesis in rats. Am J Transl Res. 2020;12(2):397-408. PMID: 32194892	China
484	2020	Du J, Ren W, Zhang Q, Fu N, Han F, Cui P, Li W, Kong L, Zhao S, Wang R, Zhang Y, Yang L, Kong L, Nan Y. Heme oxygenase-1 suppresses Wnt signaling pathway in nonalcoholic steatohepatitis-related liver fibrosis. Biomed Res Int. 2020;2020:4910601. doi: 10.1155/2020/4910601	China
485	2020	Wu JS, Chiu V, Lan CC, Wang MC, Tzeng IS, Kuo CY, Hsieh PC. Chrysophanol prevents lipopolysaccharide-induced hepatic stellate cell activation by upregulating apoptosis, oxidative stress, and the unfolded protein response. Evid Based Complement Alternat Med. 2020;2020:8426051. doi: 10.1155/2020/8426051	Taiwan
486	2020	Chen G, Zhu Y, Liang X, Wang X, Yu W, Guo J, Zhu L, Ma R. The effect of lecithins coupled decorin nanoliposomes on treatment of carbon tetrachloride-induced liver fibrosis. Biomed Res Int. 2020;2020:8815904. doi: 10.1155/2020/8815904	China
487	2020	Chang Y, Xia L, Song M, Tang M, Patpur BK, Li J, Yang W, Yang C. The in vitro effects of phospholipase D1-mTOR axis in liver fibrogenesis. Life Sci. 2020;251:117595. doi: 10.1016/j.lfs.2020.117595	China
488	2020	Zhang Q, Mohammed EAH, Wang Y, Bai Z, Zhao Q, He D, Wang Z. Synthesis and anti-hepaticfibrosis of glycyrrhetinic acid derivatives with inhibiting COX-2. Bioorg Chem. 2020;99:103804. doi: 10.1016/j.bioorg.2020.103804	China
489	2020	Yin L, Zhang Y, Shi H, Feng Y, Zhang Z, Zhang L. Proteomic profiling of hepatic stellate cells in alcohol liver fibrosis reveals proteins involved in collagen production. Alcohol 2020;86:81-91. doi: 10.1016/j.alcohol.2020.02.167	China
490	2020	Liang H, Wang X, Si C, Duan Y, Chen B, Liang H, Yang D. Downregulation of miR-141 deactivates hepatic stellate cells by targeting the PTEN/AKT/mTOR pathway. Int J Mol Med. 2020;46(1):406-14. doi: 10.3892/ijmm.2020.4578	China
491	2020	Hu Z, Su H, Zeng Y, Lin C, Guo Z, Zhong F, Jiang K, Yuan G, He S. Tetramethylpyrazine ameliorates hepatic fibrosis through autophagy-mediated inflammation. Biochem Cell Biol. 2020;98(3):327-37. doi: 10.1139/bcb-2019-0059	China
492	2020	Dong Z, Li S, Si L, Ma R, Bao L, Bo A. Identification lncRNA LOC102551149/miR-23a-5p pathway in hepatic fibrosis. Eur J Clin Invest. 2020;50(6):e13243. doi: 10.1111/eci.13243	China
493	2020	Yang H, Zhao LF, Zhang L, Zhang XH, Zhang XQ. [Rosiglitazone inhibits hepatic stellate cell proliferation by regulating peroxisome proliferator- activated receptor gamma/ heme oxygenase-1 expression]. Zhonghua Gan Zang Bing Za Zhi	China

		2020;28(5):410-5. Chinese. doi: 10.3760/cma.j.cn501113-20190430-00150	
494	2020	Asada K, Kaji K, Sato S, Seki K, Shimozato N, Kawaratani H, Takaya H, Sawada Y, Nakanishi K, Furukawa M, Kitade M, Moriya K, Namisaki T, Noguchi R, Akahane T, Yoshiji H. Hydralazine sensitizes to the antifibrotic effect of 5-aza-2'-deoxycytidine in hepatic stellate cells. Biology (Basel) 2020;9(6):117. doi: 10.3390/biology9060117	Japan
495	2020	Du QH, Zhang CJ, Li WH, Mu Y, Xu Y, Lowe S, Han L, Yu X, Wang SY, Li Y, Li J. Gan Shen Fu Fang ameliorates liver fibrosis in vitro and in vivo by inhibiting the inflammatory response and extracellular signal- regulated kinase phosphorylation. World J Gastroenterol. 2020;26(21):2810-20. doi: 10.3748/wjg.v26.i21.2810	China
496	2020	Liu X, Mi X, Wang Z, Zhang M, Hou J, Jiang S, Wang Y, Chen C, Li W. Ginsenoside Rg3 promotes regression from hepatic fibrosis through reducing inflammation-mediated autophagy signaling pathway. Cell Death Dis. 2020;11(6):454. doi: 10.1038/s41419-020-2597-7	China
497	2020	Noguchi R, Kaji K, Namisaki T, Moriya K, Kawaratani H, Kitade M, Takaya H, Aihara Y, Douhara A, Asada K, Nishimura N, Miyata T, Yoshiji H. Novel oral plasminogen activator inhibitor-1 inhibitor TM5275 attenuates hepatic fibrosis under metabolic syndrome via suppression of activated hepatic stellate cells in rats. Mol Med Rep. 2020;22(4):2948-56. doi: 10.3892/mmr.2020.11360	Japan
498	2020	Xiong J, Ni J, Chen C, Wang K. miR-148a-3p regulates alcoholic liver fibrosis through targeting ERBB3. Int J Mol Med. 2020;46(3):1003-12. doi: 10.3892/ijmm.2020.4655	China
499	2020	Xiao L, Zhang H, Yang X, Mahati S, Wu G, Xiaheding Y, Bao YX, Xiao H. Role of phosphatidylinositol 3-kinase signaling pathway in radiation-induced liver injury. Kaohsiung J Med Sci. 2020;36(12):990-7. doi: 10.1002/kjm2.12279	China
500	2020	Yang YR, Bu FT, Yang Y, Li H, Huang C, Meng XM, Zhang L, Lv XW, Li J. LEFTY2 alleviates hepatic stellate cell activation and liver fibrosis by regulating the TGF-β1/Smad3 pathway. Mol Immunol. 2020;126:31-9. doi: 10.1016/j.molimm.2020.07.012	China
501	2020	Kuo CY, Chiu V, Hsieh PC, Huang CY, Huang SJ, Tzeng IS, Tsai FM, Chen ML, Liu CT, Chen YR. Chrysophanol attenuates hepatitis B virus X protein-induced hepatic stellate cell fibrosis by regulating endoplasmic reticulum stress and ferroptosis. J Pharmacol Sci. 2020;144(3):172-82. doi: 10.1016/j.jphs.2020.07.014	Taiwan
502	2020	Ma Y, Li Y, Zhang H, Wang Y, Wu C, Huang W. Malvidin induces hepatic stellate cell apoptosis via the endoplasmic reticulum stress pathway and mitochondrial pathway. Food Sci Nutr. 2020;8(9):5095-106. doi: 10.1002/fsn3.1810	China
503	2020	Kao YH, Lin YC, Lee PH, Lin CW, Chen PH, Tai TS, Chang YC, Chou MH, Chang CY, Sun CK. Infusion of human mesenchymal stem cells improves regenerative niche in thioacetamide-injured mouse liver. Tissue Eng Regen Med. 2020;17(5):671-82. doi: 10.1007/s13770-020-00274-4	Taiwan

504	2020	Zhu H, He C, Zhao H, Jiang W, Xu S, Li J, Ma T, Huang C. Sennoside A prevents liver fibrosis by binding DNMT1 and suppressing DNMT1-mediated PTEN hypermethylation in HSC activation and proliferation. FASEB J. 2020;34(11):14558-71. doi: 10.1096/fj.202000494RR	China
505	2020	Zhu Y, Pan X, Du N, Li K, Hu Y, Wang L, Zhang J, Liu Y, Zuo L, Meng X, Hu C, Wu X, Jin J, Wu W, Chen X, Wu F, Huang Y. ASIC1a regulates miR-350/SPRY2 by N6 -methyladenosine to promote liver fibrosis. FASEB J. 2020;34(11):14371-88. doi: 10.1096/fj.202001337R	China
506	2020	Zhu L, Mou Q, Wang Y, Zhu Z, Cheng M. Resveratrol contributes to the inhibition of liver fibrosis by inducing autophagy via the microRNA-20a-mediated activation of the PTEN/PI3K/AKT signaling pathway. Int J Mol Med. 2020;46(6):2035-46. doi: 10.3892/ijmm.2020.4748	China
507	2020	Cuiqiong W, Chao X, Xinling F, Yinyan J. Schisandrin B suppresses liver fibrosis in rats by targeting miR-101-5p through the TGF-β signaling pathway. Artif Cells Nanomed Biotechnol. 2020;48(1):473-8. doi: 10.1080/21691401.2020.1717507	China
508	2020	Gu YF, Zhang Y, Yue FL, Li ST, Zhang ZQ, Li J, Bai X. Synthesis of novel 2-(pyridin-2-yl) pyrimidine derivatives and study of their anti-fibrosis activity. Molecules 2020;25(22):5226. doi: 10.3390/molecules25225226	China
509	2021	Li J, Dong S, Ye M, Peng G, Luo J, Wang C, Wang J, Zhao Q, Chang Y, Wang H. MicroRNA-489-3p represses hepatic stellate cells activation by negatively regulating the JAG1/Notch3 signaling pathway. Dig Dis Sci. 2021;66(1):143-50. doi: 10.1007/s10620-020-06174-w	China
510	2021	An L, Lin Y, Li L, Kong M, Lou Y, Wu J, Liu Z. Integrating network pharmacology and experimental validation to investigate the effects and mechanism of Astragalus flavonoids against hepatic fibrosis. Front Pharmacol. 2021;11:618262. doi: 10.3389/fphar.2020.618262	China
511	2021	Lin X, Li Y, Zhang X, Wei Y, Wen S, Lu Z, Huang Q, Wei J. Tormentic acid inhibits hepatic stellate cells activation via blocking PI3K/Akt/mTOR and NF-κB signalling pathways. Cell Biochem Funct. 2021;39(1):77-87. doi: 10.1002/cbf.3564. *Retraction in: Cell Biochem Funct. 2021;* PMID: 32564421	China / USA
512	2021	Lee IH, Im E, Lee HJ, Sim DY, Lee JH, Jung JH, Park JE, Shim BS, Kim SH. Apoptotic and antihepatofibrotic effect of honokiol via activation of GSK3β and suppression of Wnt/β-catenin pathway in hepatic stellate cells. Phytother Res. 2021;35(1):452-62. doi: 10.1002/ptr.6824	Korea
513	2021	Jia WQ, Zhou TC, Dai JW, Liu ZN, Zhang YF, Zang DD, Lv XW. CD73 regulates hepatic stellate cells activation and proliferation through Wnt/β-catenin signaling pathway. Eur J Pharmacol. 2021;890:173667. doi: 10.1016/j.ejphar.2020.173667	China
514	2021	Li B, Liu J, Xin X, Zhang L, Zhou J, Xia C, Zhu W, Yu H. MiR-34c promotes hepatic stellate cell activation and liver fibrogenesis	China

		by suppressing ACSL1 expression. Int J Med Sci. 2021;18(3):615-25. doi: 10.7150/ijms.51589	
515	2021	Kong L, Huang H, Luan S, Liu H, Ye M, Wu F. Inhibition of ASIC1a-mediated ERS improves the activation of HSCs and copper transport under copper load. Front Pharmacol. 2021;12:653272. doi: 10.3389/fphar.2021.653272	China
516	2021	Fang B, Wen S, Li Y, Bai F, Wei Y, Xiong Y, Huang Q, Lin X. Prediction and verification of target of helenalin against hepatic stellate cell activation based on miR-200a-mediated PI3K/Akt and NF-κB pathways. Int Immunopharmacol. 2021;92:107208. doi: 10.1016/j.intimp.2020.107208	China
517	2021	Sommerhalder C, Cummins CB, Wang X, Ramdas D, Lopez ON, Gu Y, Zhou J, Radhakrishnan RS. HJC0416 attenuates fibrogenesis in activated hepatic stellate cells via STAT3 and NF-κB pathways. J Surg Res. 2021;261:334-42. doi: 10.1016/j.jss.2020.12.045	USA
518	2021	Zhang SP, Zhang C, Li LH, Chen YY, Zhu L, Liu XP, Li P. [Prediction of anti-liver fibrosis effect of Piperis Longi Fructus based on network pharmacology]. Zhongguo Zhong Yao Za Zhi 2021;46(4):845-54. Chinese. doi: 10.19540/j.cnki.cjcmm.20201118.402	China
519	2021	Gao HJ, Pang HS, Sun XD, Zhang T, Jing T, Wang XL, Mo XJ, Hu W. [Effects of persistent Echinococcus multilocularis infections on hepatic fibrosis in mice]. Zhongguo Xue Xi Chong Bing Fang Zhi Za Zhi 2021;33(1):54-61. Chinese. doi: 10.16250/j.32.1374.2020282	China
520	2021	Huang W, Ji R, Ge S, Zhou D, Liu Z, Sun Y, Huang W, Lu C. MicroRNA-92b-3p promotes the progression of liver fibrosis by targeting CREB3L2 through the JAK/STAT signaling pathway. Pathol Res Pract. 2021;219:153367. doi: 10.1016/j.prp.2021.153367	China
521	2021	Mo C, Xie S, Zeng T, Lai Y, Huang S, Zhou C, Yan W, Huang S, Gao L, Lv Z. Ginsenoside-Rg1 acts as an IDO1 inhibitor, protects against liver fibrosis via alleviating IDO1-mediated the inhibition of DCs maturation. Phytomedicine 2021;84:153524. doi: 10.1016/j.phymed.2021.153524	China
522	2021	Cai S, Wu L, Yuan S, Liu G, Wang Y, Fang L, Xu D. Carvacrol alleviates liver fibrosis by inhibiting TRPM7 and modulating the MAPK signaling pathway. Eur J Pharmacol. 2021;898:173982. doi: 10.1016/j.ejphar.2021.173982	China
523	2021	Li Y, Luo Y, Zhang X, Lin X, He M, Liao M. Correction: Yan, L., et al. Combined taurine, epigallocatechin gallate and genistein therapy reduces HSC-T6 cell proliferation and modulates the expression of fibrogenic factors. Int. J. Mol. Sci. 2013, 14(10), 20543-54. doi: 10.3390/ijms22052343. Erratum: Int J Mol Sci. 2021;22(5):2343. PMID: 33653018	China
524	2021	Zhang YJ, He Q, You SJ, Ma JJ, Wu XM, Liu C. Mechanism of hepatic stellate cell proliferation and apoptosis in rats by promoting circulation, removing stasis, and dredging collaterals based on the theory of liver collateral disease. Ann Palliat Med. 2021;10(4):3648-56. doi: 10.21037/apm-20-1545	China

525	2021	Liang J, Yuan H, Xu L, Wang F, Bao X, Yan Y, Wang H, Zhang C, Jin R, Ma L, Zhang J, Huri L, Su X, Xiao R, Ma Y. Study on the effect of Mongolian medicine Qiwei Qinggan powder on hepatic fibrosis through JAK2/STAT3 pathway. Biosci Biotechnol Biochem. 2021;85(4):775-85. doi: 10.1093/bbb/zbab001	China
526	2021	Park JW, Kim MJ, Kim SE, Kim HJ, Jeon YC, Shin HY, Park SJ, Jang MK, Kim DJ, Park CK, Choi EK. Increased expression of S100B and RAGE in a mouse model of bile duct ligation-induced liver fibrosis. J Korean Med Sci. 2021;36(14):e90. doi: 10.3346/jkms.2021.36.e90	Korea
527	2021	Cui B, Yang Z, Wang S, Guo M, Li Q, Zhang Q, Bi X. The protective role of protocatechuic acid against chemically induced liver fibrosis in vitro and in vivo. Pharmazie 2021;76(5):232-8. doi: 10.1691/ph.2021.0909	China
528	2021	Shuai C, Xia GQ, Yuan F, Wang S, Lv XW. CD39-mediated ATP-adenosine signalling promotes hepatic stellate cell activation and alcoholic liver disease. Eur J Pharmacol. 2021;905:174198. doi: 10.1016/j.ejphar.2021.174198	China
529	2021	Wang H, Che J, Cui K, Zhuang W, Li H, Sun J, Chen J, Wang C. Schisantherin A ameliorates liver fibrosis through TGF-β1mediated activation of TAK1/MAPK and NF-κB pathways in vitro and in vivo. Phytomedicine 2021;88:153609. doi: 10.1016/j.phymed.2021.153609	China
530	2021	Zhang C, Li L, Hou S, Shi Z, Xu W, Wang Q, He Y, Gong Y, Fang Z, Yang Y. Astragaloside IV inhibits hepatocellular carcinoma by continually suppressing the development of fibrosis and regulating pSmad3C/3L and Nrf2/HO-1 pathways. J Ethnopharmacol. 2021;279:114350. doi: 10.1016/j.jep.2021.114350	China
531	2021	Fan X, Deng J, Shi T, Wen H, Li J, Liang Z, Lei F, Liu D, Zhang H, Liang Y, Hao X, Wang Z. Design, synthesis and bioactivity study of evodiamine derivatives as multifunctional agents for the treatment of hepatocellular carcinoma. Bioorg Chem. 2021;114:105154. doi: 10.1016/j.bioorg.2021.105154	China
532	2021	Huang Z, Ding M, Dong Y, Ma M, Song X, Liu Y, Gao Z, Guan H, Chu Y, Feng H, Wang X, Liu H. Targeted truncated TGF-β receptor type II delivery to fibrotic liver by PDGFβ receptor-binding peptide modification for improving the anti- fibrotic activity against hepatic fibrosis in vitro and in vivo. Int J Biol Macromol. 2021;188:941-9. doi: 10.1016/j.ijbiomac.2021.08.055	China
533	2021	Jiang H, Liu J, Zhang K, Zeng Q. Saikosaponin D inhibits the proliferation and promotes the apoptosis of rat hepatic stellate cells by inducing autophagosome formation. Evid Based Complement Alternat Med. 2021;2021:5451758. doi: 10.1155/2021/5451758	China
534	2021	Zhang J, Yang L, Han X, Li C, Liu R, Ma Z, Han B, Xie R, Yang Q. [Endoplasmic reticulum stress in hepatic stellate cells induced by tunicamycin promotes apoptosis and cell cycle arrest]. Xi Bao Yu Fen Zi Mian Yi Xue Za Zhi. 2021;37(9):794-800. PMID: 34533126	China

535	2021	Zhang C, Yan Y, Gao X, Ma Y. [Therapeutic mechanism of the Mongolian medicine Qiwei Qinggan Powder against liver fibrosis based on UHPLC-TOF-MS combined with network pharmacological methods]. Nan Fang Yi Ke Da Xue Xue Bao. 2021;41(8):1131-41. doi: 10.12122/j.issn.1673-4254.2021.08.02	China
536	2021	Gu Z, Fang L, Ma P. The angiotensin-converting enzyme inhibitor, captopril, suppressed hepatic stellate cell activation via NF-kappaB or wnt3α/β-catenin pathway. Bioengineered 2021;12(1):8370-7. doi: 10.1080/21655979.2021.1987091	China
537	2021	Wu TH, Wang PW, Lin TY, Yang PM, Li WT, Yeh CT, Pan TL. Antioxidant properties of red raspberry extract alleviate hepatic fibrosis via inducing apoptosis and transdifferentiation of activated hepatic stellate cells. Biomed Pharmacother. 2021;144:112284. doi: 10.1016/j.biopha.2021.112284	Taiwan
538	2021	Zhang L, Gao J, Zhou D, Wang X, Li J, Wang J, Chen H, Xie X, Chen T. Profiles of messenger RNAs and MicroRNAs in hypoxia-induced hepatic stellate cells. Ann Transl Med. 2021;9(18):1451. doi: 10.21037/atm-21-4215	China
539	2021	Li Z, Zhao L, Xia Y, Chen J, Hua M, Sun Y. Schisandrin B attenuates hepatic stellate cell activation and promotes apoptosis to protect against liver fibrosis. Molecules 2021;26(22):6882. doi: 10.3390/molecules26226882	China
540	2021	Lee SW, Kim SM, Hur W, Kang BY, Lee HL, Nam H, Yoo SH, Sung PS, Kwon JH, Jang JW, Kim SJ, Yoon SK. Tenofovir disoproxil fumarate directly ameliorates liver fibrosis by inducing hepatic stellate cell apoptosis via downregulation of PI3K/Akt/mTOR signaling pathway. PLoS One 2021;16(12):e0261067. doi: 10.1371/journal.pone.0261067	China
541	2022	Krishnan V, Subramaniam S, Chia-Chuan C, Venkatachalam B, Thomas Cheeran A, Chi-Ying F H. Anticancer activity of Leonurus sibiricus L.: Possible involvement of intrinsic apoptotic pathway. Nutr Cancer 2022:74(1):225-36. doi: 10.1080/01635581.2020.1870702	India / Taiwan
542	2022	Yuan S, Wei C, Liu G, Zhang L, Li J, Li L, Cai S, Fang L. Sorafenib attenuates liver fibrosis by triggering hepatic stellate cell ferroptosis via HIF-1α/SLC7A11 pathway. Cell Prolif. 2022;55(1):e13158. doi: 10.1111/cpr.13158	China
543	2022	Fu W, Zhao Y, Xie J, Yang Y, Xiao P. Identification of anti-hepatic fibrosis components in Periplaneta americana based on spectrum-effect relationship and chemical component separation. Biomed Chromatogr. 2022;36(3):e5286. doi: 10.1002/bmc.5286	Korea
544	2022	Lin L, Li J, Yang S, Zhang R, Hu C, Chen Y, Tian Z, Ma W, Feng Y, Liu N, Liu J, Yang Y, Chen T, Zhao Y, He Y, Yan T. MAPK p38/Ulk1 pathway inhibits autophagy and induces IL-1β expression in hepatic stellate cells. Am J Physiol Gastrointest Liver Physiol. 2022;322(3):G360-G367. doi: 10.1152/ajpgi.00230.2021	China
545	2022	Yang H, Zhang L, Chen J, Zhang X, Zhao Z, Zhao L. Heme Oxygenase-1 inhibits the proliferation of hepatic stellate cells by activating PPARγ and suppressing NF-κB. Comput Math Methods Med. 2022;2022:8920861. doi: 10.1155/2022/8920861	China

546	2022	Chen Z, Lin Z, Yu J, Zhong H, Zhuo X, Jia C, Wan Y. Mitofusin-2 restrains hepatic stellate cells' proliferation via PI3K/Akt signaling pathway and inhibits liver fibrosis in rats. J Healthc Eng. 2022;2022:6731335. doi: 10.1155/2022/6731335	China
547	2022	Wan LY, Peng H, Ni YR, Jiang XP, Wang JJ, Zhang YQ, Ma L, Li R, Han L, Tan Y, Li JM, Cai WL, Yuan WF, Liang JJ, Huang L, Wu X, Zhou Q, Cheng QN, Yang X, Liu MY, Ai WB, Liu CB, Zhang H, Wu JF. The miR-23b/27b/24-1 cluster inhibits hepatic fibrosis by inactivating hepatic stellate cells. Cell Mol Gastroenterol Hepatol. 2022;13(5):1393-412. doi: 10.1016/j.jcmgh.2022.01.016	China
548	2022	He Z, Chen S, Pan T, Li A, Wang K, Lin Z, Liu W, Wang Y, Wang Y. Ginsenoside Rg2 ameliorating CDAHFD-induced hepatic fibrosis by regulating AKT/mTOR-mediated autophagy. J Agric Food Chem. 2022;70(6):1911-22. doi: 10.1021/acs.jafc.1c07578	China
549	2022	Zhang T, Yang Y, Wang B, Wang L, Wang D, Cao N, Shi J. XSSJS inhibits hepatic fibrosis by promoting the miR-29b-3p/VEGFA axis in vitro and in vivo. Biosci Rep. 2022;42(2):BSR20212241. doi: 10.1042/BSR20212241	China
550	2022	Hao LS, Song J, Zhang MT, Song XJ, Jiang MY, Ji JX, Mo YB, Wang J. [Effects of adenovirus-mediated shRNA down-regulates PTEN expression on fibril-binding proteins vinculin, filamin A and cortactin in activated hepatic stellate cells]. Zhonghua Gan Zang Bing Za Zhi. 2022;30(1):38-44. doi: 10.3760/cma.j.cn501113-20201230-00691	China
551	2022	Liu Z, Xiang H, Xiang D, Xiao S, Xiang H, Xiao J, Ren H, Hu P, Liu H, Peng M. Revealing potential anti-fibrotic mechanism of Ganxianfang formula based on RNA sequence. Chin Med. 2022;17(1):23. doi: 10.1186/s13020-022-00579-7	China
552	2022	Chen D, Chen J, Chen Y, Chen F, Wang X, Huang Y. Interleukin-10 regulates starvation-induced autophagy through the STAT3-mTOR-p70s6k axis in hepatic stellate cells. Exp Biol Med (Maywood) 2022:15353702221080435. doi: 10.1177/15353702221080435	China
553	2022	Gao J, Shi Y, Han Y, Tang X, Bi R, Pan L, Lai X. One-way intestinal perfusion of PVP/VA-poloxamer 188-curcuma longa L. extract solid dispersion in rats in vivo and its effect on HSC-T6 cell proliferation. AAPS PharmSciTech. 2022;23(3):83. doi: 10.1208/s12249-022-02228-6	China
554	2022	Li J, Feng W, Dai R, Li B. Rational design, synthesis and activities of phenanthrene derivatives against hepatic fibrosis. Fitoterapia 2022:105176. doi: 10.1016/j.fitote.2022.105176	China
555	2022	Rauchbach E, Zeigerman H, Abu-Halaka D, Tirosh O. Cholesterol induces oxidative stress, mitochondrial damage and death in hepatic stellate cells to mitigate liver fibrosis in mice model of NASH. Antioxidants (Basel) 2022;11(3):536. doi: 10.3390/antiox11030536	Israel
556	2022	Jiang YC, Han X, Dou JY, Yuan MH, Zhou MJ, Cui ZY, Lian LH, Nan JX, Zhang X, Wu YL. Protective role of Siberian onions against toxin-induced liver dysfunction: an insight into health-promoting effects. Food Funct. 2022;13(8):4678-90. doi:	China

		10.1039/d1fo04404d	
557	2022	Qiu JL, Zhang GF, Chai YN, Han XY, Zheng HT, Li XF, Duan F, Chen LY. Ligustrazine attenuates liver fibrosis by targeting miR-145 mediated TGF-β/Smad signaling in an animal model of biliary atresia. J Pharmacol Exp Ther. 2022:JPET-AR-2021-001020. doi: 10.1124/jpet.121.001020. Epub ahead of print.	China
558	2022	Li BL, Liang HJ, Li QR, Wang Q, Ao ZY, Fan YW, Zhang WJ, Lian X, Chen JY, Yuan J, Wu JW. Euryachincoside, a novel phenolic glycoside with anti-hepatic fibrosis activity from Eurya chinensis. Planta Med. 2022. doi: 10.1055/a-1828-2671. Epub ahead of print.	China
559	2022	Liu Z, Zhou S, Zhang Y, Zhao M. Rat bone marrow mesenchymal stem cells (BMSCs) inhibit liver fibrosis by activating GSK3β and inhibiting the Wnt3a/β-catenin pathway. Infect Agent Cancer. 2022;17(1):17. doi: 10.1186/s13027-022-00432-4	China
560	2022	Huang S, Wang Y, Xie S, Lai Y, Mo C, Zeng T, Kuang S, Zhou C, Zeng Z, Chen Y, Huang S, Gao L, Lv Z. Isoliquiritigenin alleviates liver fibrosis through caveolin-1-mediated hepatic stellate cells ferroptosis in zebrafish and mice. Phytomedicine 2022;101:154117. doi: 10.1016/j.phymed.2022.154117. Epub ahead of print.	China
561	2022	Chen S, He Z, Xie W, Chen X, Lin Z, Ma J, Liu Z, Yang S, Wang Y. Ginsenoside Rh2 attenuates CDAHFD-induced liver fibrosis in mice by improving intestinal microbial composition and regulating LPS-mediated autophagy. Phytomedicine 2022;101:154121. doi: 10.1016/j.phymed.2022.154121. Epub ahead of print.	China
562	2022	Schröder SK, Schüler HM, Petersen KV, Tesauro C, Knudsen BR, Pedersen FS, Krus F, Buhl EM, Roeb E, Roderfeld M, Borojevic R, Almeida JL, Weiskirchen R. Genetic and molecular characterization of the immortalized murine hepatic stellate cell line GRX. Cells 2022;11:1504. doi: 10.3390/cells11091504	Germany / Denmark / USA / Brazil
