# Supplementary material for: Genetic Characterization of Rat Hepatic Stellate Cell Line HSC-T6 for In Vitro Cell Line Authentication
Source: Cells. 2022 May 29;11(11):1783. doi: 10.3390/cells11111783 (PMC9179542; doi:10.3390/cells11111783)
Supplement: Supplementary file 1 [file cells-11-01783-s001.zip › Table S2.pdf]

Table S2. Primers used in this study

Gene	Forward primer (5′->3′)	Reverse primer (5′->3′)
mActa2	TGACAGAGGCACCACTGAACC	TCCAGAGTCCAGCACAATACCAGT
rActa2	GAGGAGCATCCGACCTTGC	ATTTTCTCCCGGTTGGCC
*Fth1**	TGGAGTTGTATGCCTCCTACG	TGGAGAAAGTATTTGGCAAAGTT
*Col1a1**	CATGTTCAGCTTTGTGGACCT	GCAGCTGACTTCAGGGATGT
mCtgf/Ccn2	TGACCTGGAGGAAAACATTAAGA	AGCCCTGTATGTCTTCACACTG
rCtgf/Ccn2	GCTGACCTAGAGGAAAACATTAAGA	CCGGTAGGTCTTCACACTGG
rGfap	TTTCTCCAACCTCCAGATCC	TCTTGAGGTGGCCTTCTGAC
rTgfbr2	GAGGACGGCCTGAAATCC	CTCACACACGATCTGGATGC
rTgfb1	CCTGGAAAGGGCTCAACAC	CAGTTCTTCTCTGTGGAGCTGA
*Gapdh**	AACCTGCCAAGTATGATGACATCA	TGTTGAAGTCACAGGAGACAACCT

* given primer pair matches mouse and rat gene sequences
