# Supplementary figures and images for: Genetic Characterization of Rat Hepatic Stellate Cell Line HSC-T6 for In Vitro Cell Line Authentication
Source: Cells. 2022 May 29;11(11):1783. doi: 10.3390/cells11111783 (PMC9179542; doi:10.3390/cells11111783)

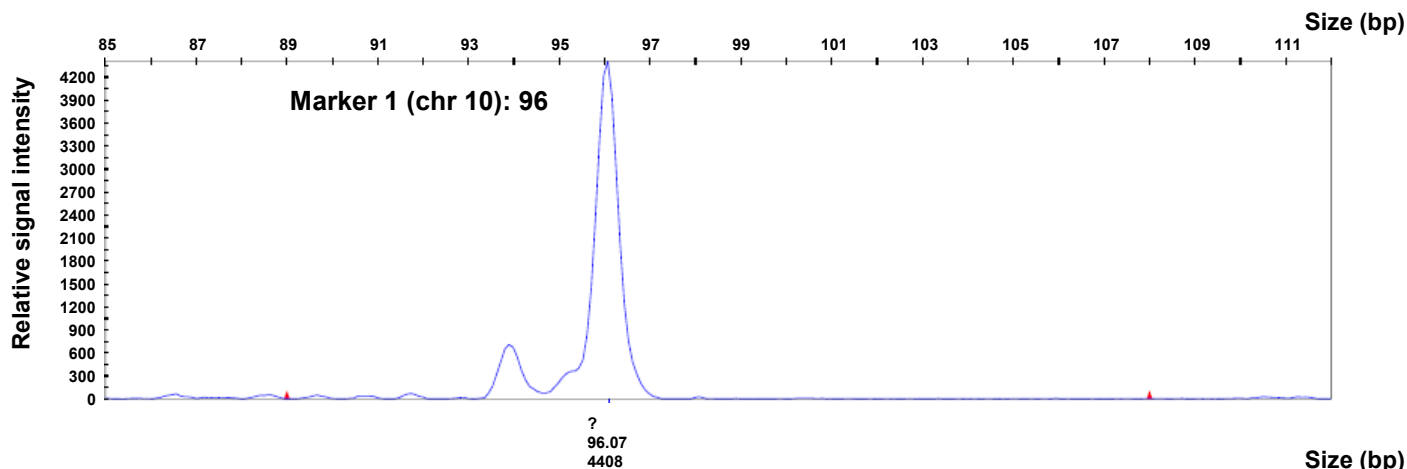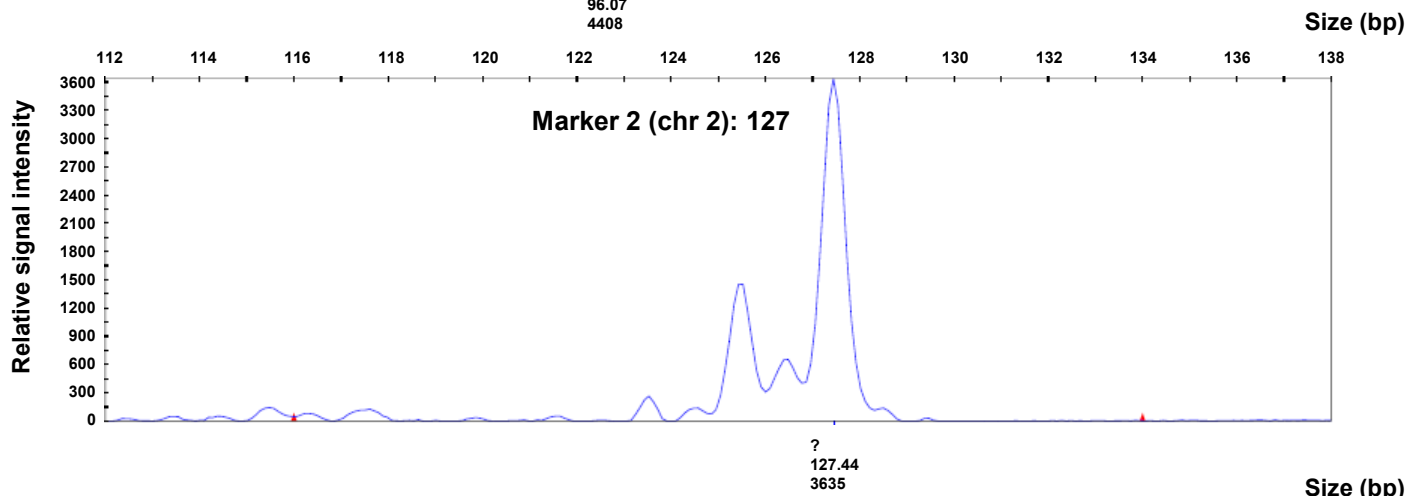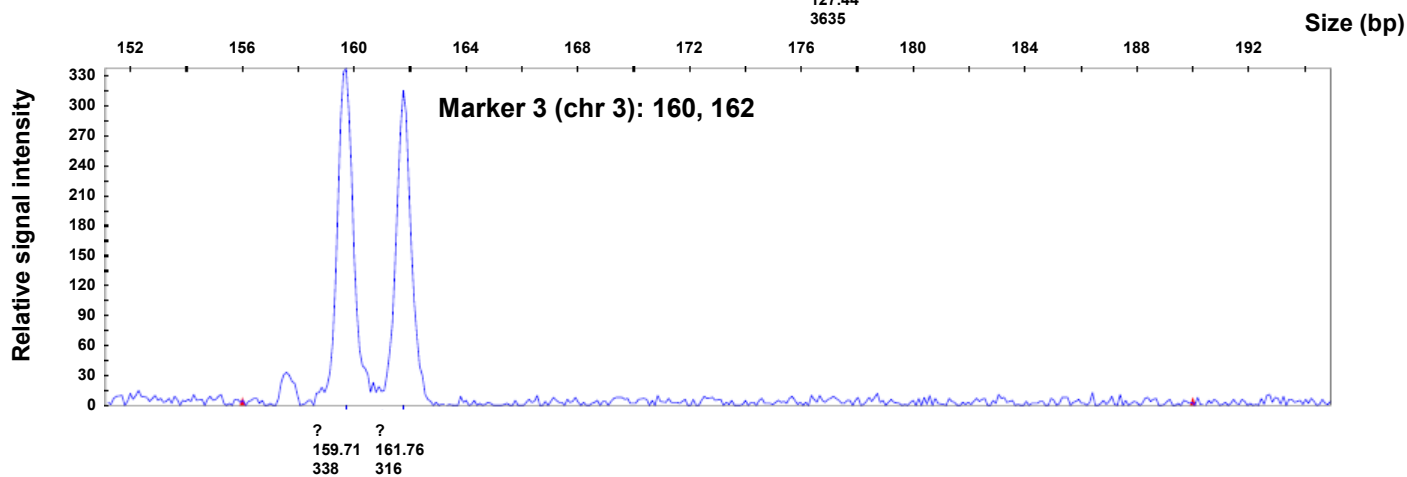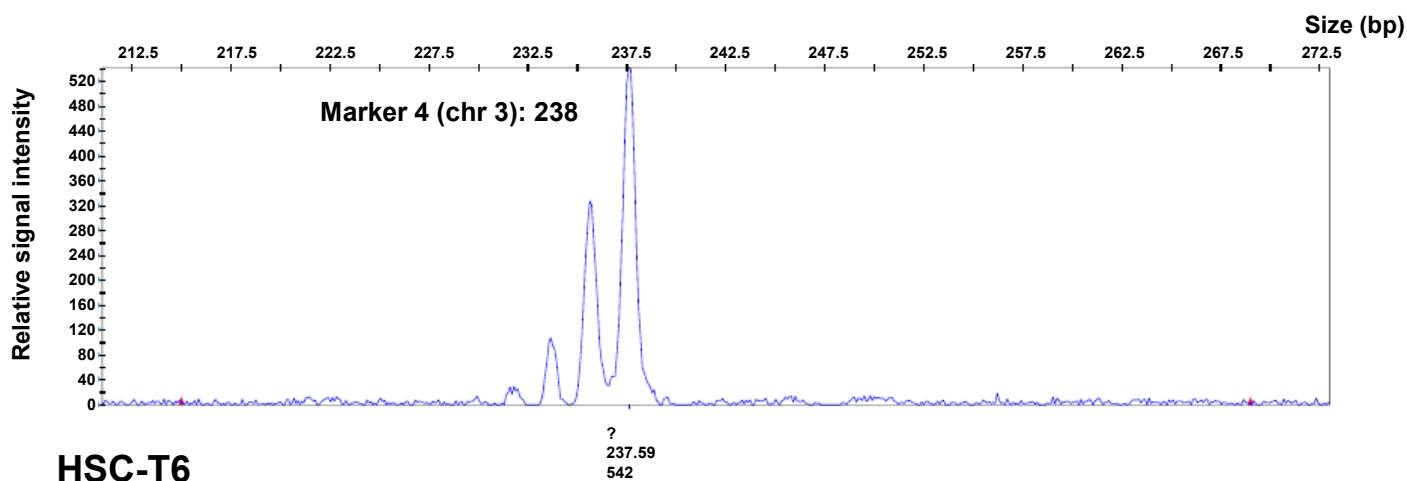

HSC-T6

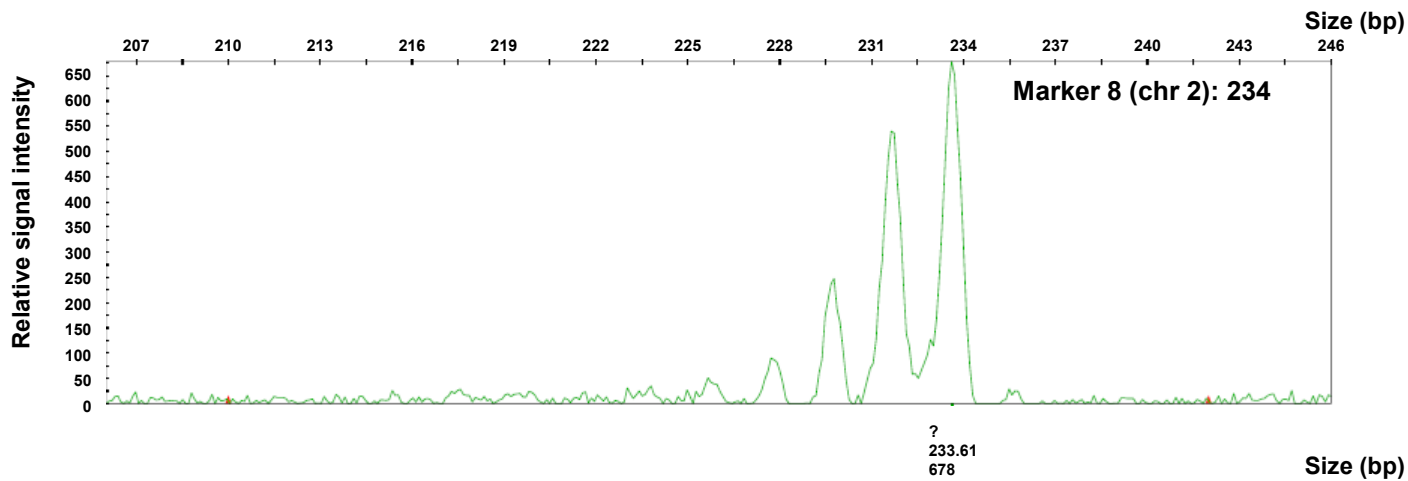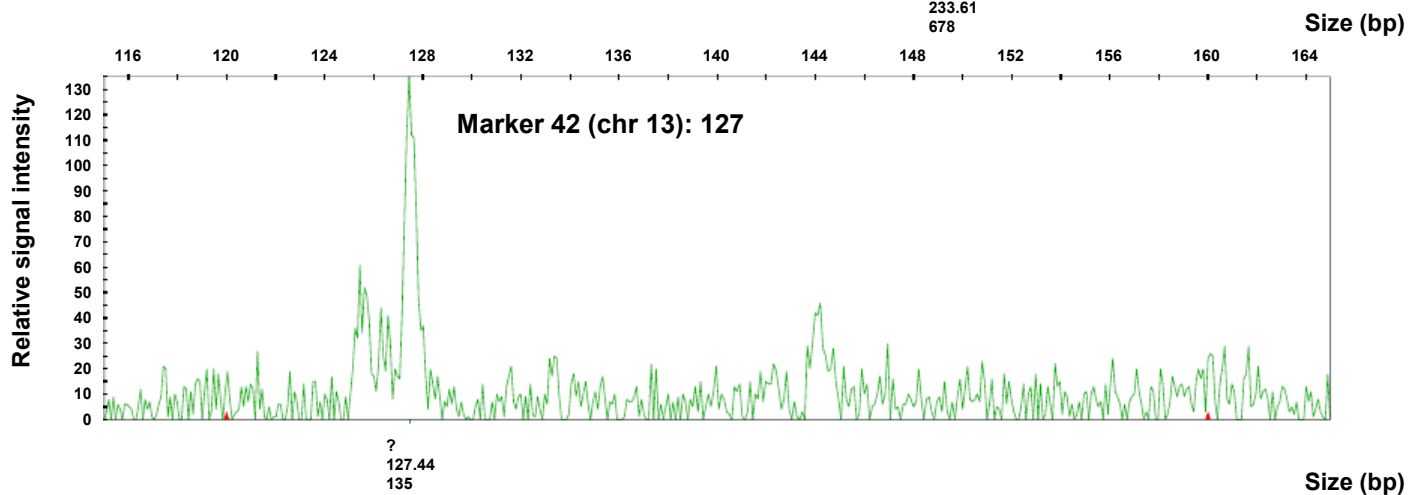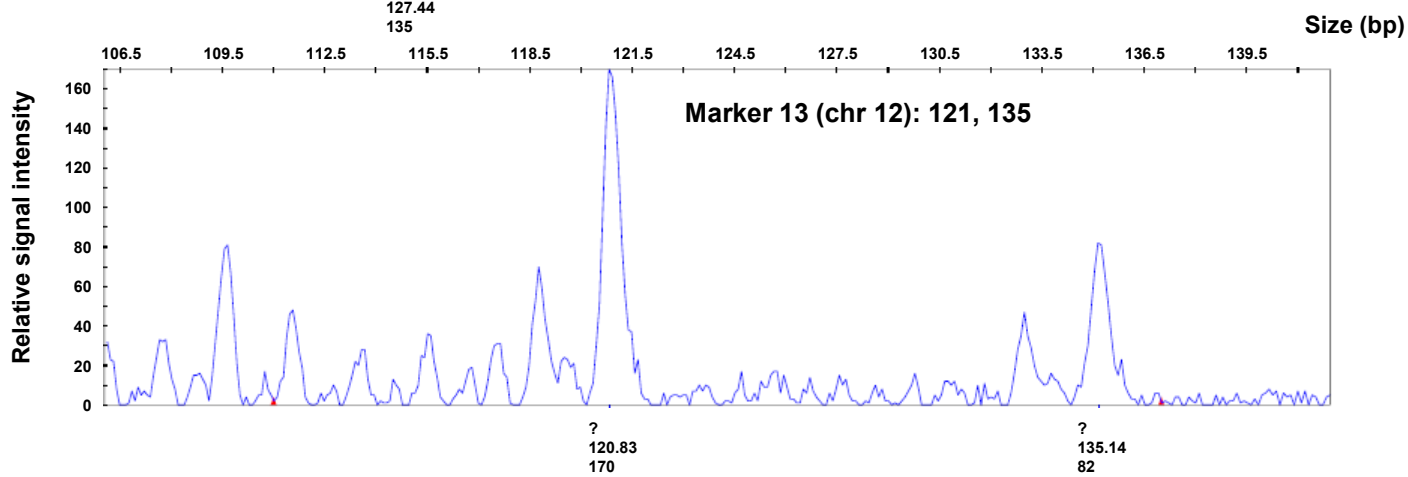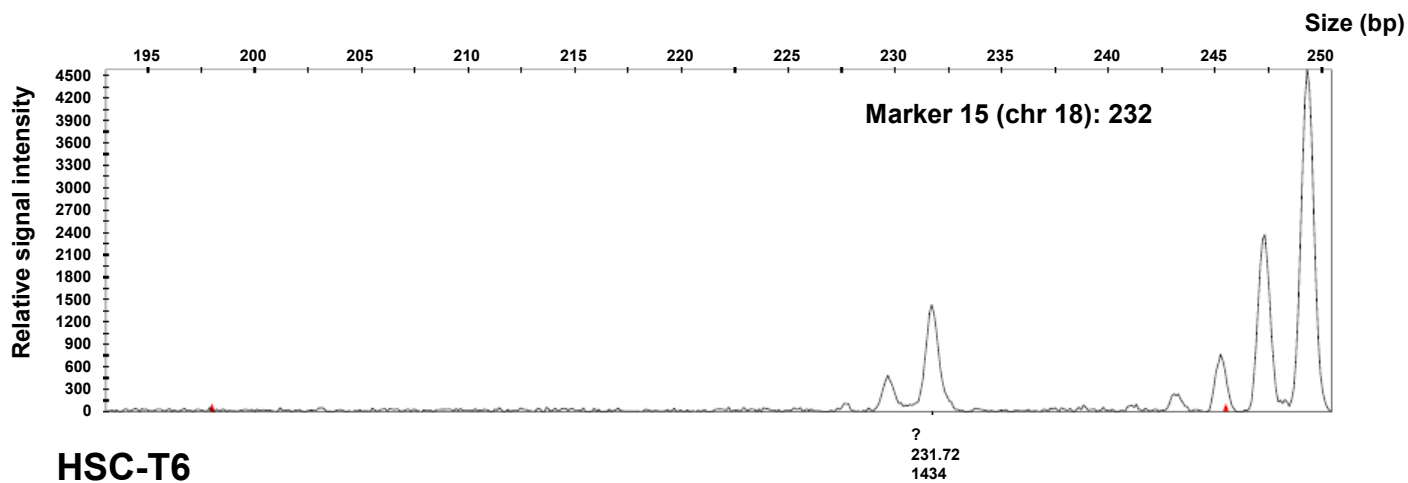

HSC-T6

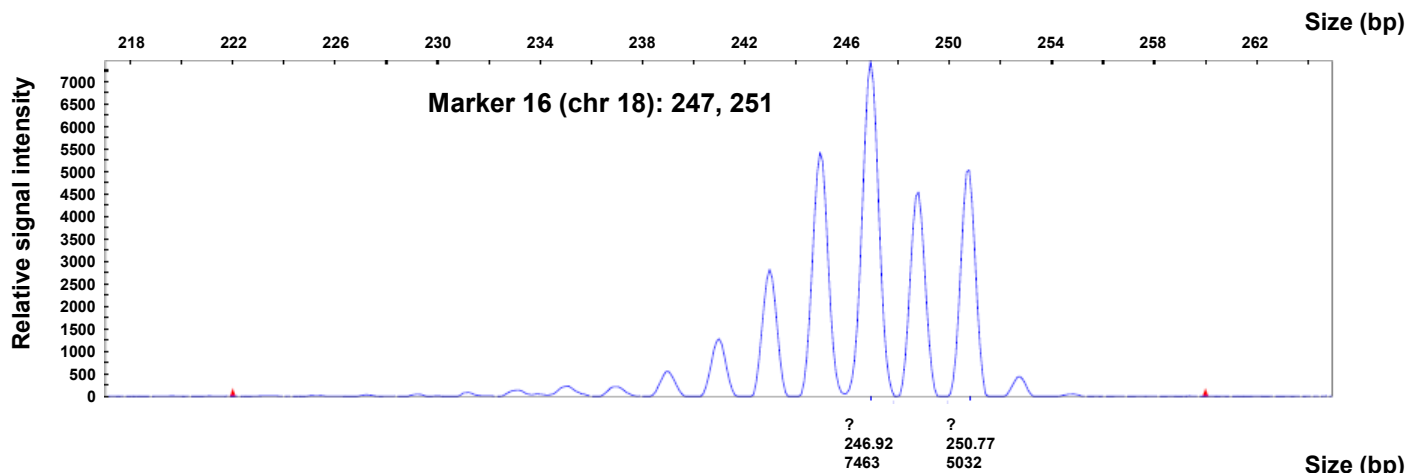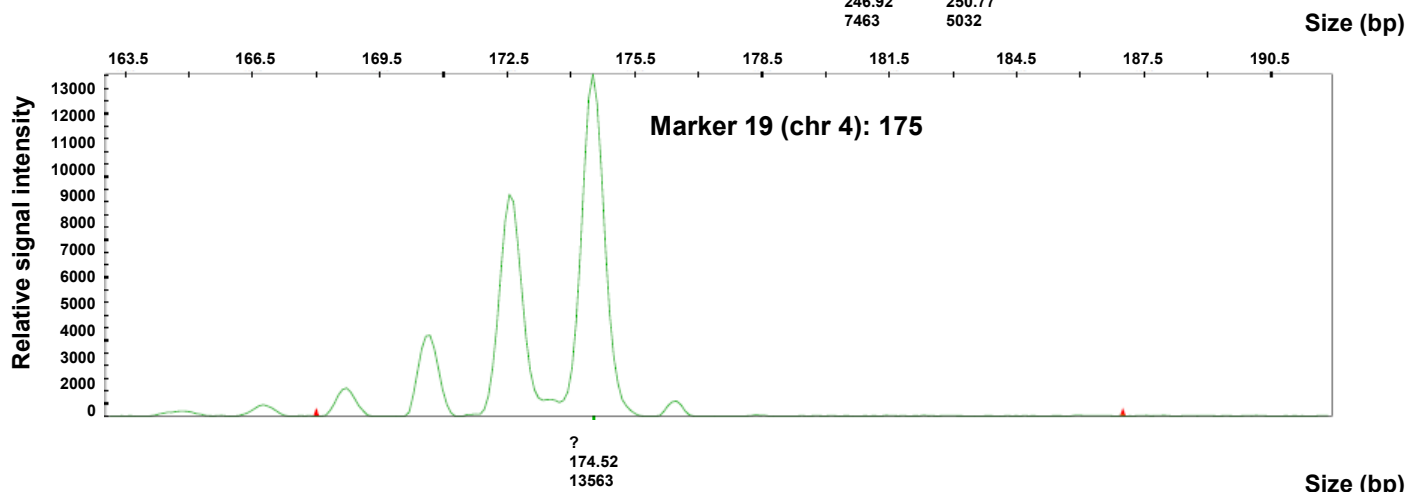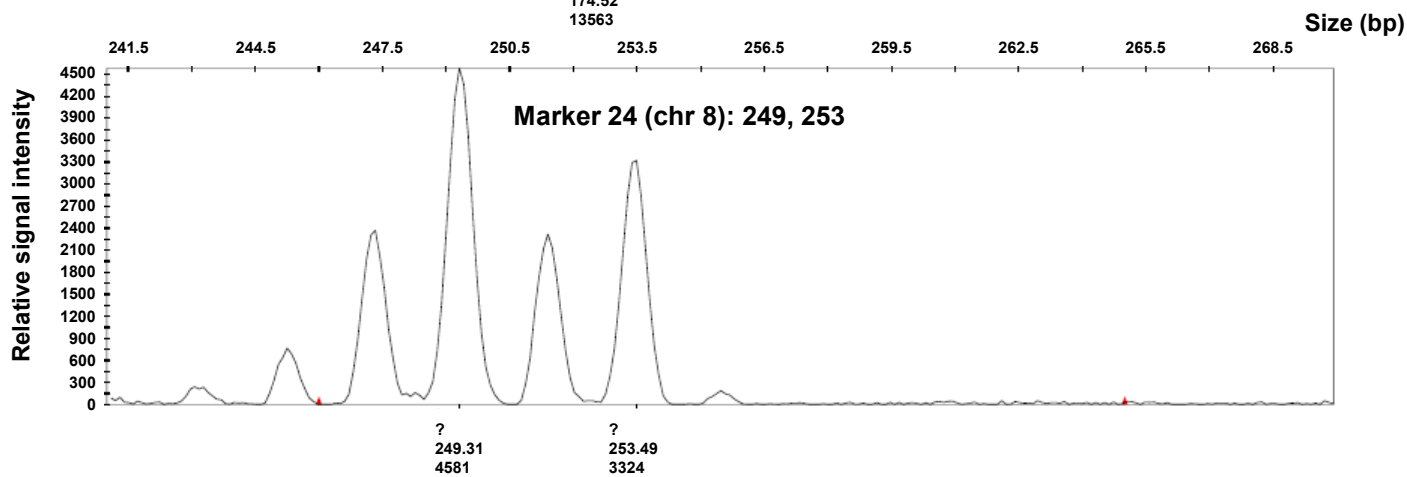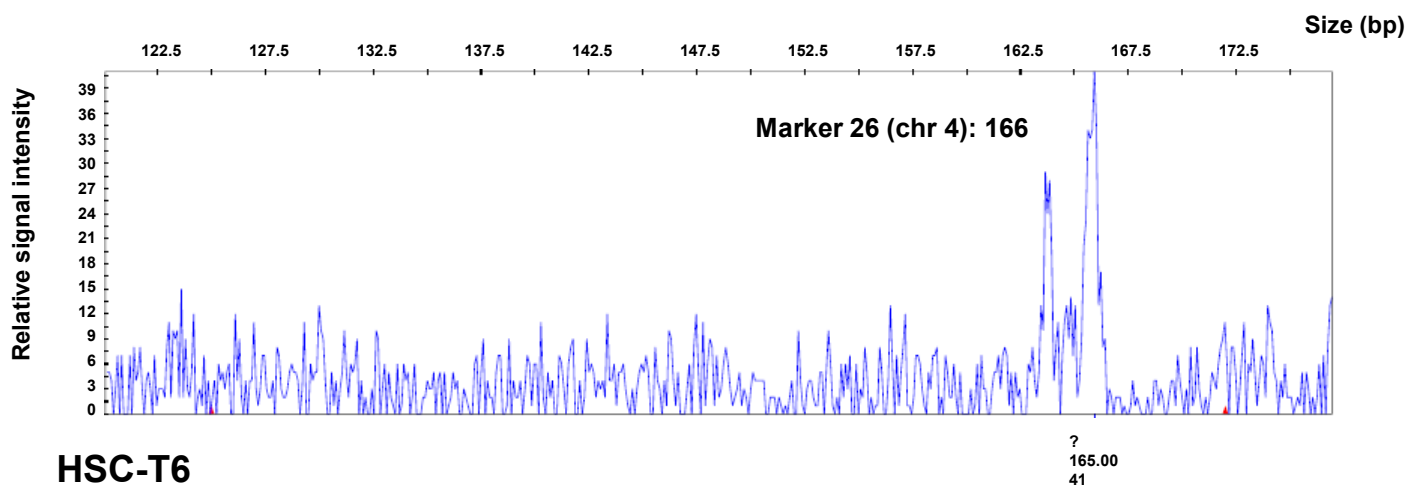

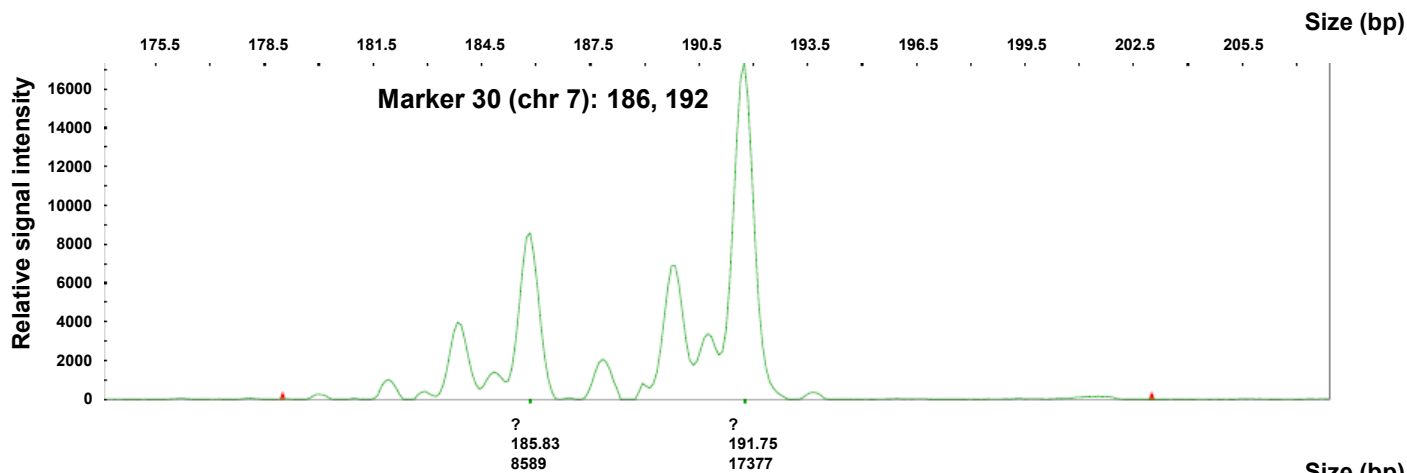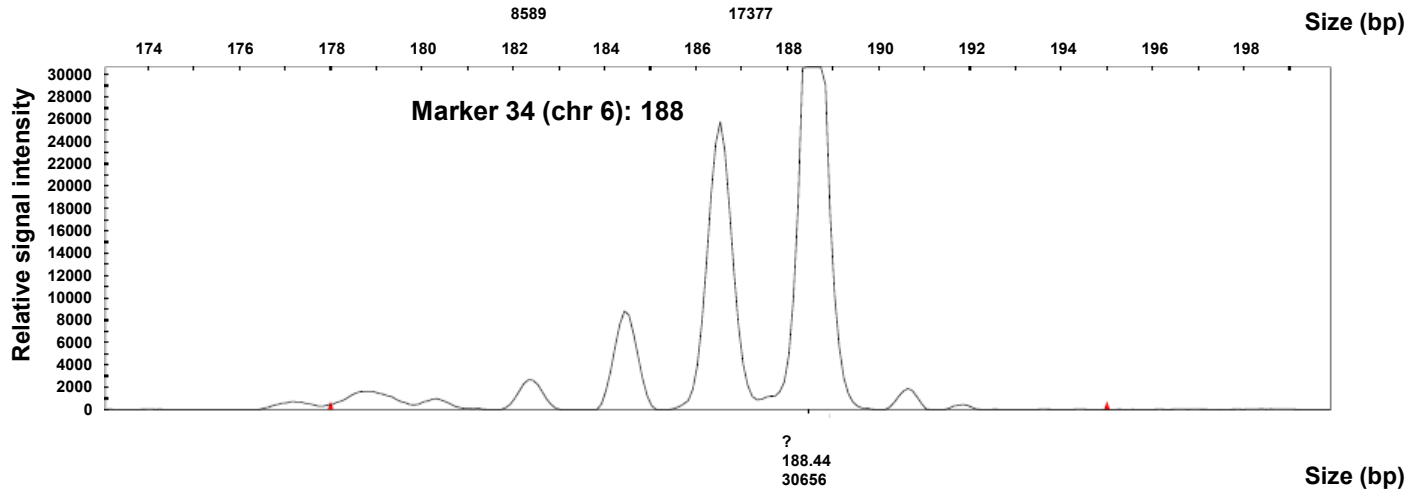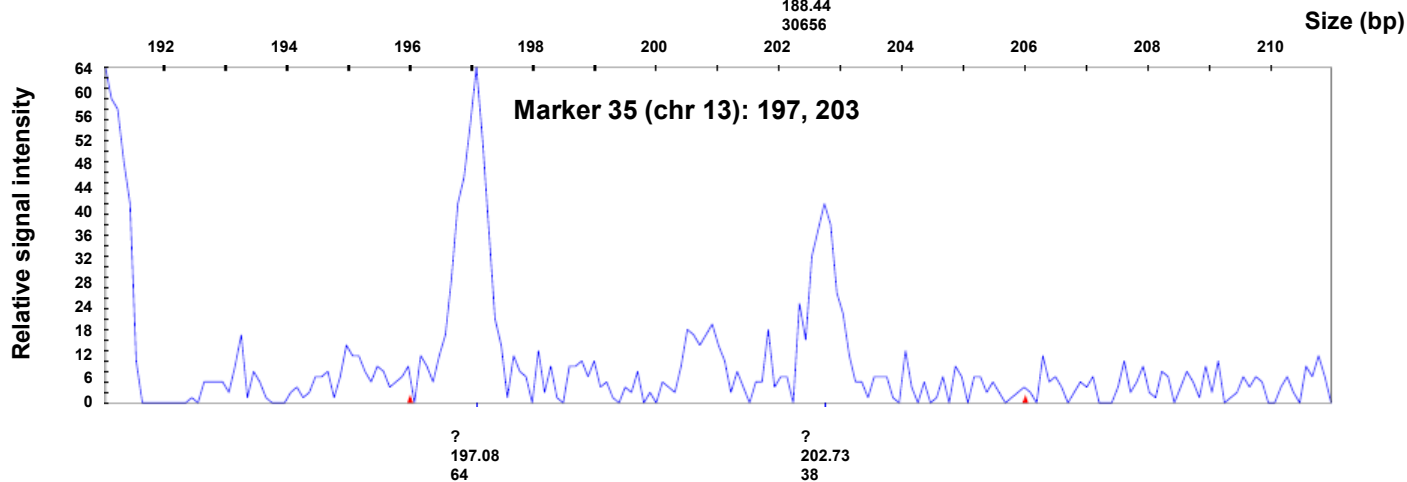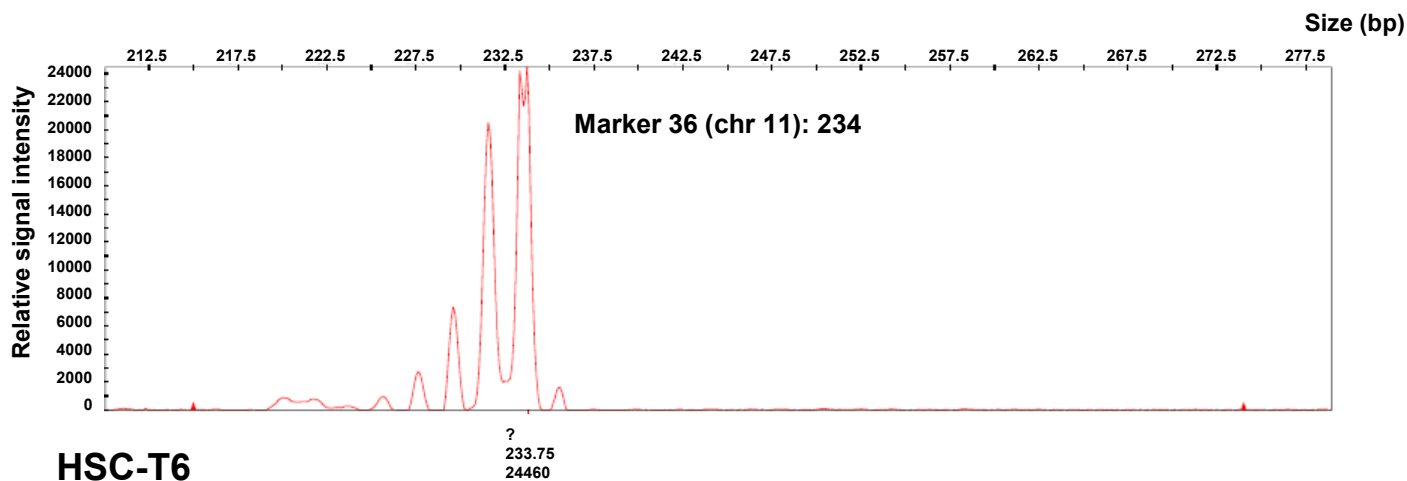

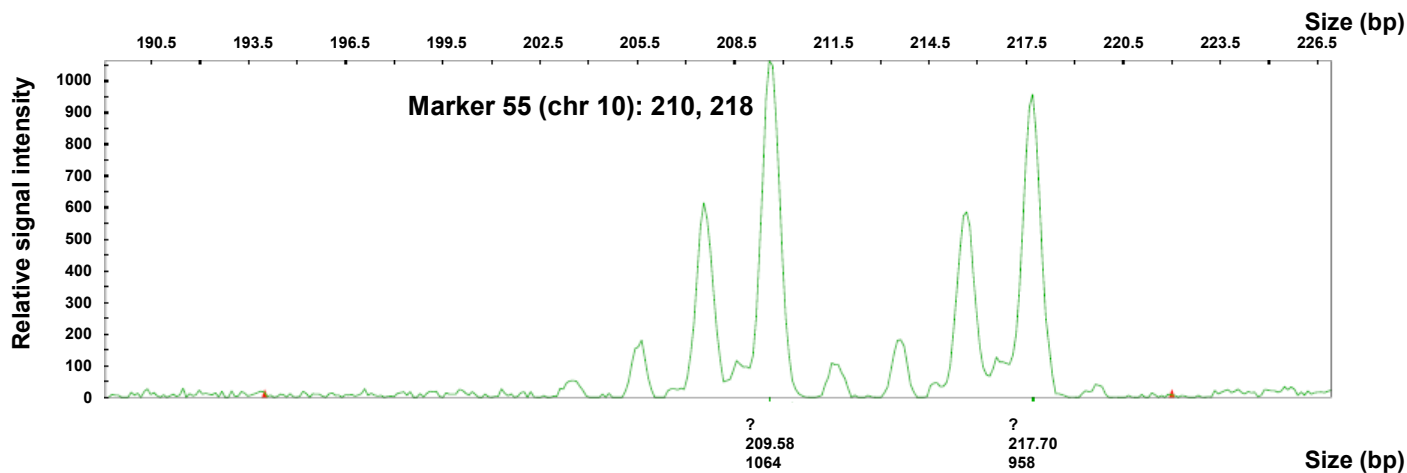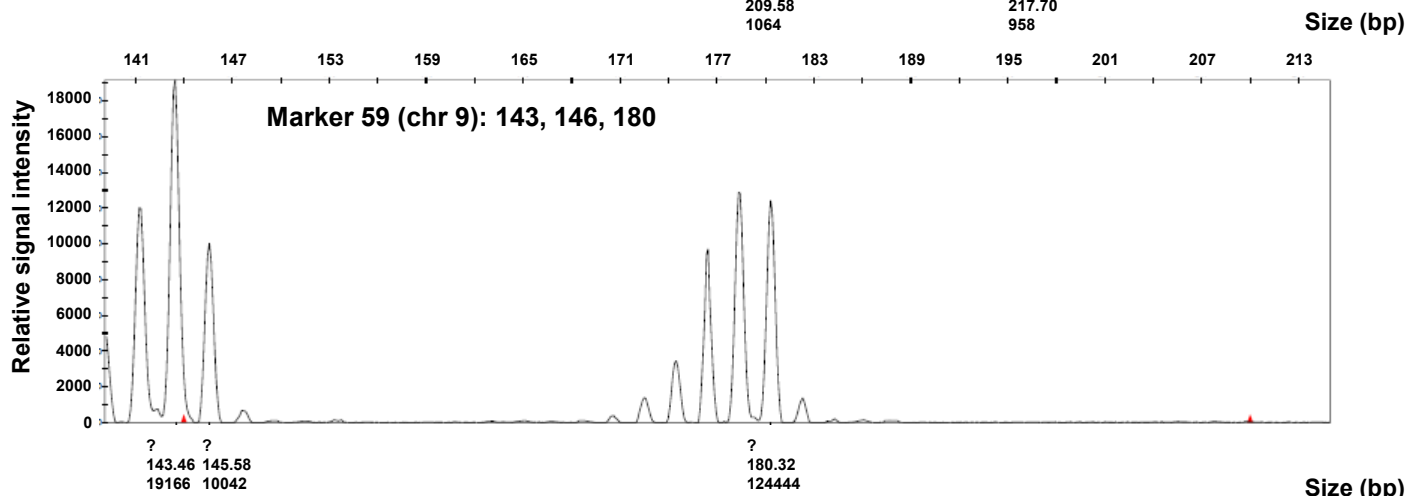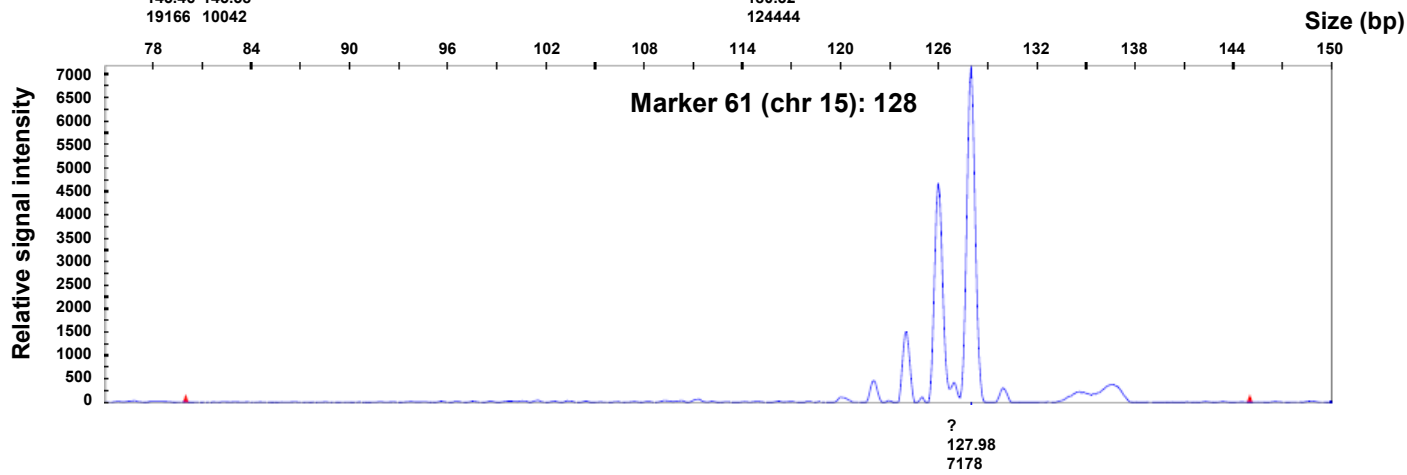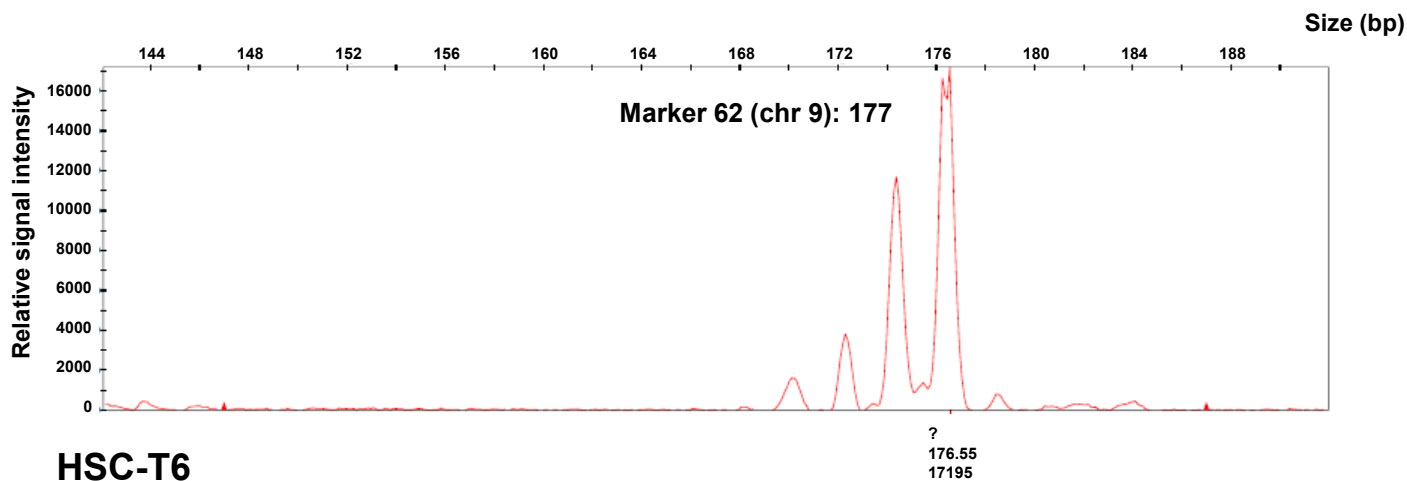

HSC-T6

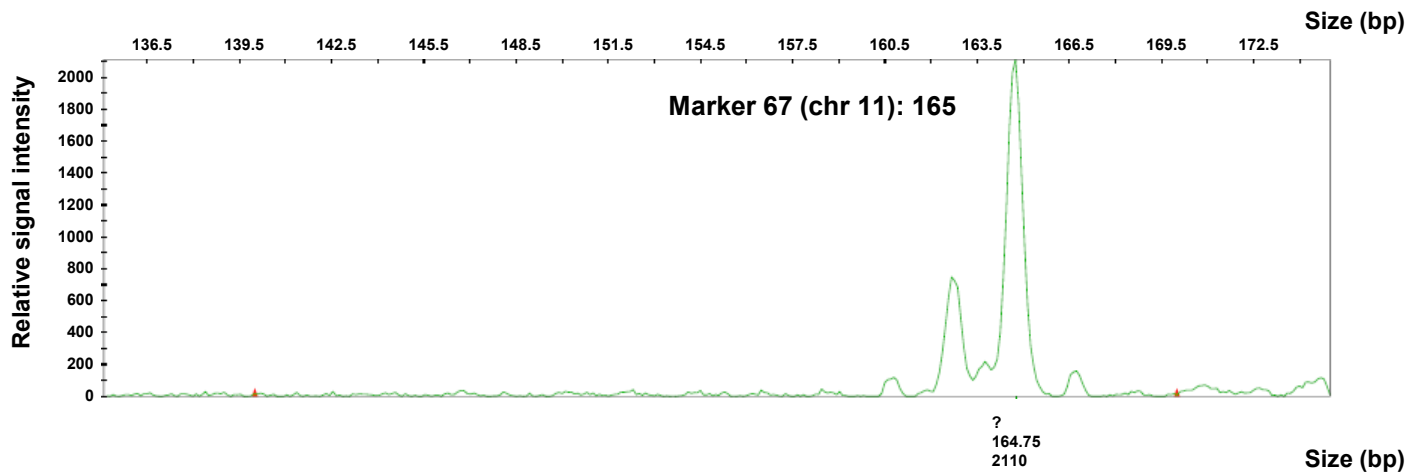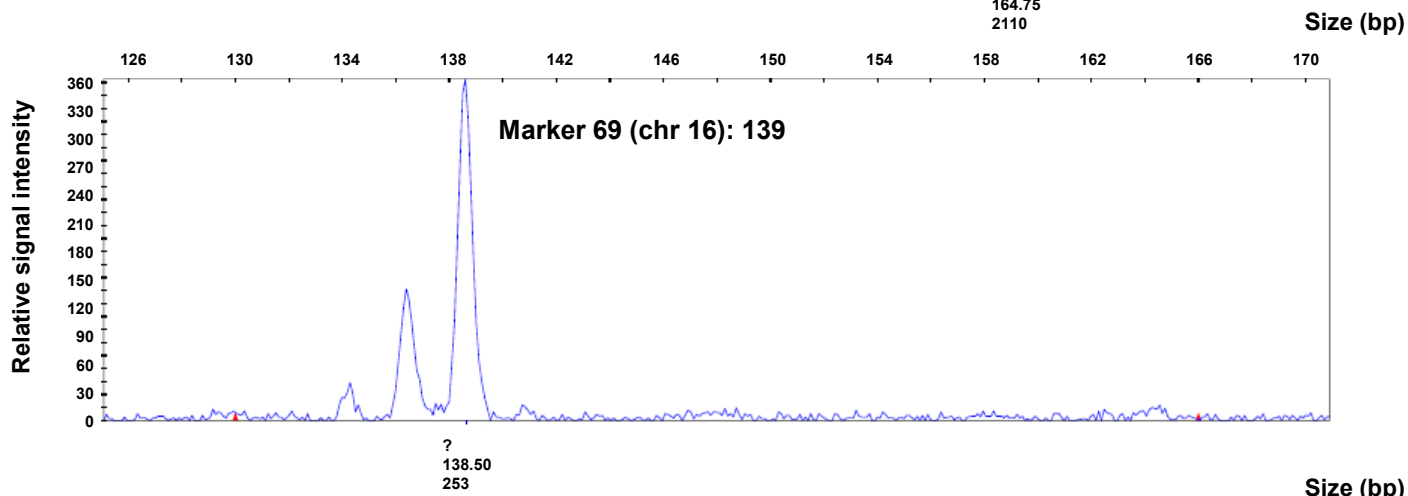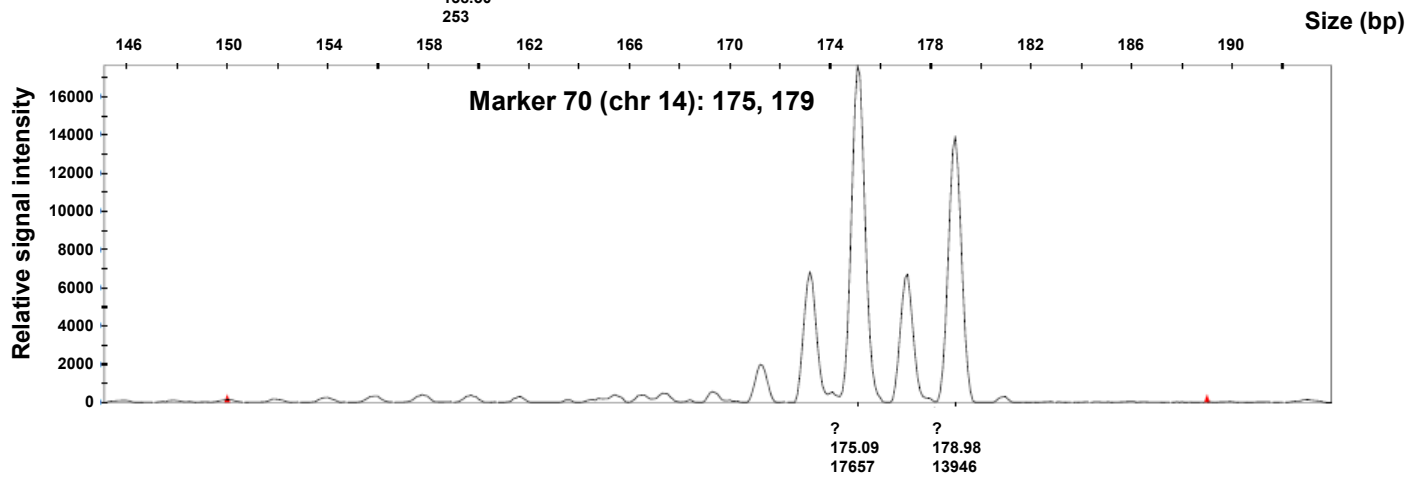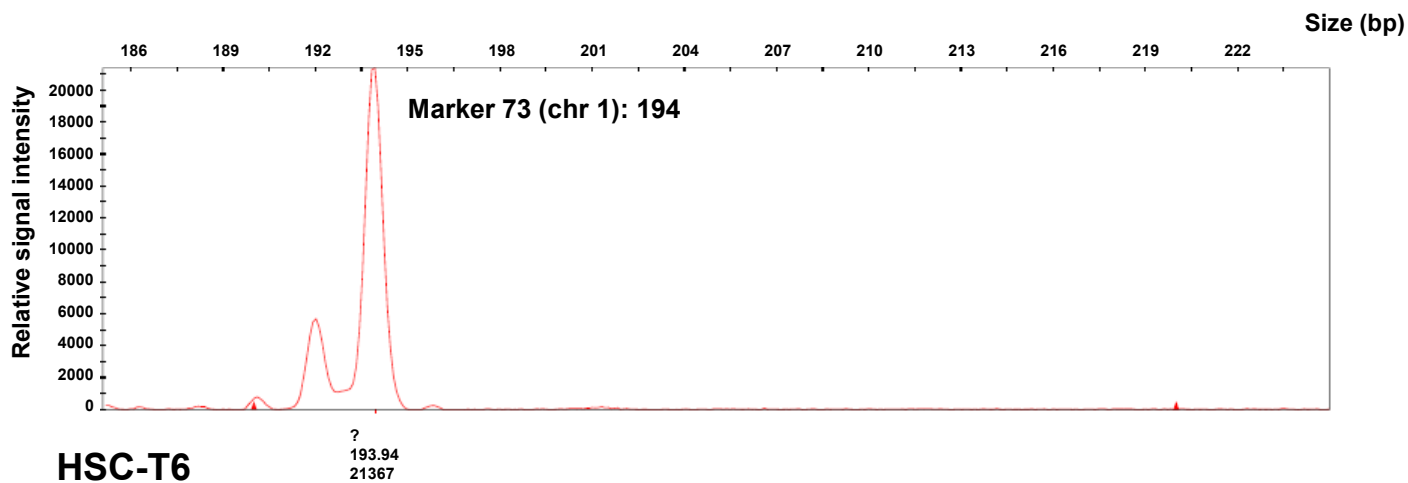

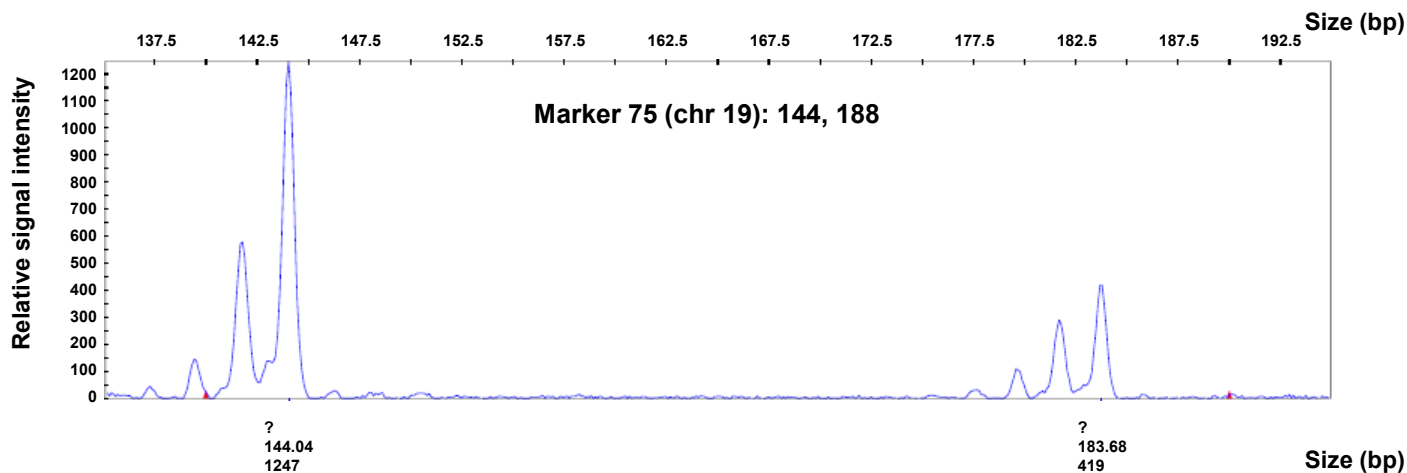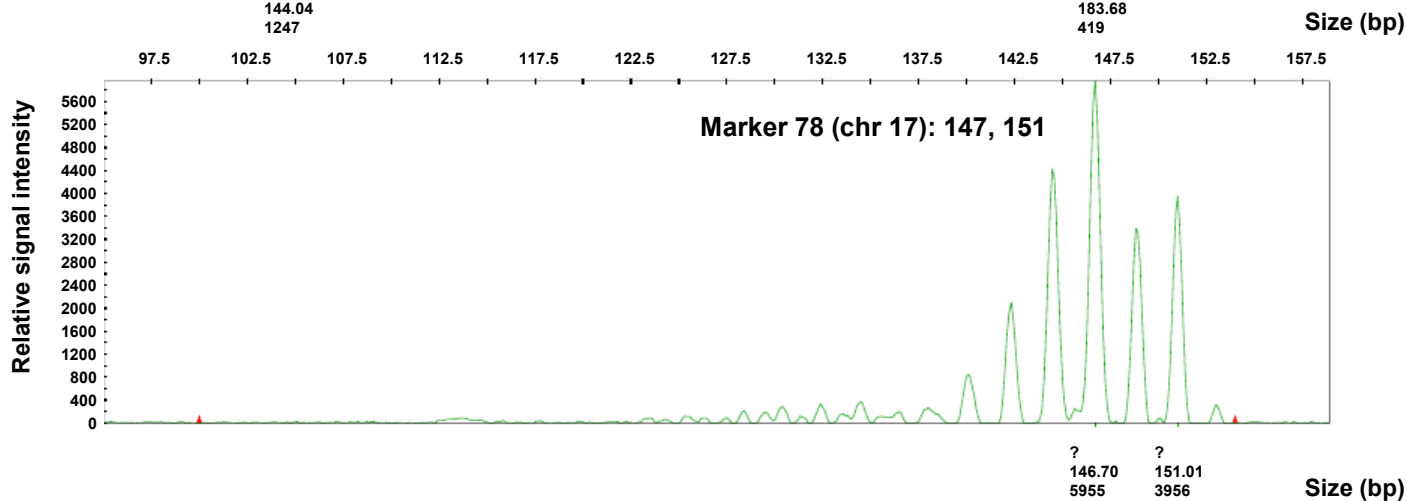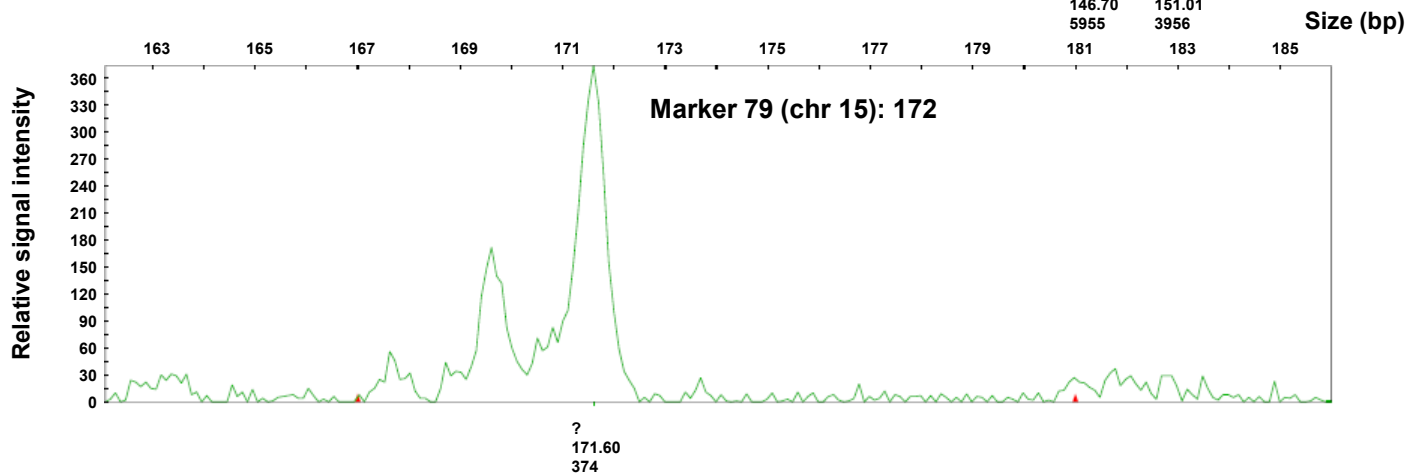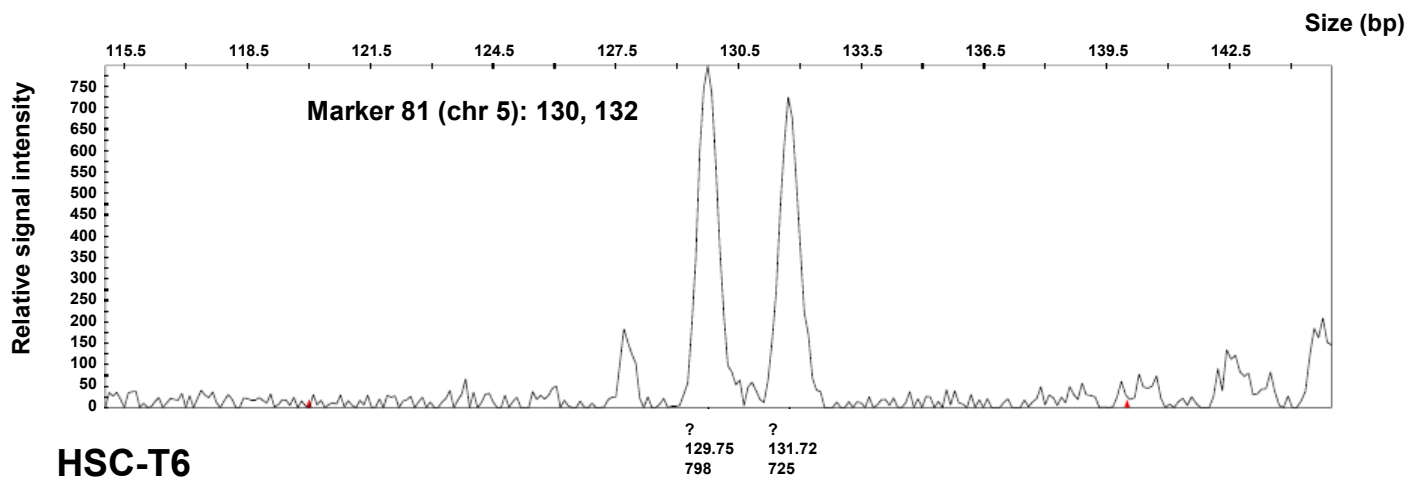

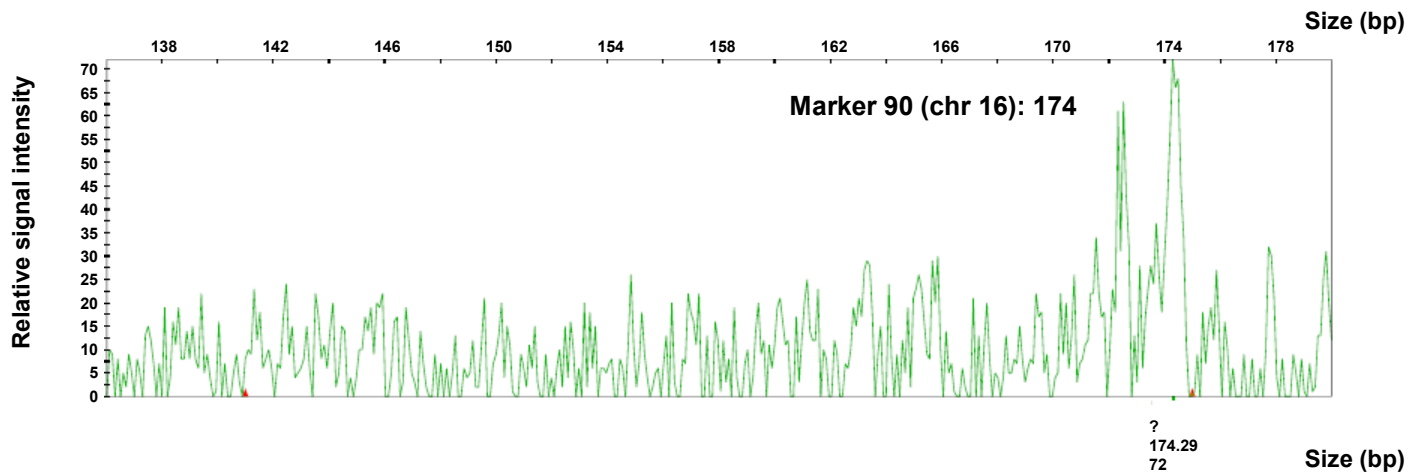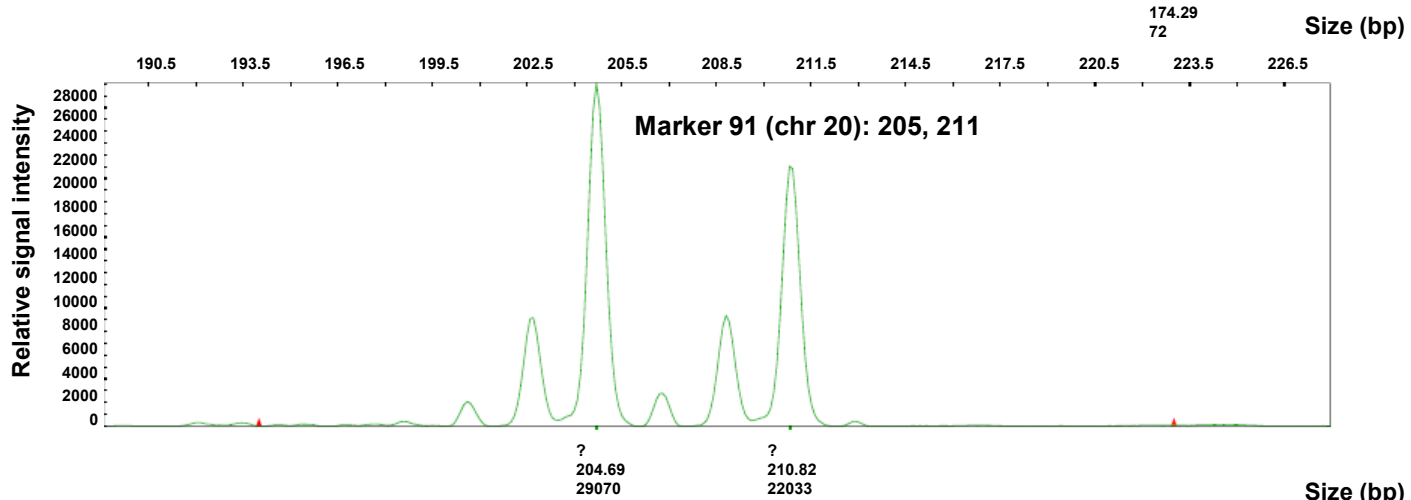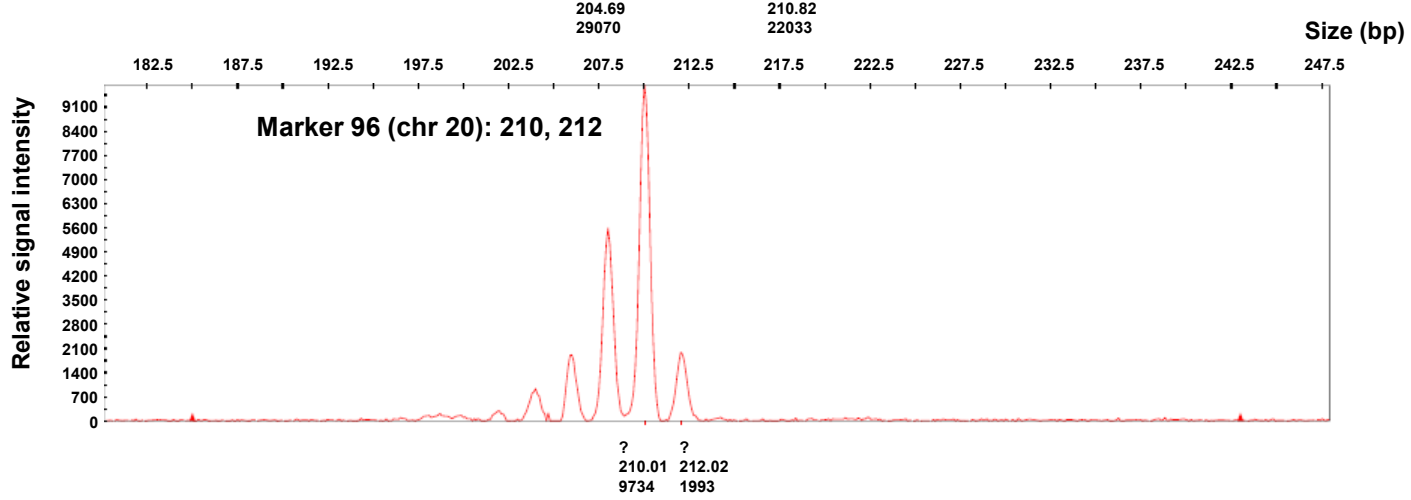

Supplement: Supplementary file 1 [file cells-11-01783-s001.zip › Figure S7.pdf]
